# Copper-catalyzed asymmetric methylation of fluoroalkylated pyruvates with dimethylzinc

**DOI:** 10.3762/bjoc.14.44

**Published:** 2018-03-07

**Authors:** Kohsuke Aikawa, Kohei Yabuuchi, Kota Torii, Koichi Mikami

**Affiliations:** 1 Department of Chemical Science and Engineering, School of Materials and Chemical Technology, Tokyo Institute of Technology, O-okayama, Meguro-ku, Tokyo 152-8552, Japan

**Keywords:** asymmetric methylation, chiral phosphine ligand, copper catalyst, dimethylzinc, trifluoropyruvate

## Abstract

The catalytic asymmetric methylation of fluoroalkylated pyruvates is shown with dimethylzinc as a methylating reagent in the presence of a copper catalyst bearing a chiral phosphine ligand. This is the first catalytic asymmetric methylation to synthesize various α-fluoroalkylated tertiary alcohols with CF_3_, CF_2_H, CF_2_Br, and *n*-C*_n_*F_2_*_n_*_+1_ (*n* = 2, 3, 8) groups in good-to-high yields and enantioselectivities. Axial backbones and substituents on phosphorus atoms of chiral phosphine ligands critically influence the enantioselectivity. Moreover, the methylation of simple perfluoroalkylated ketones is found to be facilitated by only chiral phosphines without copper.

## Introduction

**Scheme 1:**
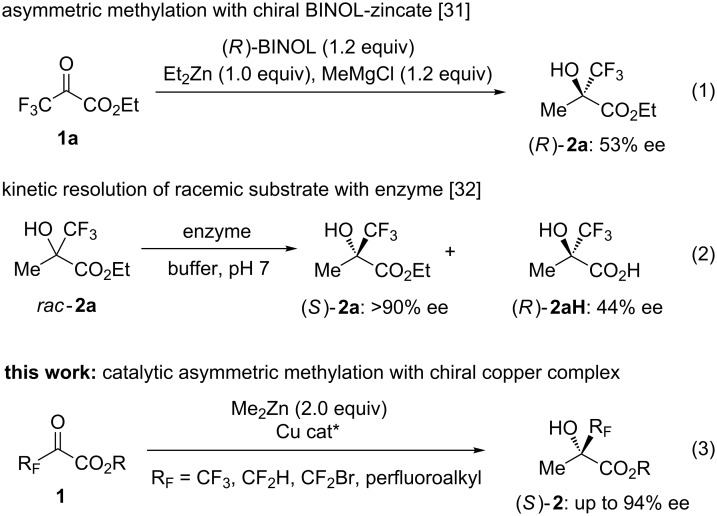
Synthesis of chiral α-fluoroalkylated tertiary alcohols.

The introduction of fluorine atoms into organic compounds plays an important role in the discovery of lead candidates with unique biological and physicochemical properties [1–2]. Therefore, the development of novel synthetic methods for the introduction of fluorinated fragments, such as trifluoromethyl (CF_3_), difluoromethyl (CF_2_H), and difluoromethylene (-CF_2_-), has attracted a great deal of attention from synthetic organic chemists [3–6]. Among these methods, many researchers including us have studied the catalytic asymmetric synthesis of optically active α-trifluoromethylated tertiary alcohols [7–8]. In these cases, one of commercially available and versatile trifluoromethyl sources, trifluoropyruvate, has been utilized for a variety of catalytic asymmetric carbon–carbon bond forming reactions, providing efficiently α-trifluoromethylated tertiary alcohols in high enantioselectivities [9–19]. Over the past decade we have also investigated several catalytic asymmetric reactions using trifluoropyruvate as an electrophile in the presence of a chiral Lewis acid complex [20–27]. However, the synthetic method for chiral α-trifluoromethylated tertiary alcohols via methylation of trifluoropyruvate is quite limited, although several drug candidates bearing this chiral trifluoromethylated moiety have so far been reported [7,28–30]. In 2007, Gosselin and Britton et al. reported that treatment of ethyl trifluoropyruvate (**1a**) with (*R*)-BINOL-mediated organozincate as a chiral methylating regent provided the corresponding methylated tertiary alcohol **2a** in moderate enantioselectivity (Scheme 1, reaction 1) [31]. Kinetic resolution of racemic α-trifluoromethylated tertiary alcohols **2a** by an enzyme is also reported to give the corresponding alcohols **2a** in high enantioselectivity (Scheme 1, reaction 2) [32]. However, there has been no report for catalytic asymmetric methylation of trifluoropyruvate. Herein, we disclose the catalytic asymmetric methylation of trifluoropyruvate derivatives as electrophiles and dimethylzinc as a methylating nucleophile by a chiral copper catalyst. This method is also applicable to the asymmetric synthesis of various α-fluoroalkylated tertiary alcohols bearing CF_2_H, CF_2_Br, and *n*-C*_n_*F_2_*_n_*_+1_ (*n* = 2, 3, 8) groups.

## Results and Discussion

Our initial investigation was focused on the methylation of ethyl trifluoropyruvate (**1a**) with Me_2_Zn in the presence of a copper salt bearing a chiral bidentate phosphine ligand (Table 1). We were delighted to find that the reaction proceeded smoothly in the presence of CuTC (TC: 2-thiophenecarboxylate, 2.5 mol %) and (*R*)-BINAP (2.7 mol %) in Et_2_O at −78 °C, furnishing the methylated product **2a** in 99% yield with 38% ee (Table 1, entry 1). The effect of the Cu salt was also surveyed. The use of CuOAc resulted in slightly decreased enantioselectivities, and (CuOTf)·C_6_H_6_ and CuI led to a racemic product (Table 1, entries 2–4). Chiral phosphine ligands instead of BINAP were further assayed with the aim of enhancing the enantioselectivity. Indeed, the investigation of the effect of axial backbones and substituents on the phosphorus atoms led to an increase in the enantioselectivity. In the case of a biphenyl backbone, MeO-BIPHEP showed the same level of enantioselectivity as BINAP, while lower enantioselectivity was obtained by SEGPHOS (Table 1, entries 5 and 6). Exploring the effect of substituents on phosphorus, DM-BINAP slightly exceeded the level attained by BINAP, although Cy-BINAP and DTBM-BINAP decreased the enantioselectivities (Table 1, entries 7–9). In sharp contrast to BINAP derivatives, DTBM-SEGPHOS and DTBM-MeO-BIPHEP with extremely bulky aryl groups increased the enantioselectivities (Table 1, entries 10 and 11). Additionally, DTB-MeO-BIPHEP provided the desired alcohol in 84% yield with 60% ee (Table 1, entry 12). In toluene and CH_2_Cl_2_ as noncoordinating solvents (Table 1, entries 14 and 15), the reaction gave lower enantioselectivities, but TBME gave the best result in 90% yield and 67% ee (Table 1, entry 16). The use of methyl trifluoropyruvate (**1b**) instead of **1a** resulted in a lower enantioselectivity (Table 1, entry 17). The absolute configuration of **2b** was determined to be *S* by comparison with the optical rotation of reported data [32]. The absolute configurations of other alcohol products **2a** and **2c–k** were tentatively assigned by analogy to **2b**.

**Table 1:** Copper-catalyzed asymmetric methylation.

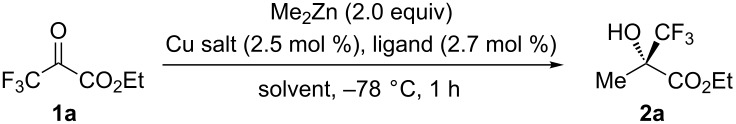					

entry	ligand	Cu salt	solvent	yield (%)^a^	ee (%)

1	(*R*)-BINAP	CuTC	Et_2_O	99	38
2	(*R*)-BINAP	CuOAc	Et_2_O	43	36
3	(*R*)-BINAP	(CuOTf)·C_6_H_6_	Et_2_O	13	0
4	(*R*)-BINAP	CuI	Et_2_O	38	0
5	(*R*)-SEGPHOS	CuTC	Et_2_O	81	26
6	(*R*)-MeO-BIPHEP	CuTC	Et_2_O	85	38
7	(*R*)-Cy-BINAP	CuTC	Et_2_O	92	3
8	(*R*)-DM-BINAP	CuTC	Et_2_O	70	41
9	(*R*)-DTBM-BINAP	CuTC	Et_2_O	73	17
10	(*R*)-DTBM-SEGPHOS	CuTC	Et_2_O	67	55
11	(*R*)-DTBM-MeO-BIPHEP	CuTC	Et_2_O	99	50
12	(*R*)-DTB-MeO-BIPHEP	CuTC	Et_2_O	84	60
13	(*R*)-DTB-MeO-BIPHEP	CuTC	THF	85	57
14	(*R*)-DTB-MeO-BIPHEP	CuTC	toluene	98	38
15	(*R*)-DTB-MeO-BIPHEP	CuTC	CH_2_Cl_2_	90	9
16	(*R*)-DTB-MeO-BIPHEP	CuTC	TBME	90	67
17^b^	(*R*)-DTB-MeO-BIPHEP	CuTC	TBME	71	59 (*S*)

^a^Yields were determined by ^19^F NMR analysis using benzotrifluoride (BTF) as an internal standard. ^b^Methyl trifluoropyruvate (**1b**) was used instead of ethyl trifluoropyruvate (**1a**).
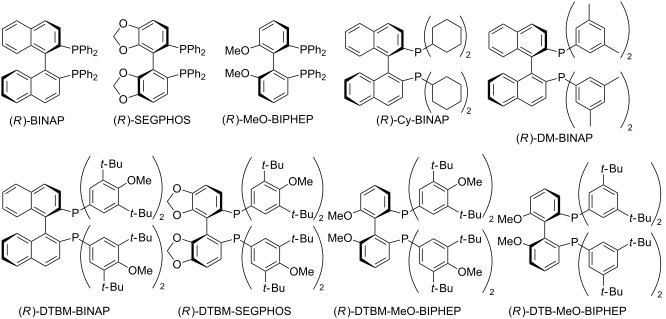

Additionally, the reaction conditions were fine-tuned as exemplified in Table 2. It was found that reactions without CuTC and phosphine ligand (Table 2, entry 1) or only without phosphine ligand (Table 2, entry 2) provided the alcohol as a racemic mixture even at −78 °C. In contrast, the chiral product was obtained in 64% yield in the absence of a copper salt but in low enantioselectivity (Table 2, entry 3). Therefore, decreasing the amount of phosphine ligand (2.4 mol %) to less than that of copper salt led to an enhancement of the enantioselectivity to 70% ee (Table 2, entries 4 vs 5). In addition, the selection of BTFM-Garphos instead of DTB-MeO-BIPHEP afforded a higher enantoselectivity (Table 2, entry 6), and consequently, a lower reaction temperature (−90 °C) gave the best result with 86% yield and 89% ee (Table 2, entry 7).

**Table 2:** Optimization of reaction conditions.

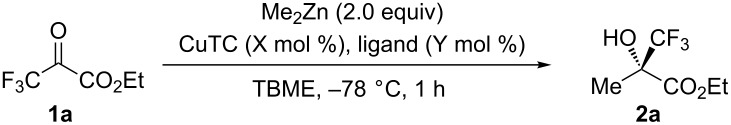					

entry	X (mol %/Cu)	Y (mol %/ligand)	ligand	yield (%)^a^	ee (%)

1	0	0	–	17	–
2	2.5	0	–	15	–
3	0	2.5	(*R*)-DTB-MeO-BIPHEP	64	7
4	2.5	2.7	(*R*)-DTB-MeO-BIPHEP	90	67
5	2.5	2.4	(*R*)-DTB-MeO-BIPHEP	94	70
6	2.5	2.4	(*R*)-BTFM-Garphos	88	73
7^b^	2.5	2.4	(*R*)-BTFM-Garphos	86	89

^a^Yields were determined by ^19^F NMR analysis using benzotrifluoride (BTF) as an internal standard. ^b^Reaction temperature was −90 °C.
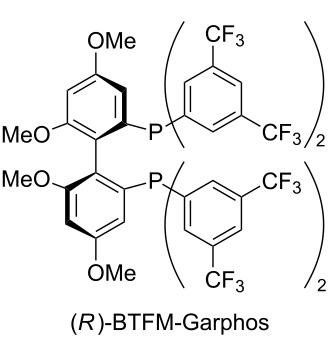

**Scheme 2:**
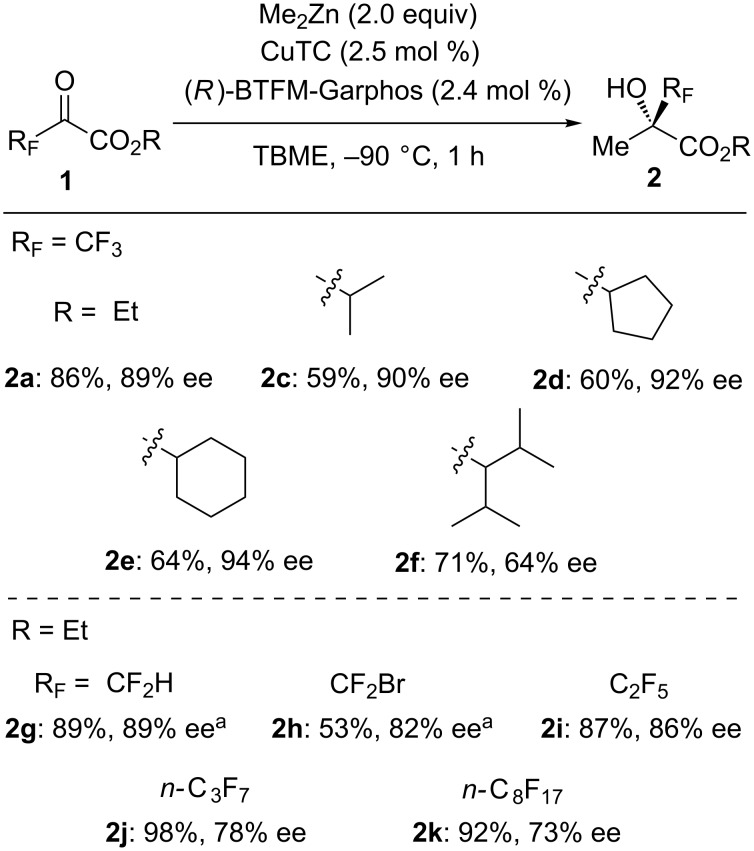
Scope of fluoroalkylated pyruvates. Yields were determined by ^19^F NMR analysis using benzotrifluoride (BTF) as an internal standard. ^a^Reaction temperature was −78 °C.

Various fluoroalkylated pyruvates were applicable to this catalytic transformation under the optimized reaction conditions (Scheme 2). Alkyl substituents on the ester moiety of the trifluoropyruvate were found to influence the stereoselectivity drastically. The reaction of trifluoropyruvates (**1c**–**e**) bearing sterically demanding substituents such as isopropyl, cyclopentyl, and cyclohexyl led to a higher level of enantioselectivities (90–94% ee), compared to the corresponding ethyl ester **1a**. In contrast, trifluoropyruvate **1f** with an extremely bulky substituent caused a decrease of enantioselectivity. Ethyl difluoropyruvate (**1g**) and ethyl bromodifluoropyruvate (**1h**) also underwent the reactions in good enantioselectivities, although a slight decrease in yield was observed due to the steric hindrance of the CF_2_Br group. Significantly, ethyl perfluoropyruvates **1i–k** with longer alkyl chains were also converted to the desired tertiary alcohols in good enantioselectivities.

**Scheme 3:**
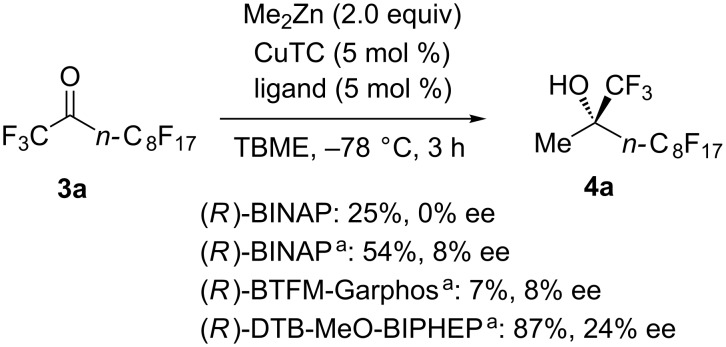
Catalytic asymmetric methylation of the simple perfluoroalkylated ketone **3a**. Yields were determined by ^19^F NMR analysis using benzotrifluoride (BTF) as an internal standard. ^a^Reaction was carried out without CuTC.

The catalytic asymmetric methylation using the simple perfluoroalkylated ketone **3a** instead of pyruvate derivatives was further examined (Scheme 3). In contrast to the pyruvate system, the combination of CuTC and BINAP did not facilitate the reaction even at −78 °C, but also afforded the racemic product (25% yield, 0% ee). Interestingly, the use of only BINAP without CuTC led to higher reactivity and enantioselectivity (54% yield, 8% ee), while BTFM-Garphos decreased the reactivity (7% yield, 8% ee). After screening of phosphines, DTB-MeO-BIPHEP was found to smoothly catalyze the asymmetric methylation to give the desired alcohol **4a** in 87% yield and 24% ee.

## Conclusion

In summary, we have succeeded in the catalytic enantioselective methylation of fluoroalkylated pyruvates in the presence of chiral diphosphine–copper complexes, providing the corresponding tertiary alcohols with an R_F_ group such as CF_3_, CF_2_H, CF_2_Br, and *n*-C*_n_*F_2_*_n_*_+1_ (*n* = 2, 3, 8) in good-to-high yields and enantioselectivities. This is the first report on catalytic asymmetric methylation with fluoroalkylated pyruvates. Moreover, a simple perfluoroalkyl ketone was also found to be methylated enantioselectively with dimethylzinc and a catalytic amount of a chiral diphosphine, but without copper.

## Experimental

**Typical procedure for copper-catalyzed asymmetric methylation of fluoroalkylated pyruvates:** To a mixture of CuTC (1.0 mg, 0.005 mmol) and (*R*)-BTFM-Garphos (5.7 mg, 0.0048 mmol) was added CH_2_Cl_2_ (1.0 mL) at room temperature under an argon atmosphere, and the solution was stirred for 12 h. The solvent was removed under reduced pressure, and the prepared catalyst was dissolved in TBME (0.5 mL) under an argon atmosphere. After the solution was cooled to −90 °C, Me_2_Zn (1.0 M in heptane, 0.4 mL, 0.4 mmol) followed by fluoroalkylated pyruvate **1** (0.2 mmol) in TBME (0.5 mL) were added over 30 min. The reaction mixture was stirred at the same temperature for 1 h. The reaction mixture was quenched with saturated aq NH_4_Cl solution. The organic layer was separated and the aqueous layer was extracted twice with Et_2_O. The combined organic layer was dried over anhydrous Na_2_SO_4_ and evaporated under reduced pressure (350 mmHg). The concentrated solution was used without purification for the next protection reaction. The yield of alcohol product **2** was determined by ^19^F NMR analysis using benzotrifluoride (BTF) as an internal standard.

To a solution of DMAP (2.4 mg, 0.02 mmol) and the crude alcohol **2** in CH_2_Cl_2_ (2.0 mL) was added NEt_3_ (56 μL, 0.4 mmol) at room temperature under an argon atmosphere. After the reaction mixture was cooled to 0 °C, *p-*nitrobenzoyl chloride (56 mg, 0.3 mmol) was added. Then the mixture was warmed to room temperature and stirred for 1 h. After 1 N HCl (5.0 mL) was added to the reaction mixture, the organic layer was separated and the aqueous layer was extracted twice with Et_2_O. The combined organic layer was washed with saturated aq. NaHCO_3_, water, and brine, and then dried over anhydrous MgSO_4_ and evaporated under reduced pressure. The residue was purified by silica gel column chromatography to give *p-*nitrobenzoylated alcohol **2’**. The enantiomeric excess was determined by chiral HPLC analysis.

### (*S*)-3-Ethoxy-1,1,1-trifluoro-2-methyl-3-oxopropan-2-yl 4-nitrobenzoate (**2a’**)

The yield of alcohol **2a** (86%) was determined by ^19^F NMR analysis. *p-*Nitrobenzoylated alcohol **2a’** was purified by silica-gel column chromatography (EtOAc/hexane 1:40) as a colorless liquid (53% yield for 2 steps, 89% ee). ^1^H NMR (300 MHz, CDCl_3_) δ 8.34–8.31 (m, 2H), 8.24–8.20 (m, 2H), 4.33 (q, 4H, *J* = 6.9 Hz), 1.97 (d, 3H, *J* = 0.9 Hz), 1.28 (t, 3H, *J* = 7.0 Hz); ^13^C NMR (75 MHz, CDCl_3_) δ 164.3, 162.3, 151.1, 134.0, 131.2, 123.7, 122.7 (q, *J*_ C-F_ = 282.9 Hz), 80.7 (q, *J*_ C-F_ = 30.4 Hz), 63.2, 16.6, 13.8; ^19^F NMR (282 MHz, CDCl_3_) δ −78.4 (s, 3F); HRMS (APCI-TOF): [M]^−·^ calcd for C_13_H_12_F_3_NO_6_, 335.0617; found, 335.0623; FTIR (neat, cm^−1^) 784, 813, 849, 876, 927, 1011, 1109, 1149, 1273, 1342, 1387, 1452, 1525, 1602, 1740, 1763, 2857, 2920, 2952, 2996, 3087, 3116; [α]_D_^22^ −28.94 (*c* 0.20, CHCl_3_); HPLC (column, CHIRALCEL OJ-3, hexane/2-propanol 91:9, flow rate 0.6 mL/min, 20 °C detection UV 254 nm) *t*_R_ of major isomer 13.1 min, *t*_R_ of minor isomer 23.8 min.

### (*S*)-1-Ethoxy-3,3-difluoro-2-methyl-1-oxopropan-2-yl benzoate (**2g’**)

Reaction temperature was −78 °C. The yield of alcohol **2g** (89%) was determined by ^19^F NMR analysis. In the protection of alcohol, benzoyl chloride was used instead of *p*-nitrobenzoyl chloride. Benzoylated alcohol **2g’** was purified by silica gel column chromatography (EtOAc/hexane 1:40) as a colorless liquid (41% yield for 2 steps, 89% ee). ^1^H NMR (300 MHz, CDCl_3_) δ 8.05 (dd, *J* = 8.3, 1.3 Hz, 2H). 7.61 (tt, *J* = 6.7, 1.3 Hz, 1H), 7.49–7.44 (m, 2H), 6.30 (dd, *J*_H-F_ = 56.8, 54.8 Hz, 1H), 4.29 (q, *J* = 7.1 Hz, 2H), 1.77 (t, *J*_H-F_ = 1.6 Hz, 3H), 1.27 (t, *J* = 7.1 Hz, 3H); ^13^C NMR (75 MHz, CDCl_3_) δ 167.6, 164.8, 133.7, 130.0, 128.8, 128.5, 122.9 (dd, *J*_C-F_ = 250.0, 245.0 Hz), 79.7 (dd, *J*_C-F_ = 27.5, 21.9 Hz), 62.3, 14.6 (t, *J*_C-F_ = 3.2 Hz) 13.9; ^19^F NMR (282 MHz, CDCl_3_) δ −128.40 (dd, *J* = 290.2 Hz, *J*_F-H_ =54.7 Hz, 1F), −132.76 (dd, *J* = 289.90 Hz, *J*_F-H_ = 56.4 Hz, 1F); HRMS (APCI-TOF): [M + Na]^+^ calcd for C_13_H_14_F_2_NaO_4_, 295.0758; found, 295.0761; FTIR (neat, cm^−1^) 1026, 1093, 1114, 1216, 1279, 1388, 1452, 1602, 1730, 1747, 2938, 2985, 3021; [α]_D_^25^ −7.47 (*c* 1.01, CHCl_3_); HPLC (column, CHIRALCEL OJ-3, hexane/2-propanol 99:1, flow rate 0.6 mL/min, 20 °C detection UV 220 nm) *t*_R_ of major isomer 21.2 min, *t*_R_ of minor isomer 22.6 min.

### (*S*)-1-Bromo-3-ethoxy-1,1-difluoro-2-methyl-3-oxopropan-2-yl 4-nitrobenzoate (**2h’**)

Reaction temperature was −78 °C. The yield of alcohol **2h** (53%) was determined by ^19^F NMR analysis. *p*-Nitrobenzoylated alcohol **2h’** was purified by silica gel column chromatography (EtOAc/hexane 1:50) as a white solid (32% yield for 2 steps, 82% ee). ^1^H NMR (300 MHz, CDCl_3_) δ 8.34–8.31 (m, 2H), 8.25–8.21 (m, 2H), 4.32 (q, 2H, *J* = 7.2 Hz), 2.02 (s, 3H), 1.29 (t, 3H, *J* = 7.0 Hz); ^13^C NMR (75 MHz, CDCl_3_) δ 164.3, 162.4, 151.2, 134.3, 131.3, 123.9, 121.0 (t, *J*_C-F_ = 311.6 Hz), 84.8 (dd, *J*_C-F_ = 25.6, 23.4 Hz), 63.4, 18.4, 14.0; ^19^F NMR (282 MHz, CDCl_3_) δ −56.9 (d, 1F, *J* = 168.6 Hz), −58.9 (d, 1F, *J* = 165.3 Hz); HRMS (APCI-TOF): [M]^−·^ calcd for C_13_H_12_BrF_2_NO_6_, 394.9816; found, 394.9835; FTIR (KBr pellet, cm^−1^) 716, 843, 876, 961, 1020, 1106, 1146, 1280, 1347, 1446, 1528, 1610, 1751, 2866, 2936, 2988; [α]_D_^22^ −11.99 (*c* 1.55, CHCl_3_); HPLC (column, CHIRALCEL OD-3, hexane/2-propanol 91:9, flow rate 0.6 mL/min, 20 °C detection UV 254 nm) *t*_R_ of major isomer 18.2 min, *t*_R_ of minor isomer 12.5 min.

### (*S*)-1-Ethoxy-3,3,4,4,4-pentafluoro-2-methyl-1-oxobutan-2-yl *p*-nitrobenzoate (**2i’**)

The yield of alcohol **2i** (87%) was determined by ^19^F NMR analysis. *p*-Nitrobenzoylated alcohol **2i’** was purified by silica gel column chromatography (EtOAc/hexane 1:40) as a white solid (48% yield for 2 steps, 86% ee). ^1^H NMR (300 MHz, CDCl_3_) δ 8.35–8.30 (m, 2H) 8.21–8.16 (m, 2H), 4.37–4.27 (m, 2H), 2.04 (q, *J*_H-F_ = 0.6 Hz, 3H), 1.28 (t, *J* = 7.1 Hz, 3H); ^13^C NMR (75 MHz, CDCl_3_) δ 164.2, 162.2 (d, *J*_C-F_ = 2.0 Hz), 151.0, 134.1, 131.0, 123.8, 118.6 (qt, *J*_C-F_ = 286.1, 35.6 Hz), 112.0 (tq, *J*_C-F_ = 263.0, 36.8 Hz), 81.3 (t, *J*_C-F_ = 25.4 Hz), 63.3, 16.6, 13.7; ^19^F NMR (282 MHz, CDCl_3_) δ −79.19 (s, 3F), −121.42 (d, *J* = 280.9 Hz, 1F), −122.98 (d, *J* = 279.7 Hz, 1F); HRMS (APCI-TOF): [M]^−·^ calcd for C_14_H_12_F_5_NO_6_, 385.0585; found, 385.0582; FTIR (KBr pellet, cm^−1^) 1014, 1142, 1208, 1222, 1281, 1350, 1385, 1533, 1747, 2942, 2987, 3059; [α]_D_^25^ −27.75 (*c* 1.02, CHCl_3_); HPLC (column, CHIRALCEL OJ-3, hexane/2-propanol 99:1, flow rate 0.6 mL/min, 20 °C detection UV 220 nm) *t*_R_ of major isomer 16.2 min, *t*_R_ of minor isomer 31.4 min.

### (*S*)-1-Ethoxy-3,3,4,4,5,5,5-heptafluoro-2-methyl-1-oxopentan-2-yl *p*-nitrobenzoate (**2j’**)

The yield of alcohol **2j** (98%) was determined by ^19^F NMR analysis. *p*-Nitrobenzoylated alcohol **2j’** was purified by silica-gel column chromatography (EtOAc/hexane 1:50) as a colorless oil (48% yield for 2 steps, 78% ee). ^1^H NMR (300 MHz, CDCl_3_) δ 8.35–8.31 (m, 2H) 8.21–8.16 (m, 2H), 4.36–4.29 (m, 2H), 2.07 (q, *J*_H-F_ = 1.3 Hz, 3H), 1.28 (t, *J* = 7.1 Hz, 3H); ^13^C NMR (75 MHz, CDCl_3_) δ 164.2 162.2, 151.0, 134.1, 131.0, 123.8, 117.6 (qt, *J*_C-F_ = 286.9, 33.9 Hz), 113.6 (tt, *J*_C-F_ = 263.9, 30.9 Hz), 122.9 (tq, *J*_C-F_ = 266.9, 37.4 Hz), 82.2 (t, *J*_C-F_ = 25.7 Hz), 63.4, 16.8, 13.7; ^19^F NMR (282 MHz, CDCl_3_) δ −80.65 (m, 3F), −117.75 (d, *J* = 288.5 Hz, 1F), −119.60 (d, *J* = 288.2 Hz, 1F), −123.882 (s, 2F); HRMS (APCI-TOF): [M]^−·^ calcd for C_15_H_12_F_7_NO_6_, 435.0553; found, 435.0547; FTIR (neat, cm^−1^) 1090, 1140, 1200, 1233, 1349, 1387, 1534, 1609, 1744, 1761, 2942, 2988, 3059; [α]_D_^25^ −22.60 (*c* 0.94, CHCl_3_); HPLC (column, CHIRALCEL OJ-3, hexane/2-propanol 99:1, flow rate 0.6 mL/min, 20 °C detection UV 220 nm) *t*_R_ of major isomer 12.2 min, *t*_R_ of minor isomer 15.5 min.

### (*S*)-1-Ethoxy-3,3,4,4,5,5,6,6,7,7,8,8,9,9,10,10,10-heptadecafluoro-2-methyl-1-oxodecan-2-yl *p*-nitrobenzoate (**3k’**)

The yield of alcohol **2k** (92%) was determined by ^19^F NMR analysis. *p*-Nitrobenzoylated alcohol **2k’** was purified by silica-gel column chromatography (EtOAc/hexane 1:50) as a white solid (85% yield for 2 steps, 73% ee). ^1^H NMR (300 MHz, CDCl_3_) δ 8.36–8.31 (m, 2H), 8.20–8.16 (m, 2H), 4.36–4.29 (m, 2H), 2.08 (s, 3H), 1.28 (t, 3H, *J* = 7.3 Hz); ^13^C NMR (75 MHz, CDCl_3_) δ 164.3, 162.4, 151.2, 134.3, 131.2, 123.9, 118.4–104.7 (m), 117.3 (qt, *J*_C-F_ = 288.4, 33.2 Hz), 82.8 (t, J_C-F_ = 25.4 Hz), 63.5, 17.1, 13.8; ^19^F NMR (282 MHz, CDCl_3_) δ –80.7 (m, 3F), –116.4 to −126.0 (m, 14F); HRMS (APCI-TOF): [M]^−·^ calcd for C_20_H_12_F_17_NO_6_, 685.0393; found, 685.0362; FTIR (KBr pellet cm^−1^) 847, 969, 1009, 1142, 1214, 1246, 1297, 1472, 1530, 1613, 1732, 1757, 2339, 2360, 2860, 2922, 2997, 3112, 3454, 3493; [α]_D_^22^ −11.93 (*c* 0.48, CHCl_3_); HPLC (column, CHIRALPAK AD-3 and AD-H, hexane/2-propanol 99.5:0.5, flow rate 0.6 mL/min, 20 °C detection UV 254 nm) *t*_R_ of major isomer 16.8 min, *t*_R_ of minor isomer 24.4 min.

## Supporting Information

File 1 Experimental details and characterization data of new compounds with copies of ^1^H, ^13^C and ^19^F NMR spectra.
