# Supplementary material for: Copper-catalyzed asymmetric methylation of fluoroalkylated pyruvates with dimethylzinc
Source: Beilstein J Org Chem. 2018 Mar 7;14:576–82. doi: 10.3762/bjoc.14.44 (PMC5852614; doi:10.3762/bjoc.14.44)
Supplement: File 1 — Experimental details and characterization data of new compounds with copies of 1H, 13C and 19F NMR spectra. [file Beilstein_J_Org_Chem-14-576-s001.pdf]

Supporting Information

for

Copper-catalyzed asymmetric methylation of fluoroalkylated pyruvates with dimethylzinc

Kohsuke Aikawa, Kohei Yabuuchi, Kota Torii and Koichi Mikami*

Address: Department of Chemical Science and Engineering, School of Materials and Chemical Technology, Tokyo Institute of Technology, O-okayama, Meguro-ku, Tokyo 152-8552, Japan

Email: Koichi Mikami* - mikami.k.ab@m.titech.ac.jp,

*Corresponding author

Experimental details and characterization data of new compounds with copies of ^{1}H, ^{13}C and ^{19}F NMR spectra

Table of Contents

General information .... page S2
Experimental procedures
Typical procedure I: synthesis of dialkyl oxalate .... page S3–S4
Typical procedure II: synthesis of trifluoropyruvate derivatives .... page S4–S6
Typical procedure III:
Cu-catalyzed asymmetric methylation of fluoroalkylated pyruvate .... page S7–S12
Typical procedure IV:
Cu-catalyzed asymmetric methylation of trifluoropyruvate derivatives .... page S13–S14
Determination of absolute configuration .... page S14–S15
Synthesis of simple perfluoroalkylated ketone .... page S15
Catalytic asymmetric methylation of perfluoroalkylated ketone .... page S16–S17
References .... page S17
NMR spectra .... page S18–S40

General information:

^{1}H, ^{13}C, and ^{19}F NMR spectra were measured on a Bruker AV300M (300 MHz) spectrometer. Chemical shifts of ^{1}H NMR were expressed in parts per million relative to the singlet (δ = 7.26) for $CHCl_3$ as internal standard in $CDCl_3$. Chemical shifts of ^{13}C NMR were expressed in parts per million relative to the central line of the triplet (δ = 77.0) for $CDCl_3$. Chemical shifts of ^{19}F NMR were expressed in parts per million relative to the singlet (δ = −63.24) for BTF (benzotrifluoride) as an internal standard. Optical rotations were measured on JASCO P-1020. Mass spectra were measured on a JEOL JMS-T100CS (Accu-TOF) spectrometer. IR spectra were measured on a JASCO FT/IR-4200 spectrometer. High performance liquid chromatography (HPLC) was conducted on JASCO PU-980, LG-980-02, DG-980-50, MD-2010, and CO-966 instrument equipped with model UV-975 spectrometers as an ultra violet light. Peak areas were calculated by JASCO chrom NAV (Windows 7) as an automatic integrator. All experiments were carried out under argon atmosphere unless otherwise noted.

Copper(I) thiophene-2-carboxylate (CuTC), Me_2Zn (1 M in heptane solution), and *tert*-butyl methyl ether (dehydrate) were purchased from Aldrich. (*R*)-BTFM-Garphos was purchased from Strem Chemicals, Inc.

Substrates:

Ethyl trifluoropyruvate **1a** and was provided from Central Glass Co., Ltd. Methyl trifluoropyruvate **1b** was purchased from TCI. Ethyl difluoropyruvate **1g** [1], ethyl bromodifluoropyruvate **1h** [2], and ethyl perfluoroalkylpyruvates **1i–k** [3] were synthesized according to published procedure.

Typical procedure I: synthesis of dialkyl oxalate

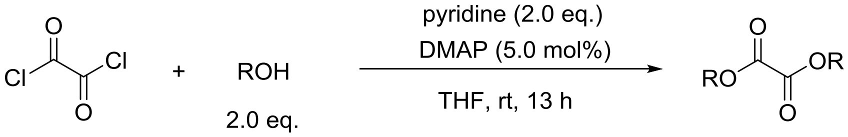

To an oven-dried 200-mL two-neck round-bottomed flask equipped with magnetic stir bar were added *N*,*N*'-dimethylaminopyridine (183 mg, 1.5 mmol), pyridine (4.84 mL, 60 mmol), ROH (60 mmol), and THF (200 mL). Oxalyl chloride (3.3 mL, 30 mmol) was added dropwise to the solution. After stirring at room temperature for 13 h, the reaction mixture was diluted with H_2O (100 mL) and extracted with Et_2O (3 × 50 mL). The combined organic layers were washed with saturated aq. $NaHCO_3$ (100 mL), water (100 mL), brine (100 mL), and dried over $MgSO_4$, and then filtered and concentrated under reduced pressure. The residue was purified by silica gel column chromatography to give the corresponding dialkyl oxalate.

Diisopropyl oxalate [4]

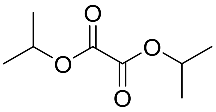

The compound was purified by silica gel column chromatography (EtOAc/hexane 1:20) as a colorless liquid (76% yield). The product is known compound, the following data are identical to those given in corresponding literature [5].
^{1}H NMR (300 MHz, $CDCl_3$) δ 5.10 (sept, 2H, *J* = 6.3 Hz), 1.30 (d, 12H, *J* = 6.3 Hz); ^{13}C NMR (75 MHz, $CDCl_3$) δ 157.9, 71.3, 21.5.

Dicyclopentyl oxalate

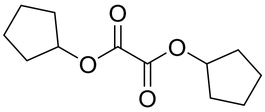

The compound was purified by silica-gel column chromatography (EtOAc/hexane 1:20) as a colorless liquid (91% yield).
^{1}H NMR (300 MHz, $CDCl_3$) δ 5.25-5.19 (m, 2H), 1.92-1.80 (m, 4H), 1.78-1.66 (m, 8H), 1.62-1.49 (m, 4H); ^{13}C NMR (75 MHz, $CDCl_3$) δ 158.1, 80.1, 32.4, 23.6; HRMS (ESI-TOF) calcd for $C_{12}H_{18}NaO_4$ $[M+Na]^+$: 249.1103, found: 249.1106; FT-IR (neat, cm^{-1}) 762, 852, 942, 1017, 1122, 1172, 1310, 1363, 1465, 1747, 1763, 2875, 2943, 2974.

Dicyclohexyl oxalate

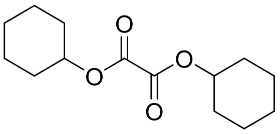

The compound was purified by silica-gel column chromatography (EtOAc/hexane 1:20) as white solid (92% yield).

^{1}H NMR (300 MHz, $CDCl_3$) δ 4.94-4.85 (m, 2H), 1.93-1.88 (m, 4H), 1.81-1.74 (m, 4H), 1.60-1.48 (m, 6H), 1.44-1.20 (m, 6H); ^{13}C NMR (75 MHz, $CDCl_3$) δ 157.9, 76.0, 31.3, 25.3, 23.7; HRMS (ESI-TOF) calcd for $C_{14}H_{22}NaO_4$ $[M+Na]^+$: 277.1416, found: 277.1429; FT-IR (KBr, cm^{-1}) 758, 807, 920, 1007, 1037, 1089, 1146, 1179, 1247, 1262, 1314, 1360, 1442, 1739, 2060, 2104, 2153, 2228, 2251, 2293, 2495, 2654, 2864, 2951, 3473.

Typical procedure II: synthesis of trifluoropyruvate derivatives [6]

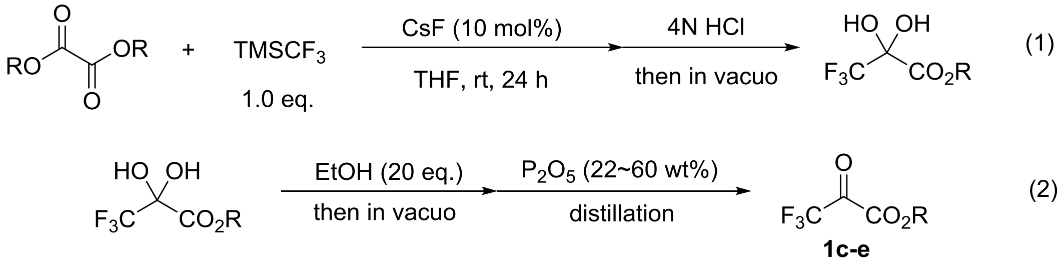

To an oven-dried 200-mL two-neck round-bottomed flask equipped with magnetic stir bar were added dialkyl oxalate (10 mmol), cesium fluoride (151.9 mg, 1 mmol), $TMSCF_3$ (1.48 mL, 10 mmol), and THF (60 mL). After the reaction mixture was stirred for 24 h at room temperature, 4 N HCl (30 mL) was added and concentrated in vacuo to give the corresponding trifluoropyruvate hydrate (reaction 1). Then, a solution of trifluoropyruvate hydrate in ethanol was stirred at 70 °C for 4 h, and evaporated. The hemiacetal pyruvate was purified by distillation with P_2O_5 to provide the corresponding trifluoropyruvate (**1c**,**d**) (reaction 2).

Isopropyl 3,3,3-trifluoro-2-oxopropanoate (1c)

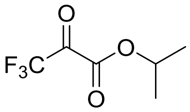

The compound was purified by distillation with P_2O_5 (55 wt %, 263 Torr, 35 °C) as a colorless liquid (25% yield).

^{1}H NMR (300 MHz, $CDCl_3$) δ 5.23 (sept, 1H, J = 6.3 Hz), 1.36 (d, 6H, J = 6.3 Hz); ^{13}C NMR (75 MHz, CDCl3) δ 174.4 (q, $J_{C\text{-}F}$ = 38.3 Hz), 156.3, 115.2 (q, $J_{C\text{-}F}$ = 288.4 Hz), 73.2, 21.4; ^{19}F NMR (282 MHz, $CDCl_3$) δ -75.7 (s, 3F); HRMS (APCI-TOF) calcd for $C_6H_7F_3O_3$ $[M]^{\cdot-}$: 184.0347, found: 184.0345; FT-IR (neat, cm^{-1}) 729, 810, 840, 904, 985, 1085, 1157, 1199, 1253, 1378, 1454, 1637, 1740, 2943, 2985, 3439.

Cyclopentyl 3,3,3-trifluoro-2-oxopropanoate (1d)

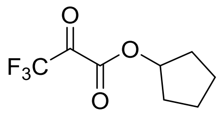

The compound was purified by distillation with P_2O_5 (60 wt%, 229 Torr, 29 °C) as a colorless liquid (25% yield).

^{1}H NMR (300 MHz, Acetone-d_6) δ 5.44-5.38 (m, 1H), 2.01-1.92 (m, 2H), 1.86-1.77 (m, 2H), 1.77-1.60 (m, 4H); ^{13}C NMR (75 MHz, Acetone-d_6) δ 174.0 (q, J_{C-F}= 36.4 Hz), 156.6, 116.3 (q, J_{C-F}= 288.2 Hz), 82.0, 33.0, 24.2; ^{19}F NMR (282 MHz, Acetone-d_6) δ 75.7 (s, 3F); HRMS (APCI-TOF) calcd for $C_8H_9F_3O_3$ $[M]^{-\cdot}$: 210.0538, found: 210.0530; FT-IR (neat, cm^{-1}) 728, 850, 950, 991, 1024, 1094, 1150, 1202, 1247, 1291, 1747, 1850, 2877, 2966, 3433.

Cyclohexyl 3,3,3-trifluoro-2-oxopropanoate (1e)

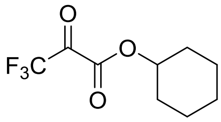

The compound was purified by distillation with P_2O_5 (42 wt%, 41 Torr, 52~53 °C) (containing small amount of cyclohexene) as a colorless liquid (42% yield).

^{1}H NMR (300 MHz, $CDCl_3$) δ 5.09-5.01 (m, 1H), 1.98-1.88 (m, 3H), 1.82-1.75 (m, 2H), 1.69-1.50 (m, 3H), 1.49-1.28 (m, 2H); ^{13}C NMR (75 MHz, $CDCl_3$) δ 174.6 (q, J_{C-F} = 38.5 Hz), 156.2, 115.2 (q, J_{C-F} = 288.4 Hz), 77.7, 31.1, 25.1, 23.4; ^{19}F NMR (282 MHz, $CDCl_3$) δ 75.7 (s, 3F); HRMS (APCI-TOF) calcd for $C_9H_{11}F_3O_3$ $[M]^{-\cdot}$: 224.0660, found: 224.0650; FT-IR (neat, cm^{-1}) 733, 828, 898, 1008, 1088, 1161, 1195, 1253, 1450, 1744, 1780, 2867, 2935, 3454.

2,4-Dimethylpentan-3-yl ethyl oxalate

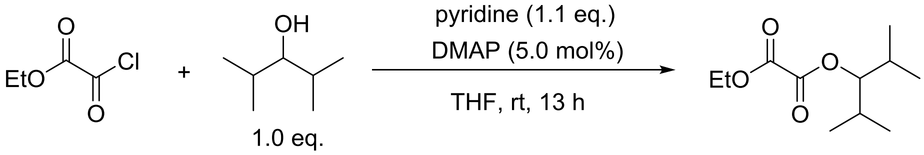

To an oven-dried 200-mL two-neck round-bottomed flask equipped with magnetic stir bar were added *N*,*N*'-dimethylaminopyridine (366 mg, 3.0 mmol), pyridine (5.3 mL, 66 mmol), 2,4-dimethylpentan-3-ol (8.4 mL, 60 mmol), and THF (200 mL). Ethyl chloroglyoxylate (3.3 mL, 60 mmol) was added dropwise to the solution. After stirring at room temperature for 13 h, the reaction mixture was diluted with H_2O (100 mL) and extracted with Et_2O (3 × 50 mL). The combined organic layers were washed with saturated aq. $NaHCO_3$ (100 mL), water (100 mL), brine (100 mL), and dried over $MgSO_4$, filtered and concentrated under reduced pressure. Resulting crude product was purified by silica-gel column chromatography (hexane:EtOAc 20:1) to give 2,4-dimethylpentan-3-yl ethyl oxalate as a clear liquid (6.4 g, >99%).

^{1}H NMR (300 MHz, $CDCl_3$) δ 4.73 (t, 1H, *J* = 6.2 Hz), 4.34 (q, 2H, *J* = 6.9 Hz), 2.00 (m, 2H), 1.37 (t, 3H, *J* = 7.0 Hz), 0.92 (d, 6H, *J* = 6.9 Hz), 0.90 (d, 6H, *J* = 6.6 Hz); ^{13}C NMR (75 MHz, $CDCl_3$) δ 158.6, 86.7, 63.0, 29.5, 19.5, 17.2, 14.1; HRMS (ESI-TOF) calcd for $C_{11}H_{20}NaO_4$ $[M+Na]^+$: 239.1259, found: 239.1270; FT-IR (neat, cm^{-1}) 760, 859, 894, 935, 1011, 1099, 1130, 1187, 1306, 1370, 1389, 1458, 1748, 1767, 2882, 2935, 2970.

2,4-Dimethylpentan-3-yl 3,3,3-trifluoro-2-oxopropanoate (1f)

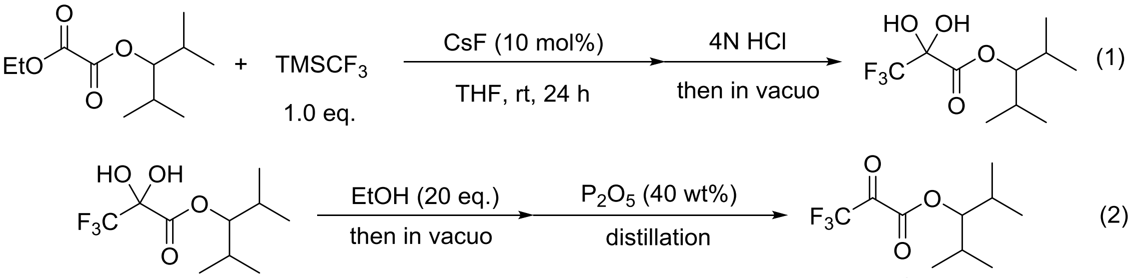

To an oven-dried 200-mL two-neck round-bottomed flask equipped with magnetic stir bar were added 2,4-dimethylpentan-3-yl ethyl oxalate (2.16 g, 10 mmol), cesium fluoride (151.9 mg, 1 mmol), $TMSCF_3$ (1.48 mL, 10 mmol), and THF (60 mL). After the reaction mixture was stirred for 24 h at room temperature, 4 N HCl (60 mL) was added and extracted with Et_2O (3 × 50 mL). The combined organic layers were washed with brine (100 mL), and dried over Na_2SO_4, filtered and concentrated under reduced pressure to afford the corresponding trifluoropyruvate hydrate as a yellow liquid (2.55 g, 99%) (reaction 1).

^{1}H NMR (300 MHz, $CDCl_3$) δ 4.76 (t, 1H, *J* = 6.1 Hz), 2.08-1.97 (m, 2H), 0.92 (d, 6H, *J* = 6.9 Hz), 0.91 (d, 6H, *J* = 6.6 Hz); ^{13}C NMR (75 MHz, $CDCl_3$) δ 174.0 (q, $J_{C\text{-}F}$ = 36.4 Hz), 156.7, 116.3 (q, $J_{C\text{-}F}$= 288.2 Hz), 82.0, 33.0, 24.2; ^{19}F NMR (282 MHz, $CDCl_3$) δ 82.7 (s, 3F); HRMS (APCI-TOF) calcd for $C_{10}H_{16}F_3O_4$ $[M-H]^-$: 243.0997, found: 243.0985; FT-IR (neat, cm^{-1}) 596, 657, 722, 878, 928, 985, 1085, 1165, 1191, 1253, 1366, 1386, 1466, 1366, 1386, 1466, 1740, 2878, 2943, 2974, 3454.

Then, a solution of trifluoropyruvate hydrate in ethanol was stirred at 70 °C for 4 h, and evaporated. The hemiacetal pyruvate was purified by distillation with P_2O_5 (40 wt%, 36 Torr, 52 °C) to provide the corresponding trifluoropyruvate (**1f**) as a colorless liquid (83% yield) (reaction 2).

^{1}H NMR (300 MHz, $CDCl_3$) δ 4.83 (t, 1H, *J* = 6.1 Hz), 2.12-1.97 (m, 2H), 0.93 (d, 6H, *J* = 6.9 Hz), 0.92 (d, 6H, *J* = 6.6 Hz); ^{13}C NMR (75 MHz, CDCl3) δ 174.7 (q, $J_{C\text{-}F}$ = 38.9 Hz), 157.0 (s), 115.0 (q, $J_{C\text{-}F}$ = 288.4 Hz), 88.5, 29.3, 19.2, 16.8; ^{19}F NMR (282 MHz, $CDCl_3$) δ 75.8 (s, 3F); HRMS (APCI-TOF) calcd for $C_{10}H_{15}F_3O_3$ $[M]^{-\cdot}$: 240.0973, found: 240.0973; FT-IR (neat, cm^{-1}) 720, 886, 931, 1010, 1089, 1172, 1191, 1254, 1304, 1371, 1393, 1469, 1743, 1777, 2879, 2943, 2977, 3459.

Typical procedure III: Cu-catalyzed asymmetric methylation of fluoroalkylated pyruvate

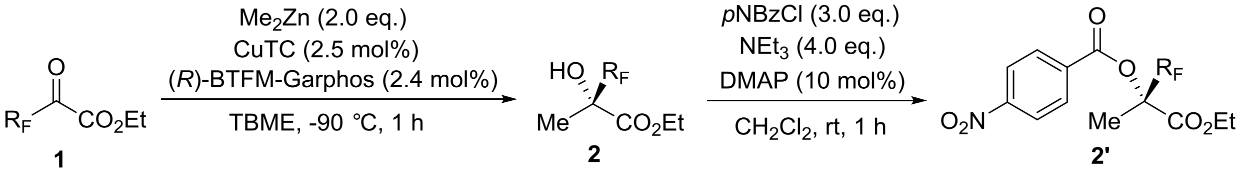

To a mixture of CuTC (1.0 mg, 0.005 mmol) and (*R*)-BTFM-Garphos (5.7 mg, 0.0048 mmol) was added CH_2Cl_2 (1.0 mL) at room temperature under argon atmosphere, and the solution was stirred for 12 h. The solvent was removed under reduced pressure, and the prepared catalyst was dissolved in TBME (0.5 mL) under argon atmosphere. After the solution was cooled to −90 °C, Me_2Zn (1.0 M in heptane, 0.4 mL, 0.4 mmol) followed by fluoroalkylated pyruvate **1** (0.2 mmol) in TBME (0.5 mL) were added in 30 min. The reaction mixture was stirred at the same temperature for 1 h. The reaction mixture was quenched with saturated aq. NH_4Cl. The organic layer was separated and the aqueous layer was extracted with Et_2O twice. The combined organic layer was dried over anhydrous Na_2SO_4 and evaporated under controlled pressure (350 mmHg). The concentrated solution was used without purification for the next protection reaction. The yield of alcohol product **2** was determined by ^{19}F NMR analysis using benzotrifluoride (BTF) as an internal standard.

To a solution of DMAP (2.4 mg, 0.02 mmol) and the crude alcohol in CH_2Cl_2 (2.0 mL) was added NEt_3 (56 μL, 0.4 mmol) at room temperature under argon atmosphere. After the reaction mixture was cooled to 0 °C, *p*-nitrobenzoyl chloride (56 mg, 0.3 mmol) was added. Then the mixture was warmed to room temperature and stirred for 1 h. After 1 N HCl (5.0 mL) was added to the reaction mixture, the organic layer was separated and the aqueous layer was extracted with Et_2O twice. The combined organic layer was washed with saturated aq. $NaHCO_3$, water, and brine, and then dried over anhydrous $MgSO_4$ and evaporated under reduced pressure. The residue was purified by silica gel column chromatography to give *p*-nitrobenzoylated alcohol **2’**. Enantiomeric excess was determined by chiral HPLC analysis.

(*S*)-3-Ethoxy-1,1,1-trifluoro-2-methyl-3-oxopropan-2-yl 4-nitrobenzoate (2a’)

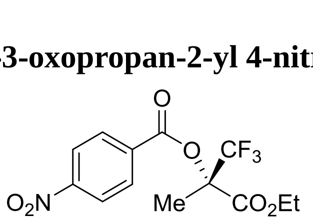

The yield of alcohol **2a** (86%) was determined by ^{19}F NMR analysis. *p*-Nitrobenzoylated alcohol **2a’** was purified by silica gel column chromatography (EtOAc/hexane 1:40) as a colorless liquid (53% yield for 2 steps, 89% ee).

^1H NMR (300 MHz, $CDCl_3$) δ 8.34-8.31 (m, 2H), 8.24-8.20 (m, 2H), 4.33 (q, 4H, J = 6.9 Hz), 1.97 (d, 3H, J = 0.9 Hz), 1.28 (t, 3H, J = 7.0 Hz); ^{13}C NMR (75 MHz, $CDCl_3$) δ 164.3, 162.3, 151.1, 134.0, 131.2, 123.7, 122.7 (q, $J_{C\text{-}F}$ = 282.9 Hz), 80.7 (q, $J_{C\text{-}F}$ = 30.4 Hz), 63.2, 16.6, 13.8; ^{19}F NMR (282 MHz, $CDCl_3$) δ -78.4 (s, 3F); HRMS (APCI-TOF) calcd for $C_{13}H_{12}F_3NO_6$ $[M]^{-\cdot}$: 335.0617, found: 335.0623; FT-IR (neat, cm^{-1}) 784, 813, 849, 876, 927, 1011, 1109, 1149, 1273, 1342, 1387, 1452,

1525, 1602, 1740, 1763, 2857, 2920, 2952, 2996, 3087, 3116; $[\alpha]_D^{22}$ -28.94 (*c* 0.20, $CHCl_3$), 89% *ee*; HPLC (column, CHIRALCEL OJ-3, Hexane/2-Propanol = 91/9, flow rate 0.6 mL/min, 20 °C detection UV 254 nm) t_R of major isomer 13.1 min, t_R of minor isomer 23.8 min.

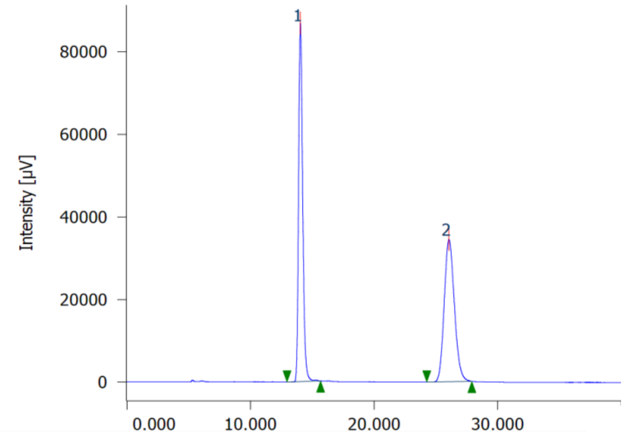

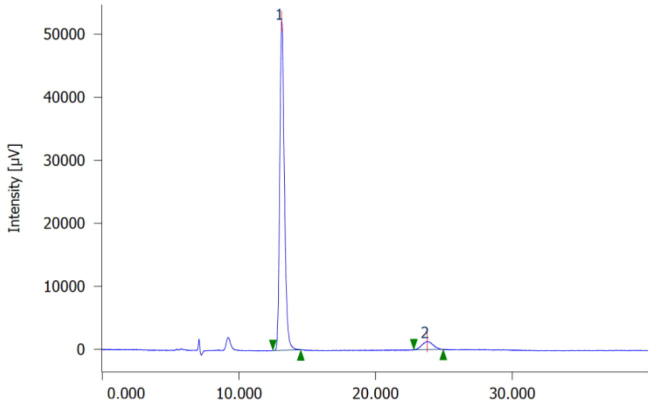

#	Time [min]	Area [µV·sec]	Area%	Height [µV]	Height%
1	14.0	1997861	50.35	86782	71.58
2	26.1	1970061	49.65	34456	28.42

#	Time [min]	Area [µV·sec]	Area%	Height [µV]	Height%
1	13.1	1238061	94.32	52128	97.56
2	23.8	74616	5.68	1305	2.44

(*S*)-1-Ethoxy-3,3-difluoro-2-methyl-1-oxopropan-2-yl benzoate (2g')

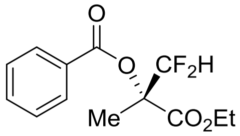

Reaction temperature was −78 °C. The yield of alcohol **2g** (89%) was determined by ^{19}F NMR analysis. In the protection of alcohol, benzoyl chloride was used instead of *p*-nitrobenzoyl chloride. Benzoylated alcohol **2g'** was purified by silica gel column chromatography (EtOAc/hexane = 1/40) as a colorless liquid (41% yield for 2 steps, 89% ee).

^{1}H NMR (300 MHz, $CDCl_3$) δ 8.05 (dd, *J* = 8.3, 1.3 Hz, 2H). 7.61 (tt, *J* = 6.7, 1.3 Hz, 1H), 7.44-7.49 (m, 2H), 6.30 (dd, $J_{H\text{-}F}$ = 56.8, 54.8 Hz, 1H), 4.29 (q, *J* = 7.1 Hz, 2H) 1.77 (t, $J_{H\text{-}F}$ = 1.6 Hz, 3H), 1.27 (t, *J* = 7.1 Hz, 3H); ^{13}C NMR (75 MHz, $CDCl_3$) δ 167.6, 164.8, 133.7, 130.0, 128.8, 128.5, 122.9 (dd, $J_{C\text{-}F}$ = 250.0, 245.0 Hz), 79.7 (dd, $J_{C\text{-}F}$ = 27.5, 21.9 Hz), 62.3, 14.6 (t, $J_{C\text{-}F}$ = 3.2 Hz) 13.9; ^{19}F NMR (282 MHz, $CDCl_3$) δ -128.40 (dd, *J* = 290.2 Hz, $J_{F\text{-}H}$ =54.7 Hz, 1F), -132.76 (dd, *J* = 289.90 Hz, $J_{F\text{-}H}$ =56.4 Hz, 1F); HRMS (APCI-TOF) calcd for $C_{13}H_{14}F_2NaO_4$ $[M+Na]^+$: 295.0758, found: 295.0761; FT-IR (neat, cm^{-1}) 1026, 1093, 1114, 1216, 1279, 1388, 1452, 1602, 1730, 1747, 2938, 2985, 3021; $[\alpha]_D^{25}$ -7.47 (*c* 1.01, $CHCl_3$), 89% *ee*; HPLC (column, CHIRALCEL OJ-3, Hexane/2-Propanol = 99/1, flow rate 0.6 mL/min, 20 °C detection UV 220 nm) t_R of major isomer 21.2 min, t_R of minor isomer 22.6 min.

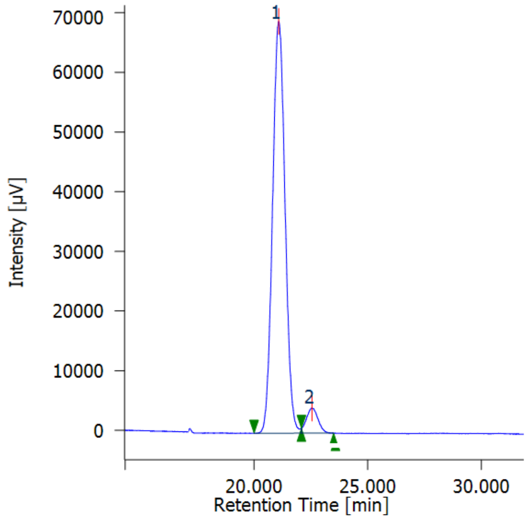

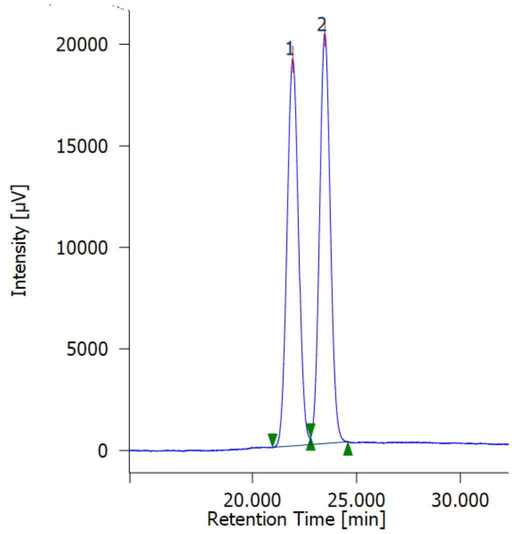

#	Time [min]	Area [µV·sec]	Area%	Height [µV]	Height%
1	21.9	730048	49.98	19047	48.53
2	23.5	730490	50.02	20201	51.47

#	Time [min]	Area [µV·sec]	Area%	Height [µV]	Height%
1	21.2	2632083	94.60	69000	94.24
2	22.6	150116	5.40	4219	5.76

(*S*)-1-Bromo-3-ethoxy-1,1-difluoro-2-methyl-3-oxopropan-2-yl 4-nitrobenzoate (2h’)

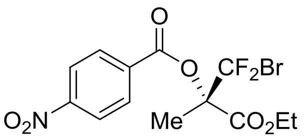

Reaction temperature was −78 °C. The yield of alcohol **2h** (53%) was determined by ^{19}F NMR analysis. *p*-Nitrobenzoylated alcohol **2h’** was purified by silica gel column chromatography (EtOAc/hexane 1:50) as a white solid (32% yield for 2 steps, 82% ee).

^{1}H NMR (300 MHz, $CDCl_3$) δ 8.34-8.31 (m, 2H), 8.25-8.21 (m, 2H), 4.32 (q, 2H, J = 7.2 Hz), 2.02 (s, 3H), 1.29 (t, 3H, J = 7.0 Hz); ^{13}C NMR (75 MHz, $CDCl_3$) δ 164.3, 162.4, 151.2, 134.3, 131.3, 123.9, 121.0 (t, $J_{C\text{-}F}$ = 311.6 Hz), 84.8 (dd, $J_{C\text{-}F}$ = 25.6, 23.4 Hz), 63.4, 18.4, 14.0; ^{19}F NMR (282 MHz, $CDCl_3$) δ -56.9 (d, 1F, J = 168.6 Hz), -58.9 (d, 1F, J = 165.3 Hz); HRMS (APCI-TOF) calcd for $C_{13}H_{12}BrF_2NO_6$ $[M]^{-\cdot}$: 394.9816, found: 394.9835; FT-IR (KBr pellet, cm^{-1}) 716, 843, 876, 961, 1020, 1106, 1146, 1280, 1347, 1446, 1528, 1610, 1751, 2866, 2936, 2988; $[\alpha]_D^{22}$ -11.99 (c 1.55, $CHCl_3$), 82% *ee*; HPLC (column, CHIRALCEL OD-3, Hexane/2-Propanol = 91/9, flow rate 0.6 mL/min, 20 °C detection UV 254 nm) t_R of major isomer 18.2 min, t_R of minor isomer 12.5 min.

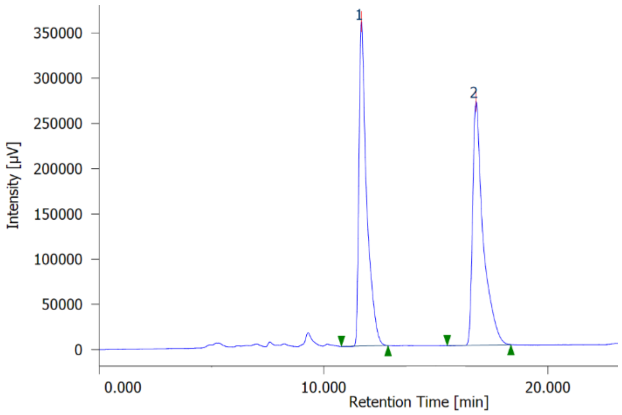

#	Time [min]	Area [µV·sec]	Area%	Height [µV]	Height%
1	11.7	8566119	49.44	358425	57.08
2	16.8	8760736	50.56	269527	42.92

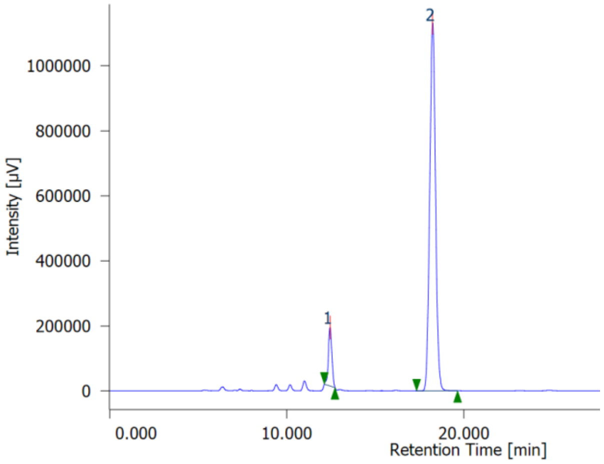

#	Time [min]	Area [µV·sec]	Area%	Height [µV]	Height%
1	12.5	2512608	8.83	180060	13.73
2	18.2	25954967	91.17	1131093	86.27

(*S*)-1-Ethoxy-3,3,4,4,4-pentafluoro-2-methyl-1-oxobutan-2-yl *p*-nitrobenzoate (2i')

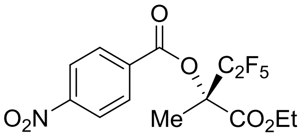

The yield of alcohol **2i** (87%) was determined by ^{19}F NMR analysis. *p*-Nitrobenzoylated alcohol **2i'** was purified by silica gel column chromatography (EtOAc/hexane 1:40) as a white solid (48% yield for 2 steps, 86% ee).

^{1}H NMR (300 MHz, $CDCl_3$) δ 8.30-8.35 (m, 2H) 8.16-8.21 (m, 2H), 4.27-4.37 (m, 2H), 2.04 (q, $J_{H\text{-}F}$ = 0.6 Hz, 3H), 1.28 (t, J = 7.1 Hz, 3H); ^{13}C NMR (75 MHz, $CDCl_3$) δ 164.2 162.2, (d, $J_{C\text{-}F}$ = 2.0 Hz) 151.0, 134.1, 131.0, 123.8, 118.6 (qt, $J_{C\text{-}F}$ = 286.1, 35.6 Hz), 112.0 (tq, $J_{C\text{-}F}$ = 263.0, 36.8 Hz), 81.3 (t, $J_{C\text{-}F}$ = 25.4 Hz), 63.3, 16.6, 13.7; ^{19}F NMR (282 MHz, $CDCl_3$) δ -79.19 (s, 3F), -121.42 (d, J = 280.9 Hz, 1F), -122.98 (d, J = 279.7 Hz, 1F); HRMS (APCI-TOF) calcd for $C_{14}H_{12}F_5NO_6$ $[M]^{\cdot-}$: 385.0585, found: 385.0582; FT-IR (KBr pellet, cm^{-1}) 1014, 1142, 1208, 1222, 1281, 1350, 1385, 1533, 1747, 2942, 2987, 3059; $[\alpha]_D^{25}$ -27.75 (*c* 1.02, $CHCl_3$), 86% *ee*; HPLC (column, CHIRALCEL OJ-3, Hexane/2-Propanol = 99/1, flow rate 0.6 mL/min, 20 °C detection UV 220 nm) t_R of major isomer 16.2 min, t_R of minor isomer 31.4 min.

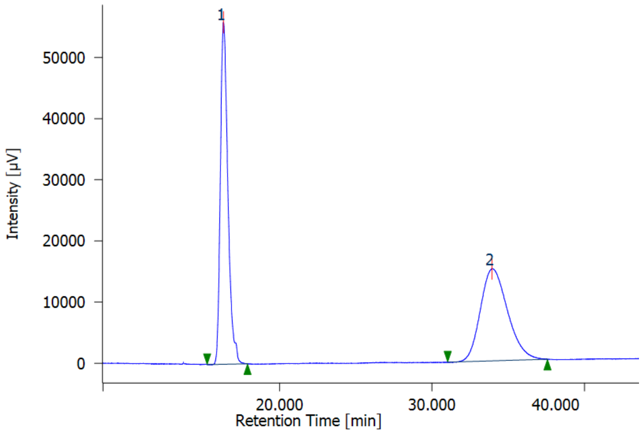

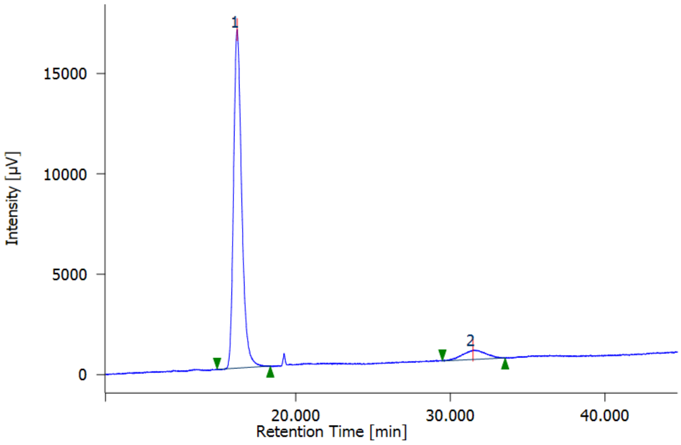

#	Time [min]	Area [µV·sec]	Area%	Height [µV]	Height%
1	16.3	1922723	50.80	55887	78.77
2	33.9	1862363	49.20	15067	21.23

#	Time [min]	Area [µV·sec]	Area%	Height [µV]	Height%
1	16.2	613879	92.76	16848	97.31
2	31.4	47932	7.24	466	2.69

(*S*)-1-Ethoxy-3,3,4,4,5,5,5-heptafluoro-2-methyl-1-oxopentan-2-yl *p*-nitrobenzoate (2j’)

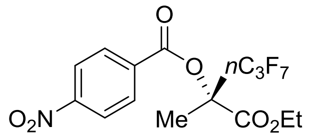

The yield of alcohol **2j** (98%) was determined by ^{19}F NMR analysis. *p*-Nitrobenzoylated alcohol **2j’** was purified by silica gel column chromatography (EtOAc/hexane 1:50) as a colorless oil (48% yield for 2 steps, 78% ee).

^{1}H NMR (300 MHz, $CDCl_3$) δ 8.31-8.35 (m, 2H) 8.16-8.21 (m, 2H), 4.29-4.36 (m, 2H), 2.07 (q, $J_{H\text{-}F}$ = 1.3 Hz, 3H), 1.28 (t, J = 7.1 Hz, 3H); ^{13}C NMR (75 MHz, $CDCl_3$) δ 164.2 162.2, 151.0, 134.1, 131.0, 123.8, 117.6 (qt, $J_{C\text{-}F}$ = 286.9, 33.9 Hz), 113.6 (tt, $J_{C\text{-}F}$ = 263.9, 30.9 Hz), 122.9 (tq, $J_{C\text{-}F}$ = 266.9, 37.4 Hz), 82.2 (t, $J_{C\text{-}F}$ = 25.7 Hz), 63.4, 16.8, 13.7; ^{19}F NMR (282 MHz, $CDCl_3$) δ -80.6--80.70 (m, 3F), -117.75 (d, J = 288.5 Hz, 1F), -119.60 (d, J = 288.2 Hz, 1F), -123.882 (s, 2F).

HRMS (APCI-TOF) calcd for $C_{15}H_{12}F_7NO_6$ $[M]^{\cdot-}$: 435.0553, found: 435.0547; FT-IR (neat, cm^{-1}) 1090, 1140, 1200, 1233, 1349, 1387, 1534, 1609, 1744, 1761, 2942, 2988, 3059; $[\alpha]_D^{25}$ -22.60 (*c* 0.94, $CHCl_3$), 78% *ee*; HPLC (column, CHIRALCEL OJ-3, Hexane/2-Propanol = 99/1, flow rate 0.6 mL/min, 20 °C detection UV 220 nm) t_R of major isomer 12.2 min, t_R of minor isomer 15.5 min.

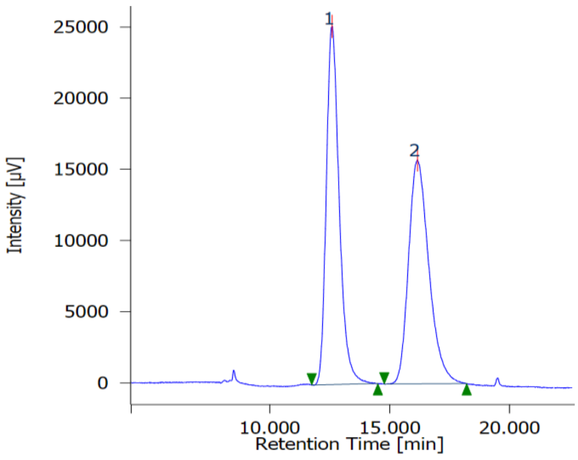

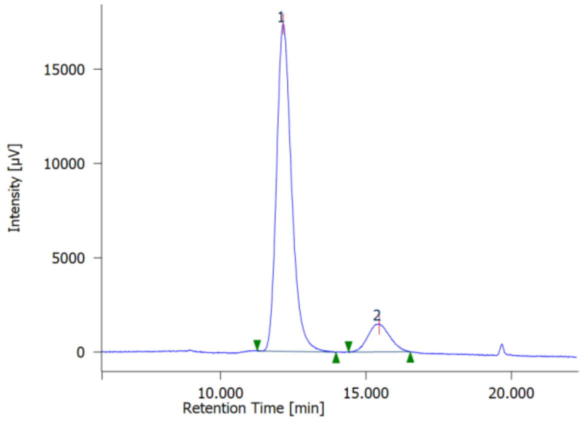

#	Time [min]	Area [µV·sec]	Area%	Height [µV]	Height%
1	12.7	942415	50.43	25143	61.52
2	16.2	926323	49.57	15729	38.48

#	Time [min]	Area [µV·sec]	Area%	Height [µV]	Height%
1	12.2	619959	88.86	17358	92.11
2	15.5	77697	11.14	1486	7.89

(*S*)-1-Ethoxy-3,3,4,4,5,5,6,6,7,7,8,8,9,9,10,10,10-heptadecafluoro-2-methyl-1-oxodecan-2-yl *p*-nitrobenzoate (2k’)

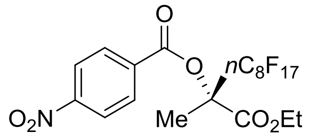

The yield of alcohol **2k** (92%) was determined by ^{19}F NMR analysis. *p*-Nitrobenzoylated alcohol **2k’** was purified by silica-gel column chromatography (EtOAc/hexane 1:50) as a white solid (85% yield

for 2 steps, 73% ee).

^{1}H NMR (300 MHz, $CDCl_3$) δ 8.36-8.31 (m, 2H), 8.20-8.16 (m, 2H), 4.36-4.29 (m, 2H), 2.08 (s, 3H), 1.28 (t, 3H, J = 7.3 Hz); ^{13}C NMR (75 MHz, $CDCl_3$) δ 164.3 162.4 151.2 134.3 131.2 123.9 118.4-104.7 (m) 117.3 (qt, J_{C-F} = 288.4, 33.2 Hz), 82.8 (t, J_{C-F} = 25.4 Hz), 63.5, 17.1,13.8; ^{19}F NMR (282 MHz, $CDCl_3$) δ -80.6--80.8 (m, 3F), -116.4--126.0 (m, 14F); HRMS (APCI-TOF) calcd for $C_{20}H_{12}F_{17}NO_6$ $[M]^{-\cdot}$: 685.0393, found: 685.0362; FT-IR (KBr pellet cm^{-1}) 847, 969, 1009, 1142, 1214, 1246, 1297, 1472, 1530, 1613, 1732, 1757, 2339, 2360, 2860, 2922, 2997, 3112, 3454, 3493; $[\alpha]_D^{22}$ -11.93 (c 0.48, $CHCl_3$), 73% *ee*; HPLC (column, CHIRALPAK AD-3 and AD-H, Hexane/2-Propanol = 99.5/0.5, flow rate 0.6 mL/min, 20 °C detection UV 254 nm) t_R of major isomer 16.8 min, t_R of minor isomer 24.4 min.

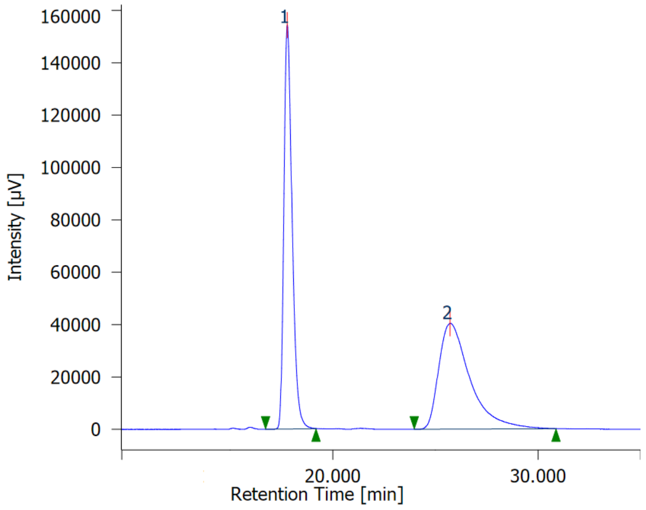

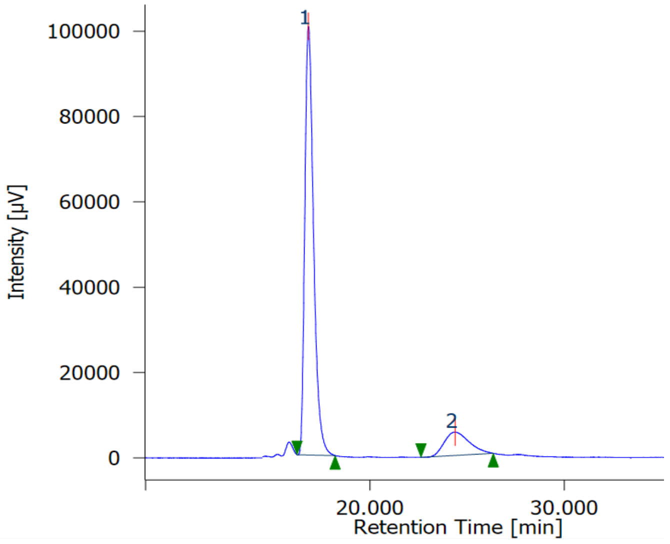

#	Time [min]	Area [µV·sec]	Area%	Height [µV]	Height%
1	17.8	4310404	50.22	154197	79.25
2	25.7	4271875	49.78	40381	20.75

#	Time [min]	Area [µV·sec]	Area%	Height [µV]	Height%
1	16.8	3133218	86.56	100378	94.81
2	24.4	486644	13.44	5495	5.19

Typical procedure IV: Cu-catalyzed asymmetric methylation of trifluoropyruvate derivatives

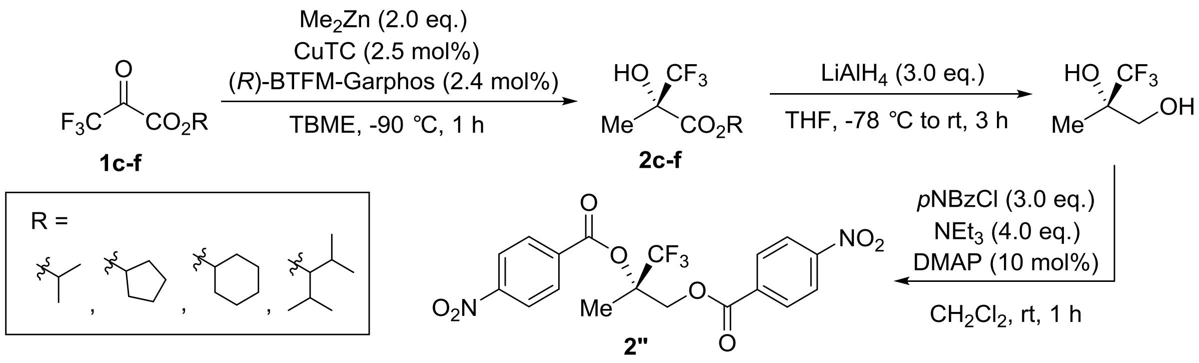

To a mixture of CuTC (1.0 mg, 0.005 mmol) and (*R*)-BTFM-Garphos (5.7 mg, 0.0048 mmol) was added CH_2Cl_2 (1.0 mL) at room temperature under argon atmosphere, and the solution was stirred for 12 h. The solvent was removed under reduced pressure, and the prepared catalyst was dissolved in TBME (0.5 mL). After the solution was cooled to −90 °C, Me_2Zn (1.0 M in heptane, 0.4 mL, 0.4 mmol), followed by trifluoropyruvate derivative **1** (0.2 mmol) in TBME (0.5 mL) were added in 30 min. The reaction mixture was stirred at the same temperature for 1 h. After the reaction mixture was quenched with saturated aq. NH_4Cl, the organic layer was separated and the aqueous layer was extracted with Et_2O twice. The combined organic layer was dried over anhydrous Na_2SO_4 and evaporated under controlled pressure (350 mmHg). The yields (**2c**: 59%, **2d**: 60%, **2e**: 64%, **2f**: 71%) were determined by ^{19}F NMR analysis using BTF as an internal standard. The concentrated solution was used without purification for the next reduction with $LiAlH_4$.

To an oven-dried 30-mL two-neck round-bottomed flask equipped with magnetic stir bar was added $LiAlH_4$ (23 mg, 0.6 mmol) in THF (10 mL). After the solution was cooled to −78 °C, the crude alcohol in THF (1 mL) was added dropwise. Then the mixture was warmed to room temperature and stirred for 3 h. The reaction mixture was quenched with water (23 μL), 15% NaOH aq. (23 μL), and water (69 μL). After filtration, the solvent was evaporated under reduced pressure. The concentrated solution was used without purification for the next protection reaction.

To a solution of DMAP (2.4 mg, 0.02 mmol) and the crude diol in CH_2Cl_2 (2.0 mL) was added NEt_3 (112 μL, 0.8 mmol) at room temperature. After the reaction mixture was cooled to 0 °C, *p*-nitrobenzoyl chloride (112 mg, 0.6 mmol) was added. The mixture was warmed to room temperature and stirred for 1 h and 1 N HCl (5.0 mL) was added to the reaction mixture. The organic layer was separated and the aqueous layer was extracted with Et_2O twice. The combined organic layers were washed with saturated aq. $NaHCO_3$, water, brine, and dried over anhydrous $MgSO_4$ and evaporated under reduced pressure. The residue was purified by silica-gel column chromatography (EtOAc/hexane 1:20) to give the corresponding di-*p*-nitrobenzoylated product **2”** as a white solid (14% yield for 3 steps, 94% ee). Enantiomeric excess was determined by chiral HPLC analysis.

^1H NMR (300 MHz, $CDCl_3$) δ 8.31 (d, 2H, *J* = 9.0 Hz), 8.25 (d, 2H, *J* = 8.7 Hz), 8.18 (d, 2H, *J* = 9.0 Hz), 8.12 (d, 2H, *J* = 8.7 Hz), 5.25 (dd, 1H, *J* = 12.6, 0.9 Hz), 4.88 (d, 1H, *J* = 12.9 Hz), 1.95 (s, 3H); ^{13}C NMR (75 MHz, $CDCl_3$) δ 163.7, 162.5, 151.2, 151.0, 134.8, 134.5, 131.1, 130.9, 124.1 (q, $J_{C\text{-}F}$ =

282.5 Hz), 123.9, 123.8, 81.4 (q, J_{C-F} = 29.1 Hz), 63.0, 16.4 (q, J_{C-F} = 1.3 Hz); ^{19}F NMR (282 MHz, $CDCl_3$) δ 79.7 (s, 3F); HRMS (APCI-TOF) calcd for $C_{18}H_{13}F_3N_2O_8$ [M]$^{-\bullet}$: 442.0624, found: 442.0603; FT-IR (KBr pellet, cm^{-1}) 822, 1102, 1119, 1171, 1271, 1342, 1523, 1734, 1748, 2337, 2362, 2588, 2923, 2958, 3056, 3081, 3113; $[\alpha]_D^{23}$ +23.80 (*c* 0.06, $CHCl_3$), 94% *ee*; HPLC (column, CHIRALCEL OD-3, Hexane/2-Propanol = 91/9, flow rate 0.6 mL/min, 20 °C detection UV 254 nm) t_R of major isomer 47.8 min, t_R of minor isomer 40.7 min.

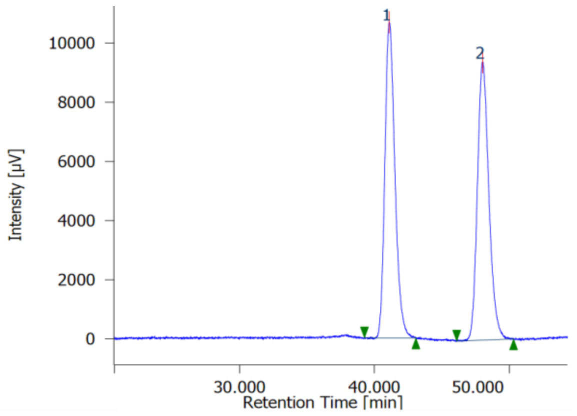

#	Time [min]	Area [µV·sec]	Area%	Height [µV]	Height%
1	41.1	553535	49.83	10673	53.20
2	48.0	557375	50.17	9389	46.80

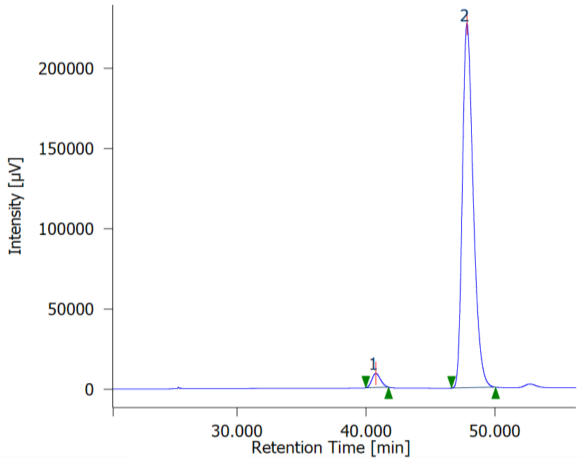

#	Time [min]	Area [µV·sec]	Area%	Height [µV]	Height%
1	40.7	412890	3.01	8868	3.76
2	47.8	13310124	96.99	226983	96.24

Determination of the absolute configuration (Table 1, entry 17)

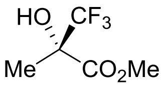

To a mixture of CuTC (9.5 mg, 0.05 mmol) and (*R*)-DTB-MeO-BIPHEP (55.7 mg, 0.054 mmol) was added was added CH_2Cl_2 (5.0 mL) at room temperature under argon atmosphere, and the solution was stirred for 12 h. The solvent was removed under reduced pressure, and the prepared catalyst was dissolved in TBME (5.0 mL) under an argon atmosphere. After the solution was cooled to −78 °C, Me_2Zn (1.0 M in heptane, 4.0 mL, 4.0 mmol) followed by methyl trifluoropyruvate **1b** (244 µL, 2.0 mmol) in TBME (5.0 mL) were added. The reaction mixture was stirred at the same temperature for 1 h. The reaction mixture was quenched with saturated aq. NH_4Cl. The organic layer was separated and the aqueous layer was extracted with Et_2O twice. The combined organic layer was dried over anhydrous Na_2SO_4 and evaporated under controlled pressure (350 mmHg), and the residue was purified by a distillation (70 kPa, 20 °C) to give **2b** in 8% yield. The product **2b** exhibited the same ^{1}H and ^{19}F NMR spectra as reported before [7]. ^{1}H NMR (300 MHz, $CDCl_3$) δ 1.59 (s, 3H), 3.77 (s, 1H), 3.91 (s, 3H); ^{19}F NMR (282 MHz, $CDCl_3$) δ −80.04 (s, 3F); $[\alpha]_D^{25}$ −2.12 (*c* 2.20, $CHCl_3$).

The absolute configuration was determined to be *S* in comparison with the optical rotation of reported data [Ref 7: (*S*)-**2b** >95% ee, $[\alpha]_D^{20}$ = −14.4 (*c* 3.65, $CHCl_3$)]. The absolute configurations of other alcohol products **2a** and **2c–k** were tentatively assigned by analogy to **2b**. Enantiomeric excess of

benzoylated alcohol **2b'** prepared by **Typical Procedure III** was determined by chiral HPLC analysis. Compound **2b'** was purified by silica-gel column chromatography (EtOAc/hexane 1:40) as a colorless oil (60% yield for 2 steps, 59% ee).

^{1}H NMR (300 MHz, $CDCl_3$) δ 1.94 (q, $J_{H\text{-}F}$ = 1.0 Hz, 3H), 3.84 (s, 3H), 7.45-7.50 (m, 2H), 7.63 (tt, J = 7.4, 1.9 Hz, 1H), 8.03-8.07 (m, 2H); ^{13}C NMR (75 MHz, $CDCl_3$) δ 16.7 (d, $J_{C\text{-}F}$ = 1.2 Hz), 53.6, 79.8 (q, $J_{C\text{-}F}$ = 30.2 Hz), 122.9 (q, $J_{C\text{-}F}$ = 282.8 Hz), 128.6, 129.7, 130.1, 134.0, 164.2, 165.6; ^{19}F NMR (282 MHz, $CDCl_3$) δ -78.45 (s, 3F); HRMS (APCI-TOF) calcd for $C_{12}H_{11}F_3NaO_4$ $[M+Na]^+$: 299.0507, found: 299.0493; FT-IR (neat, cm^{-1}) 1069, 1110, 1137, 1188, 1216, 1271, 1289, 1304, 1384, 1452, 1602, 1734, 1759, 2928, 2958, 3022; $[\alpha]_D^{25}$ +21.07 (*c* 0.80, $CHCl_3$).

Synthesis of simple perfluoroalkylated ketone (3a) [8]

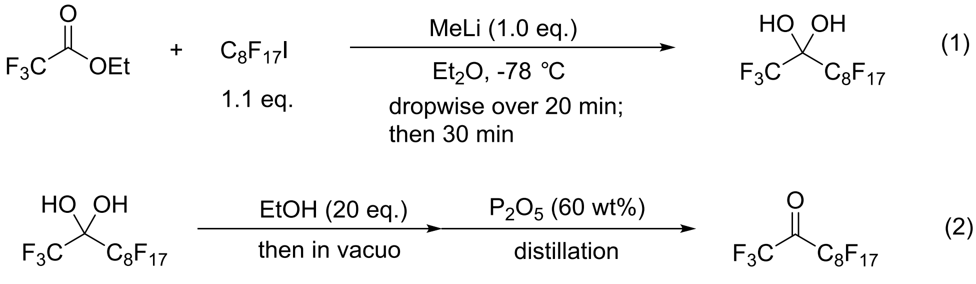

3a

To an oven-dried 200-mL two-neck round-bottomed flask equipped with magnetic stir bar were added ethyl trifluoroacetate (60 mmol), $C_8F_{17}I$ (66 mmol), and Et_2O (200 mL) under argon atmosphere. After the solution was cooled to -78 °C, MeLi (60 mmol) was added dropwise in 20 min. After stirring at −78 °C for 30 min, the reaction mixture was diluted with 1N HCl (100 mL) and extracted with Et_2O (3 × 50 mL). The combined organic extracts were washed with saturated aq. $NaHCO_3$ (100 mL), brine (100 mL), dried over Na_2SO_4, filtered and concentrated under reduced pressure. The hydrate product is known compound, the following data are identical to those given in corresponding literature [8] and used for next step without further purification. The yield was determined by ^{19}F NMR analysis using BTF as an internal standard (reaction 1).

Pink liquid (96% yield). ^{19}F NMR (282 MHz, $CDCl_3$) δ -81.2 (brs, 3F), -81.6 (brs, 3F) -120.4 (brs, 2F), -121.5--122.0 (m, 8F), -122.8 (brs, 2F), -126.2 (brs, 2F).

A solution of hydrate (10 mmol) in ethanol (200 mmol) was stirred at 70 °C for 4 h, and evaporated under reduced pressure. The ethyl acetal was purified by distillation with P_2O_5 (760 Torr, 36 °C) to provide the corresponding perfluoroalkylated ketone **3a**.

Colorless liquid (39% yield); ^{13}C NMR (75 MHz, Acetone-d_6) δ 176.0-173.5 (m), 114.3 (qt, $J_{C\text{-}F}$ = 287.9 Hz), 114.3 (q, $J_{C\text{-}F}$ = 287.9 Hz), 114.2-104.5 (m); ^{19}F NMR (282 MHz, Acetone-d_6) δ -75.2 (s, 3F), -81.8-81.9 (m, 3F), -118.9 (brs, 2F), -122.0--122.5 (m, 8F), -123.2 (brs, 2F), -126.9 (brs, 2F); HRMS (APCI-TOF) calcd for $C_{10}F_{20}O$ $[M]^{\cdot-}$: 515.9630, found: 515.9628; FT-IR (neat, cm^{-1}) 527, 558, 655, 724, 787, 818, 852, 910, 1004, 1062, 1146, 1208, 1793.

Catalytic asymmetric methylation of perfluoroalkylated ketone

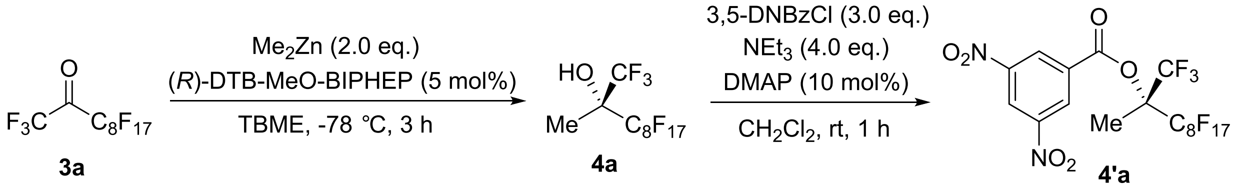

To a solution of (*R*)-DTB-MeO-BIPHEP (5.6 mg, 0.005 mmol) and **3a** (30 μL, 0.1 mmol) in TBME (1.0 mL) was added Me_2Zn (1.0 M in heptane, 0.2 mL, 0.2 mmol) dropwise at −78 °C under argon atmosphere, and then the solution was stirred for 3 h. The reaction mixture was quenched with saturated aq. NH_4Cl. The organic layer was separated and the aqueous layer was extracted with Et_2O twice. The combined organic layer was dried over anhydrous Na_2SO_4 and evaporated under controlled pressure (200 mmHg). The concentrated solution was used without purification for the next protection reaction. The yield of alcohol product **4a** (87%) was determined by ^{19}F NMR analysis using benzotrifluoride (BTF) as an internal standard.

^1H NMR (300 MHz, $CDCl_3$) δ 2.90 (s, 1H), 1.67 (s, 3H); ^{19}F NMR (282 MHz, CDCl3) δ -78.1 (s, 3F), -82.0--81.0 (m, 3F), -117.8--120.3 (m, 4F), -121.6--122.0 (m, 6F), -122.9 (s, 2F), -126.3 (brs, 2F).

To a solution of DMAP (1.2 mg, 0.01 mmol) and the crude product in CH_2Cl_2 (2.0 mL) was added NEt_3 (56 μL, 0.4 mmol) at room temperature under argon atmosphere. After the reaction mixture was cooled to 0 °C, 3,5-dinitrobenzoyl chloride (69 mg, 0.3 mmol) was added. Then the mixture was warmed to room temperature and stirred for 1 h. After 1 N HCl (5.0 mL) was added to the reaction mixture. The organic layer was separated and the aqueous layer was extracted with Et_2O twice. The combined organic layer was washed with saturated aq. $NaHCO_3$, water, brine, and dried over anhydrous $MgSO_4$, and evaporated under reduced pressure. The residue was purified by silica-gel column chromatography (EtOAc/hexane 1:60) to give 3,5-dinitrobenzoylated alcohol **4'a** as a white solid (45% yield for 2 steps, 24% ee). Enantiomeric excess was determined by chiral HPLC analysis. The absolute configuration was not determined.

^1H NMR (300 MHz, $CDCl_3$) δ 9.30 (t, 1H, J = 2.1 Hz), 9.11 (d, 2H, J = 2.1 Hz), 2.28 (s, 3H); ^{13}C NMR (75 MHz, $CDCl_3$) δ 158.6, 148.9, 132.3, 129.7, 123.4, 121.7 (q, $J_{C\text{-}F}$ = 286.5 Hz), 120.5-104.3 (m), 117.1 (q, $J_{C\text{-}F}$ = 286.8, 32.1 Hz), 84.9-83.9 (m); ^{19}F NMR (282 MHz, $CDCl_3$) δ -73.5 (m, 3F), -80.8 (m, 3F), -116.4 (brs, 2F), -119.2 (brs, 2F), -121.4--121.9 (m, 6F), -122.7 (brs, 2F), -126.1 (brs, 2F): HRMS (ESI-TOF) calcd for $C_{13}H_{13}F_3NaO_4$ $[M]^{-}$: 725.9907, found: 725.9934; FT-IR (KBr pellet, cm^{-1}) 653, 732, 928, 969, 1100, 1149, 1217, 1254, 1341, 1458, 1544, 1634, 1763, 2861, 2925, 2954, 3105; $[\alpha]_D^{22}$ -1.76 (*c* 0.61, $CHCl_3$), 24% *ee*; HPLC (column, CHIRALCEL OD-3, Hexane/2-Propanol = 96/4, flow rate 0.8 mL/min, 20 °C detection UV 254 nm) t_R of major isomer 19.1 min, t_R of minor isomer 22.4 min.

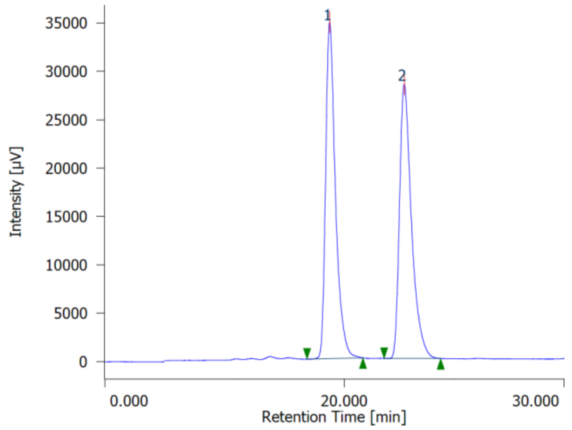

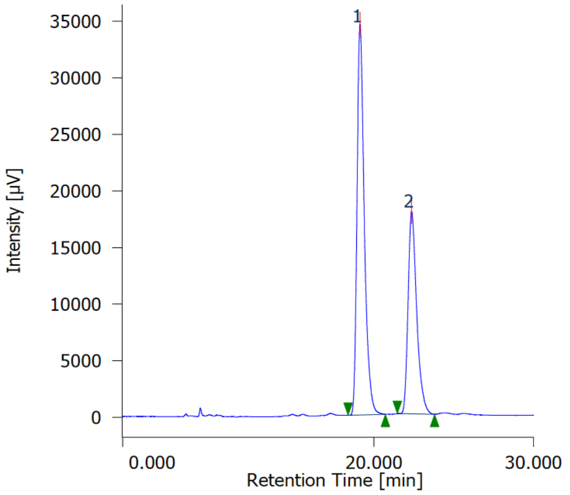

#	Time [min]	Area [µV·sec]	Area%	Height [µV]	Height%
1	19.3	1077441	50.16	34774	55.11
2	22.7	1070546	49.84	28327	44.89

#	Time [min]	Area [µV·sec]	Area%	Height [µV]	Height%
1	19.1	1092999	61.99	34497	65.87
2	22.4	670234	38.01	17877	34.13

References

[1] Aikawa, K.; Yoshida, S.; Kondo, D.; Asai, Y.; Mikami, K. *Org. Lett.* **2015**, *17*, 5108.

[2] Osipov, S. N.; Golubev, A. S.; Sewald, N.; Michel, T.; Kolomiets, A. F.; Fokin, A. V.; Burger, K. *J. Org. Chem.* **1996**, *61*, 7521.

[3] Chen, L. S.; Chen, G. J.; Tamborski, C. *J. Fluorine Chem.* **1984**, *26*, 341.

[4] Kropp, P. J.; Daus, K. A.; Tubergen, M. W.; Kepler, K. D.; Wilson, V. P.; Craig, S. L.; Baillargeon, M. M.; Breton. G. W. *J. Am. Chem. Soc.* **1993**, *115*. 3071.

[5] Denton, R. M.; An, J.; Adeniran, B.; Blake, A. J.; Lewis, W.; Poulton, A. M. *J. Org. Chem.* **2011**, *76*, 6749.

[6] Chen, L. S.; Chen, G. J. *J. Fluorine Chem.* **1989**, *42*, 371.

[7] Konigsberger, K.; Prasad, K.; Repič, O. *Tetrahedron: Asymmetry* **1999**, *10*, 679.

[8] Legros, J.; Crousse, B; Delpon, D. B.; Bégué, J. P. *Tetrahedron* **2002**, *58*, 3993.

^1H NMR

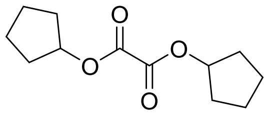

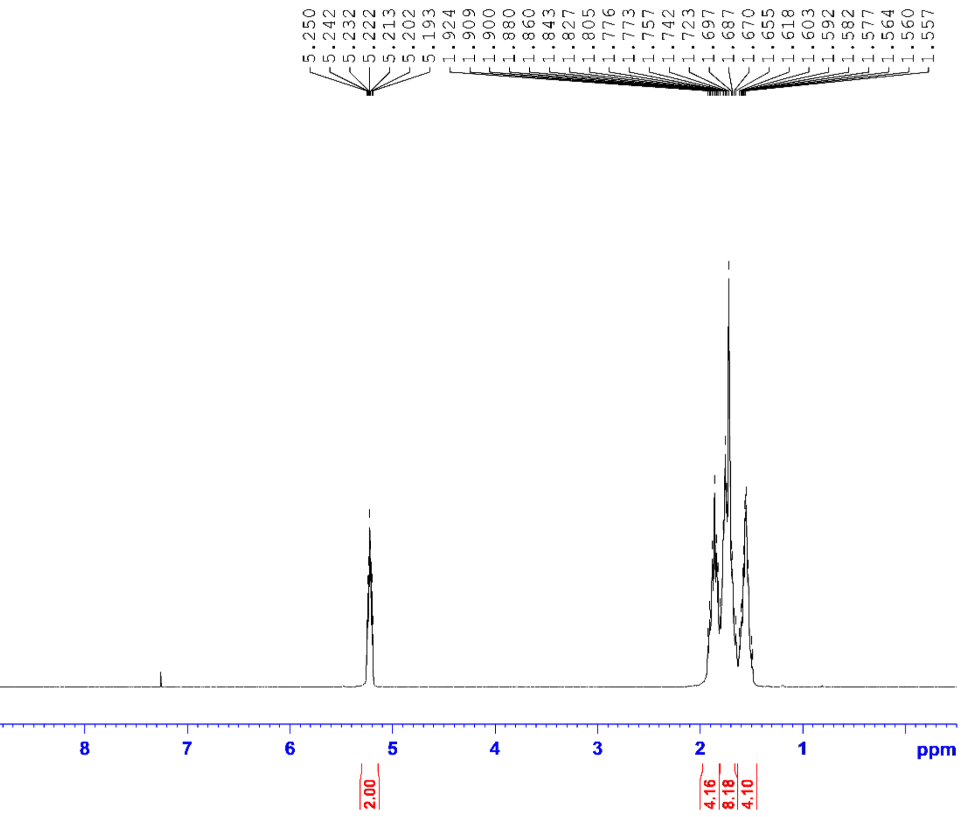

^{13}C NMR

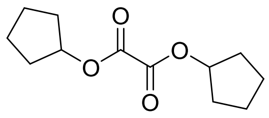

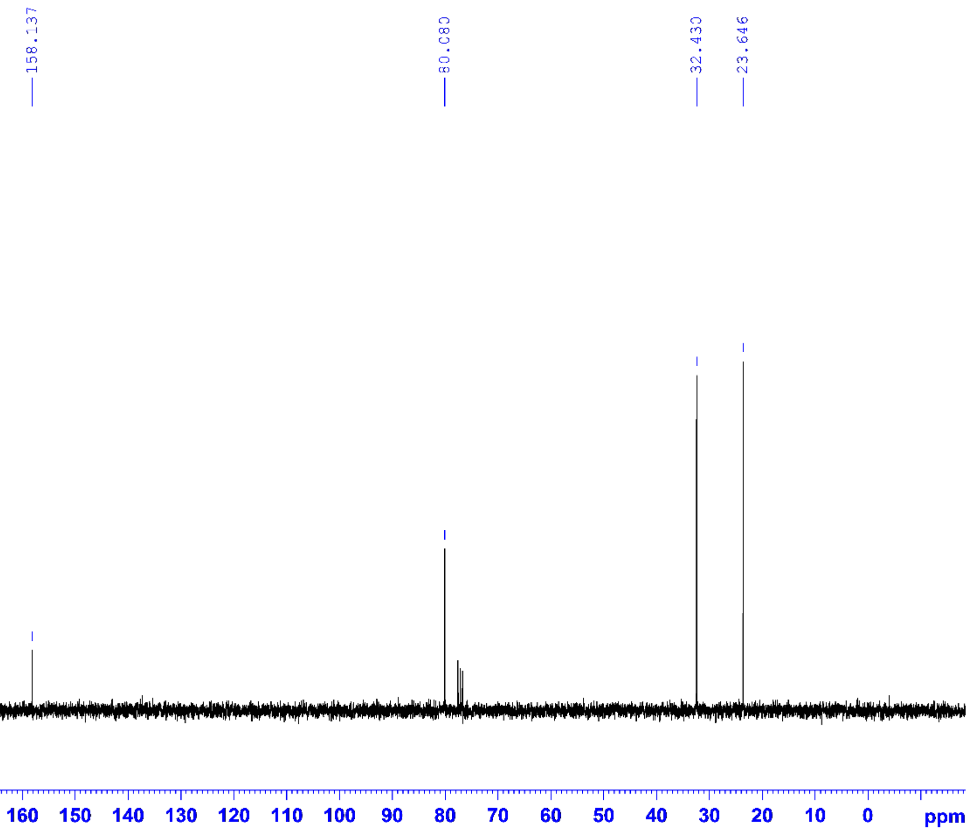

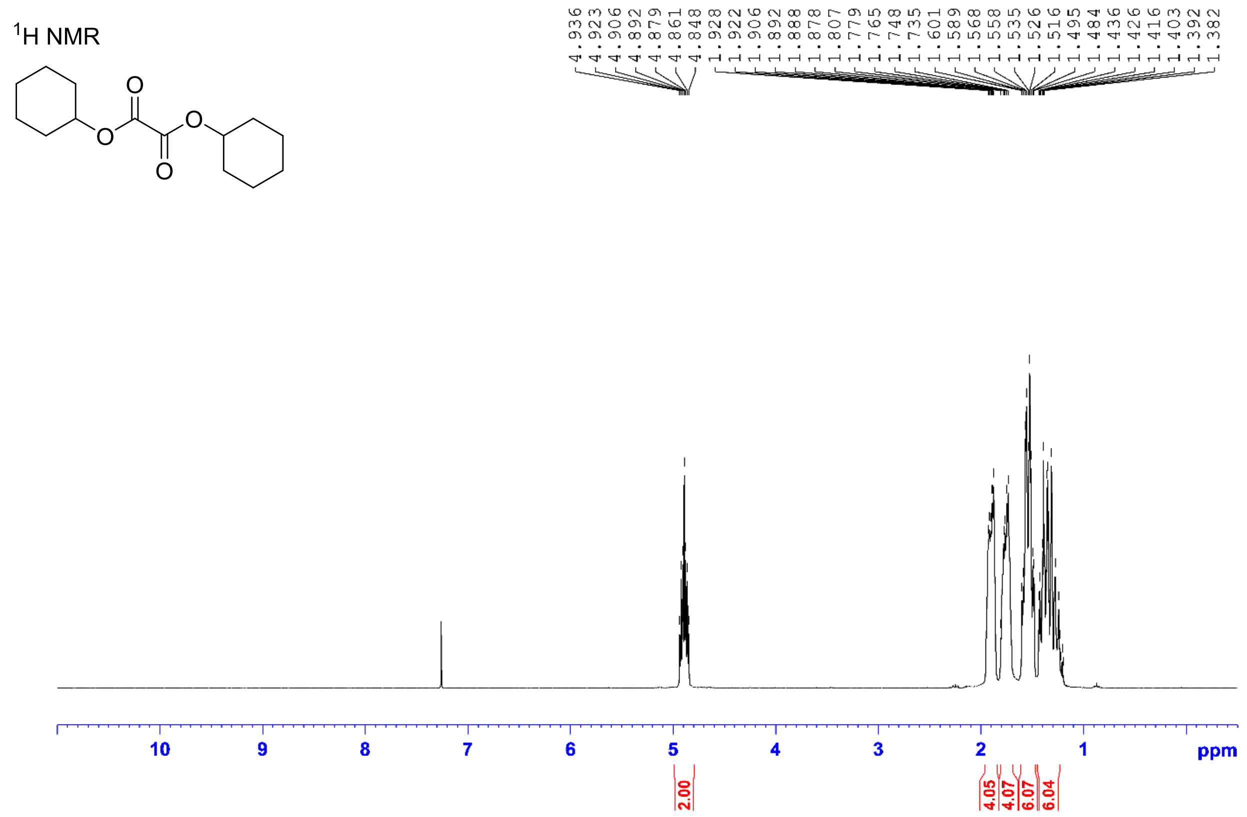
1H NMR
O
O
O
O
4.936
4.923
4.906
4.892
4.879
4.861
4.848
1.928
1.922
1.906
1.892
1.888
1.878
1.807
1.779
1.765
1.748
1.735
1.601
1.589
1.568
1.558
1.535
1.526
1.516
1.495
1.484
1.436
1.426
1.416
1.403
1.392
1.382
10
9
8
7
6
5
4
3
2
1
ppm
2.00
4.05
4.07
6.07
6.04

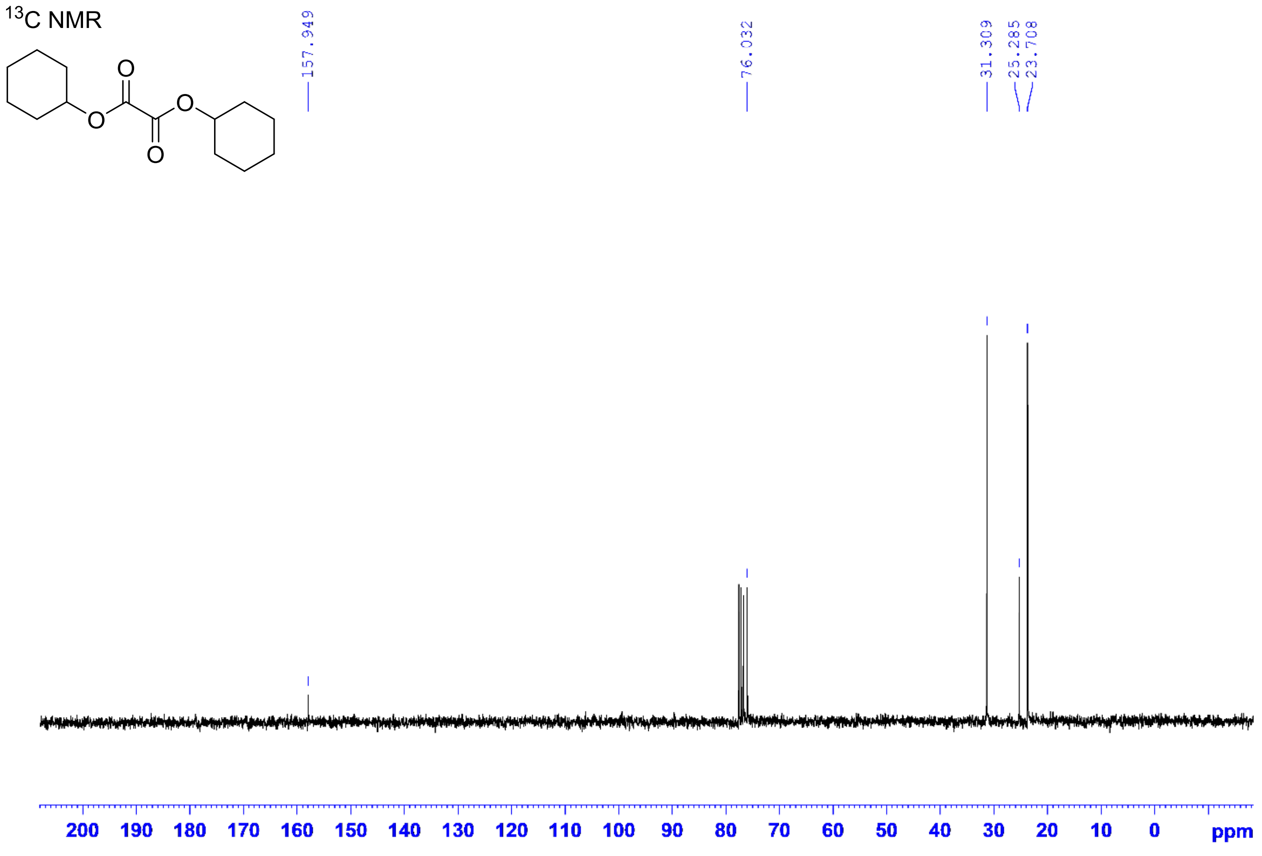
13C NMR
O
O
O
O
157.949
76.032
31.309
25.285
23.708
200
190
180
170
160
150
140
130
120
110
100
90
80
70
60
50
40
30
20
10
0
ppm

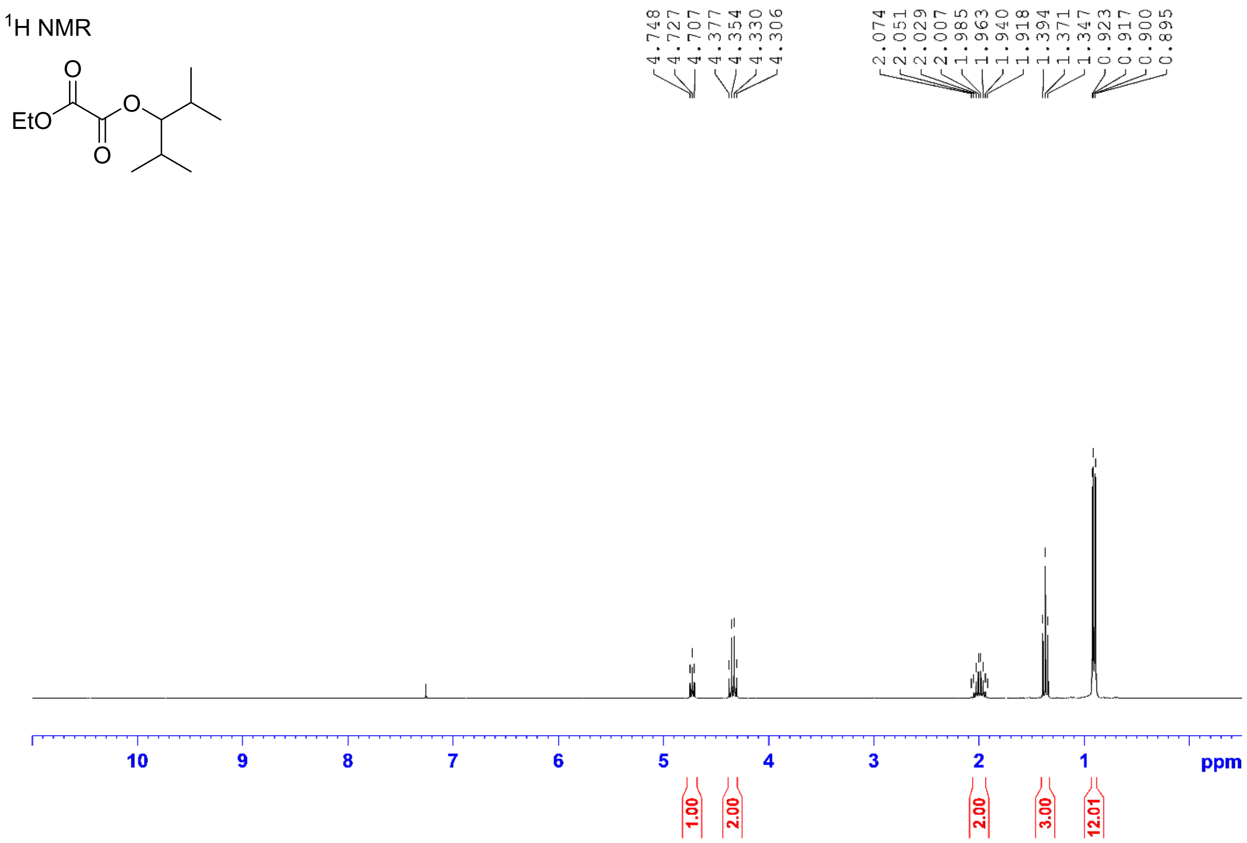

^{1}H NMR
EtO
O
4.748 4.727 4.707 4.377 4.354 4.330 4.306
2.074 2.051 2.029 2.007 1.985 1.963 1.940 1.918 1.394 1.371 1.347 0.923 0.917 0.900 0.895
10 9 8 7 6 5 4 3 2 1 ppm
1.00 2.00 2.00 3.00 12.01

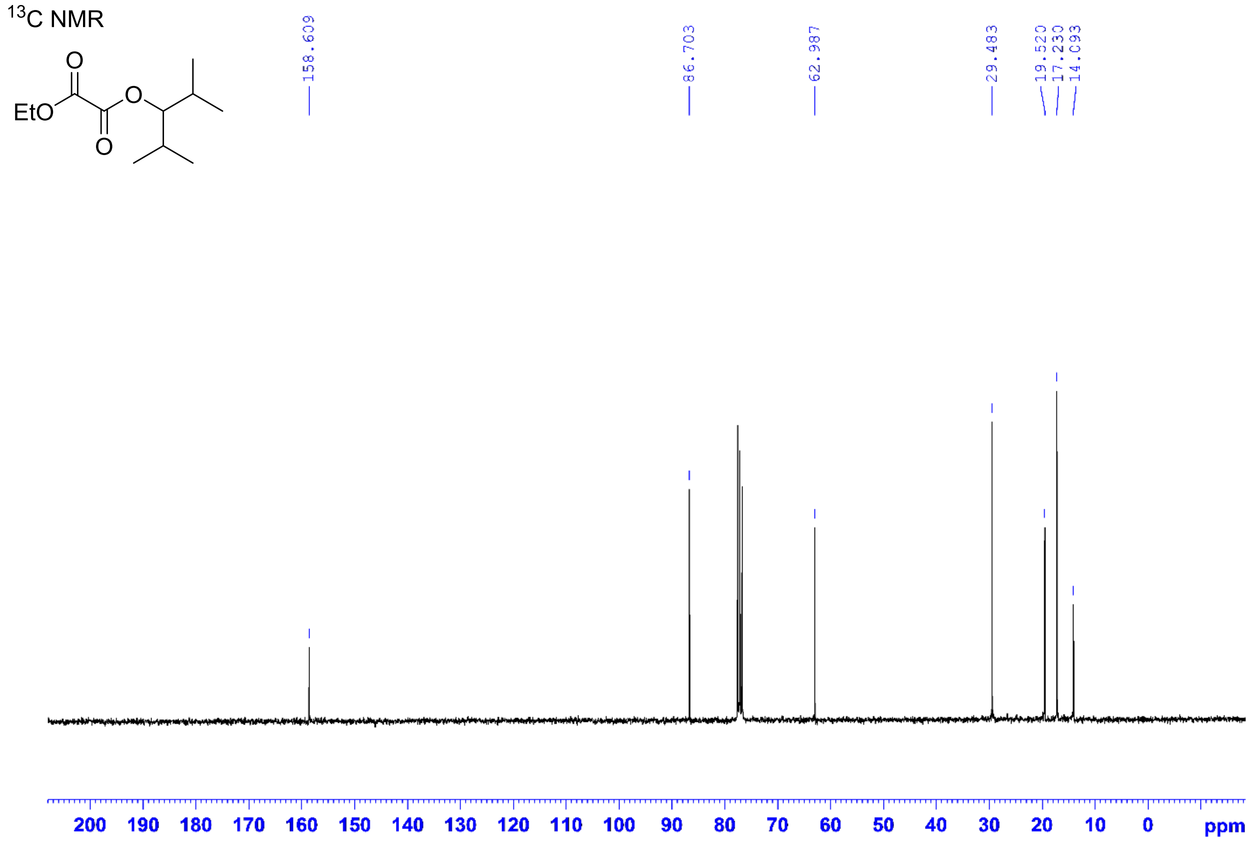

^{13}C NMR
EtO
O
158.609
86.703
62.987
29.483
19.520 17.230 14.093
200 190 180 170 160 150 140 130 120 110 100 90 80 70 60 50 40 30 20 10 0 ppm

^{1}H NMR

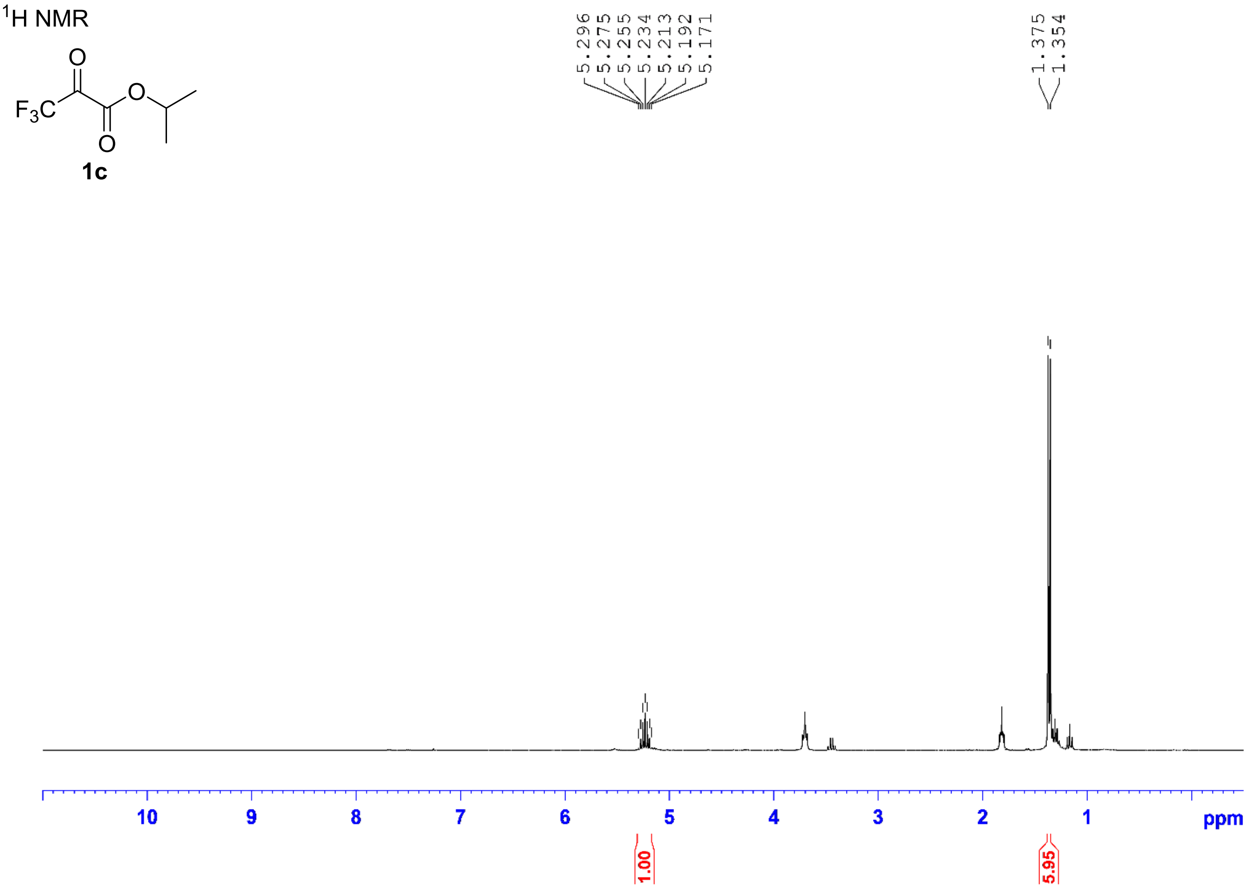

^{13}C NMR

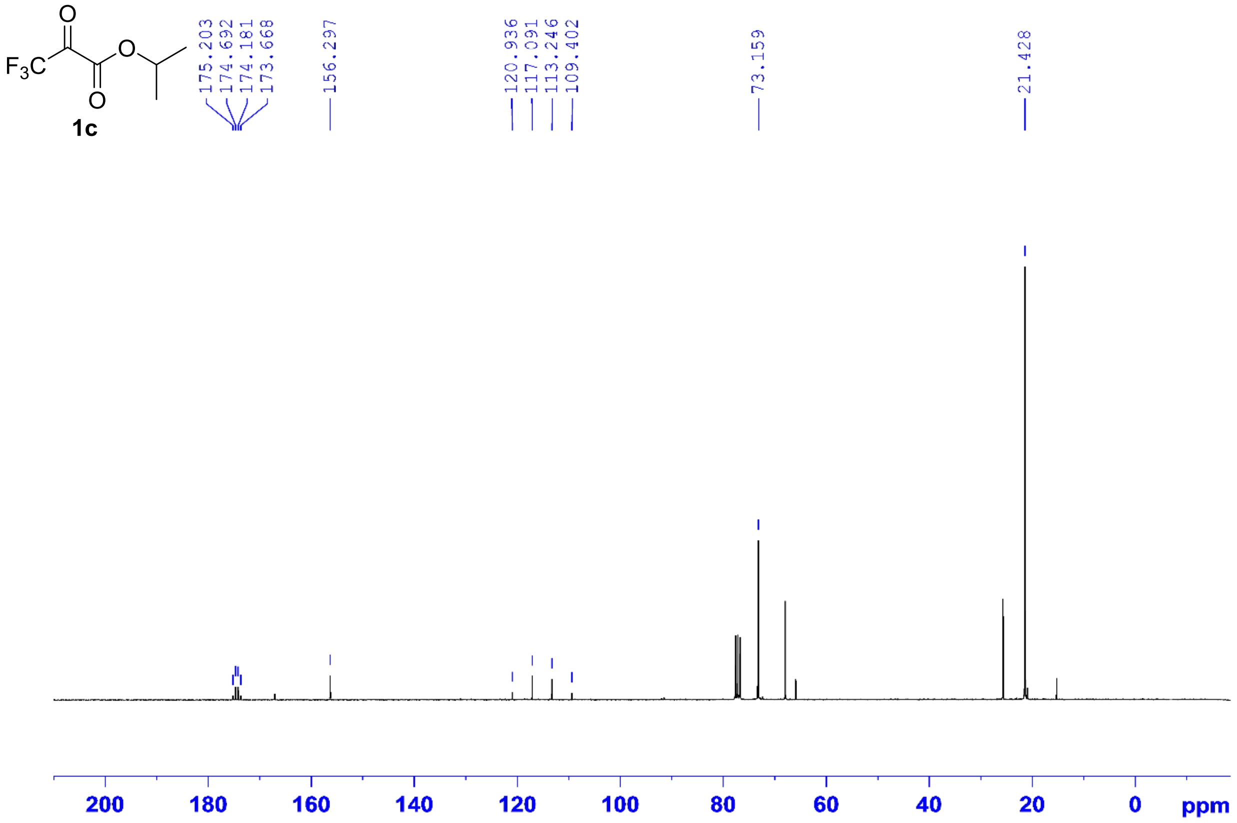

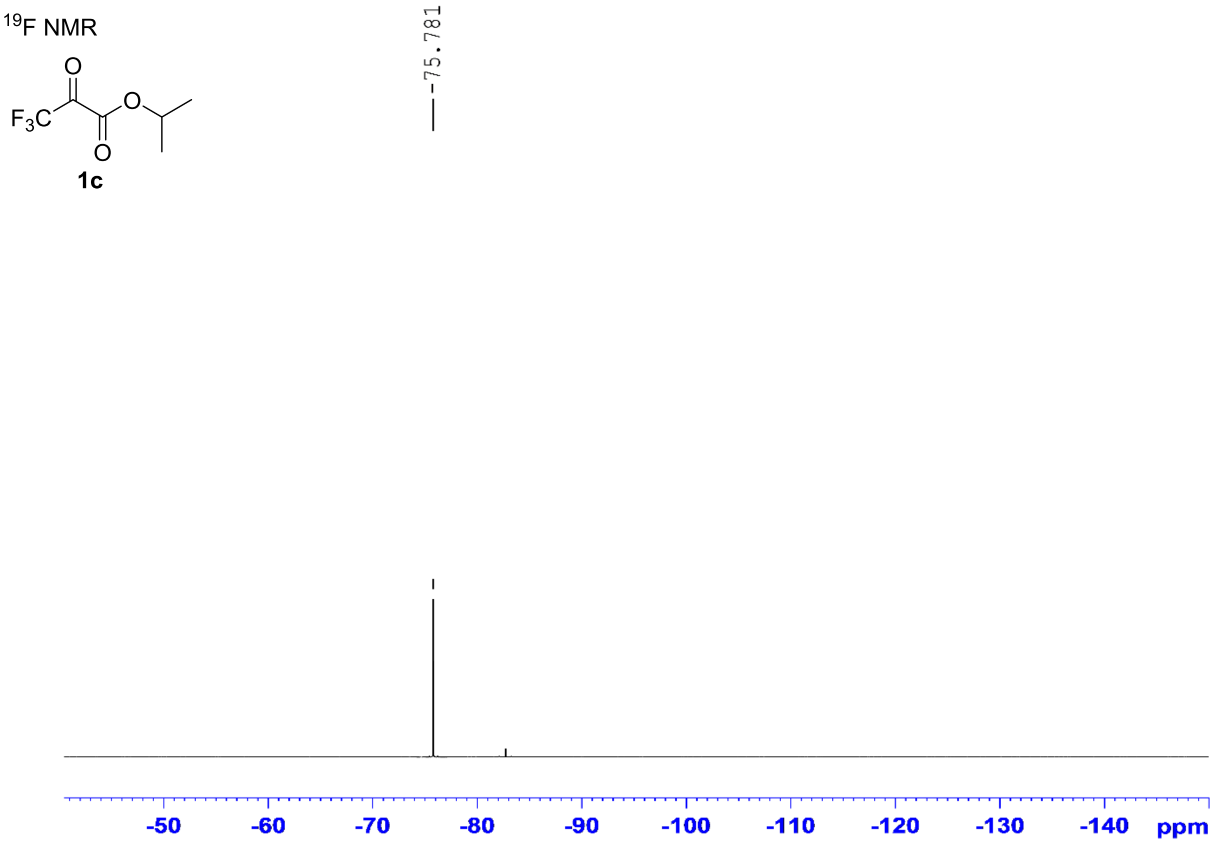
19F NMR
F3C
O
O
O
1c
-75.781
-50
-60
-70
-80
-90
-100
-110
-120
-130
-140
ppm

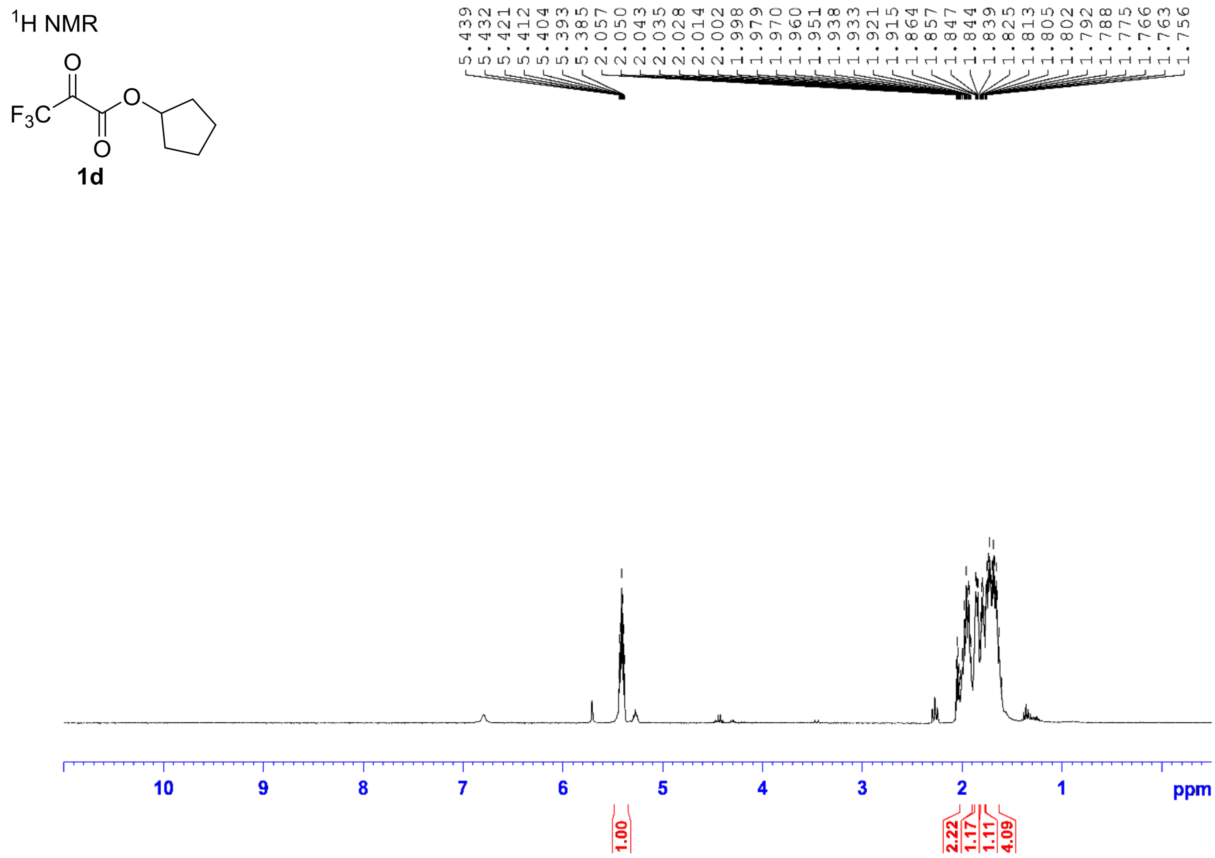
1H NMR
F3C
O
O
O
1d
5.439
5.432
5.421
5.412
5.404
5.393
5.385
2.057
2.050
2.043
2.035
2.028
2.014
2.002
1.998
1.979
1.970
1.960
1.951
1.938
1.933
1.921
1.915
1.864
1.857
1.847
1.844
1.839
1.825
1.813
1.805
1.802
1.792
1.788
1.775
1.766
1.763
1.756
10
9
8
7
6
5
4
3
2
1
ppm
1.00
2.22
1.17
1.11
4.09

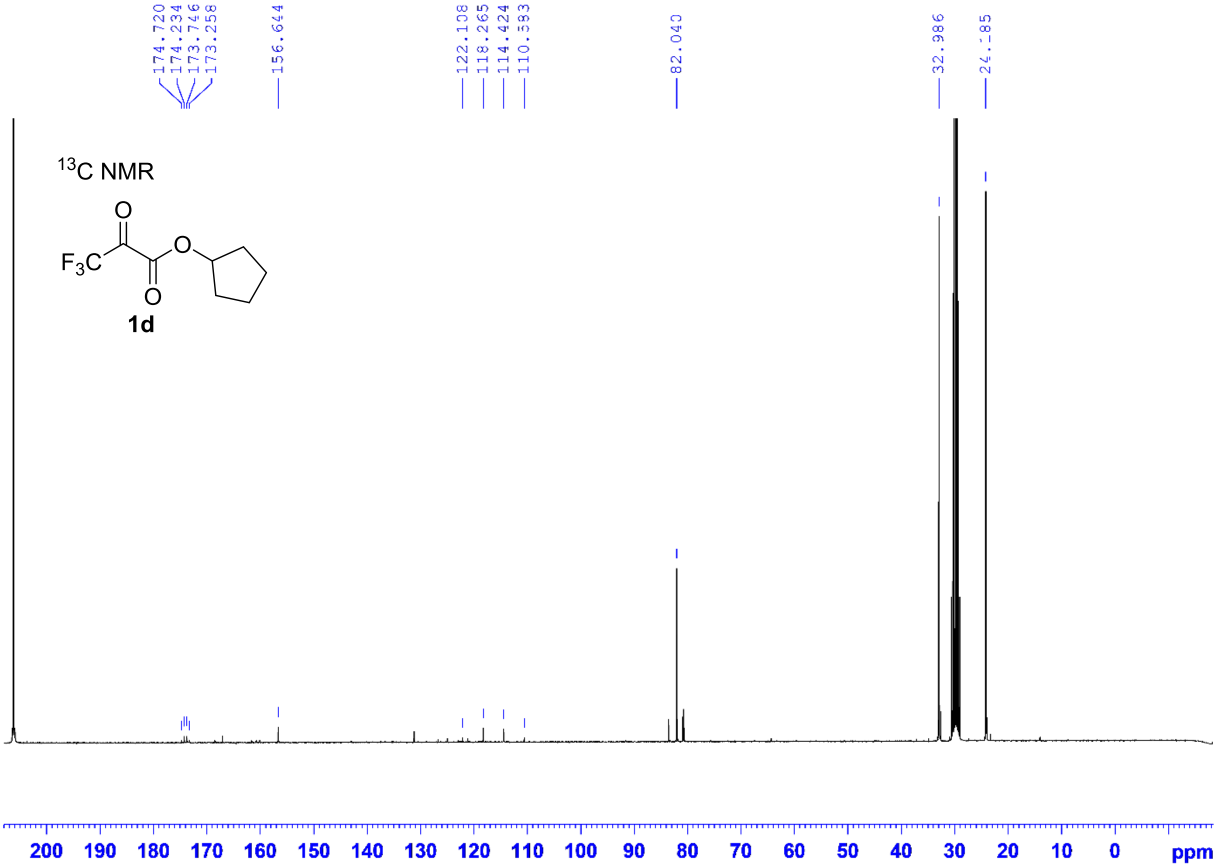
174.720
174.234
173.746
173.258
156.644
122.108
118.265
114.424
110.583
82.040
32.986
13C NMR
F3C
O
O
O
1d
200 190 180 170 160 150 140 130 120 110 100 90 80 70 60 50 40 30 20 10 0 ppm

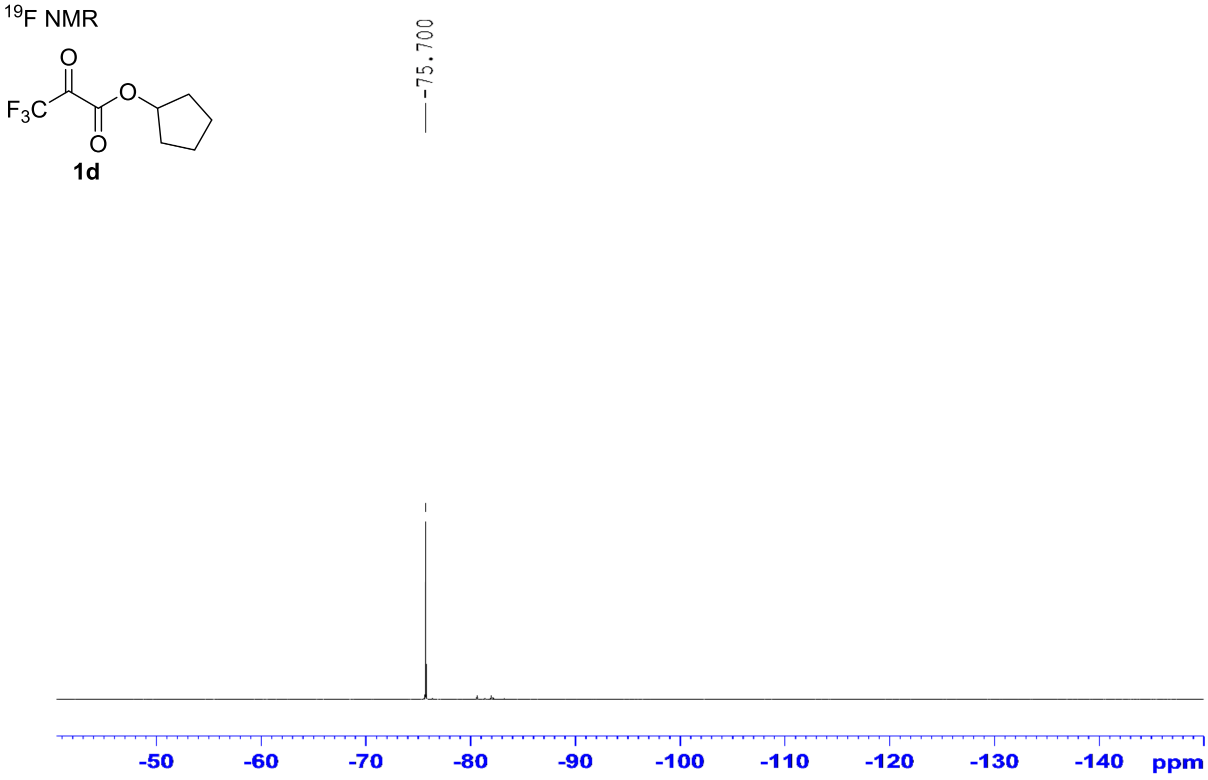
-75.700
19F NMR
F3C
O
O
O
1d
-50 -60 -70 -80 -90 -100 -110 -120 -130 -140 ppm

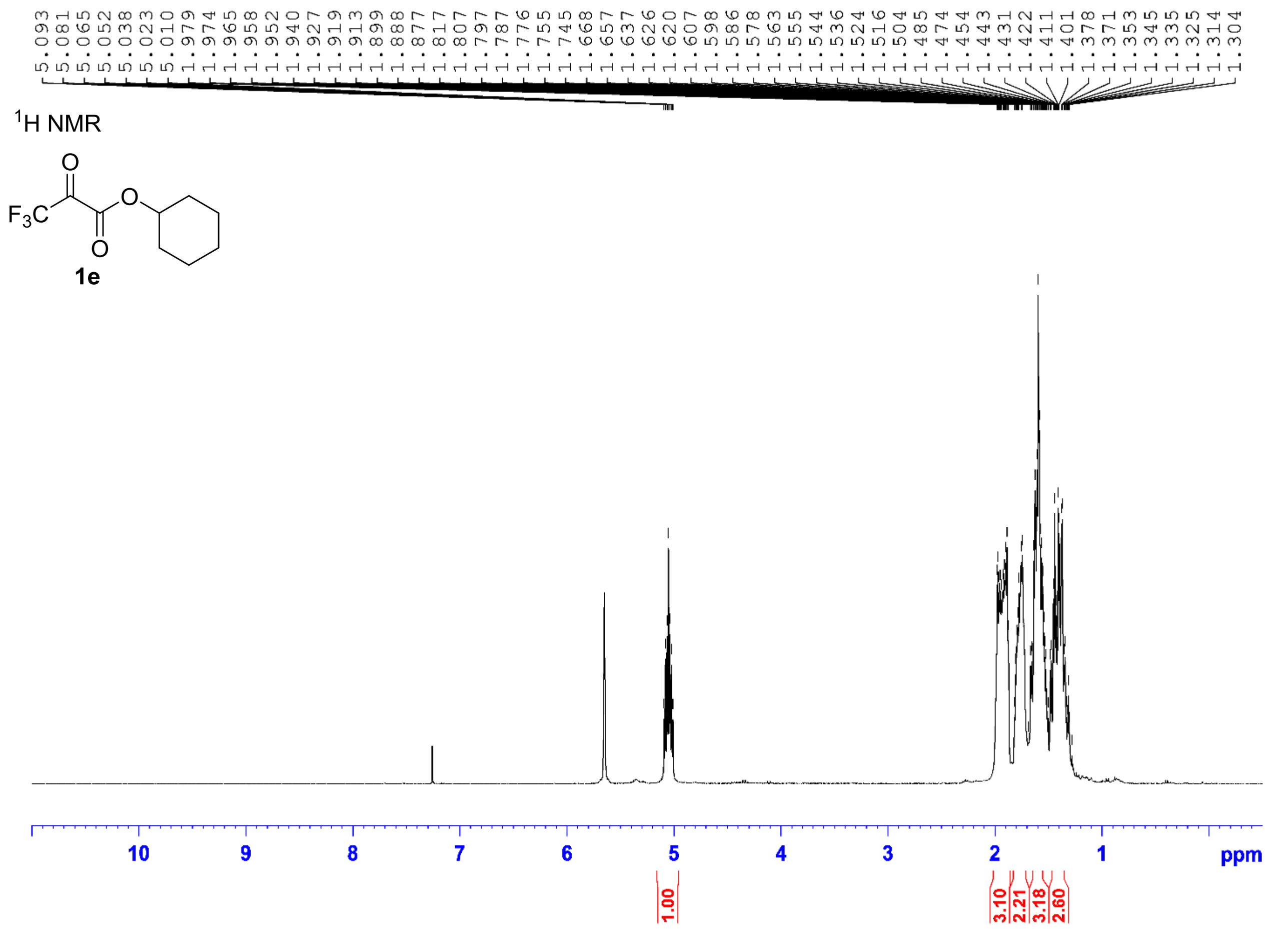
5.093
5.081
5.065
5.052
5.038
5.023
5.010
1.979
1.974
1.965
1.958
1.952
1.940
1.927
1.919
1.913
1.899
1.888
1.877
1.817
1.807
1.797
1.787
1.776
1.755
1.745
1.668
1.657
1.637
1.626
1.620
1.607
1.598
1.586
1.578
1.563
1.555
1.544
1.536
1.524
1.516
1.504
1.485
1.474
1.454
1.443
1.431
1.422
1.411
1.401
1.378
1.371
1.353
1.345
1.335
1.325
1.314
1.304
^{1}H NMR
O
F_3C
O
O
1e
10
9
8
7
6
5
4
3
2
1
ppm
1.00
3.10
2.21
3.18
2.60

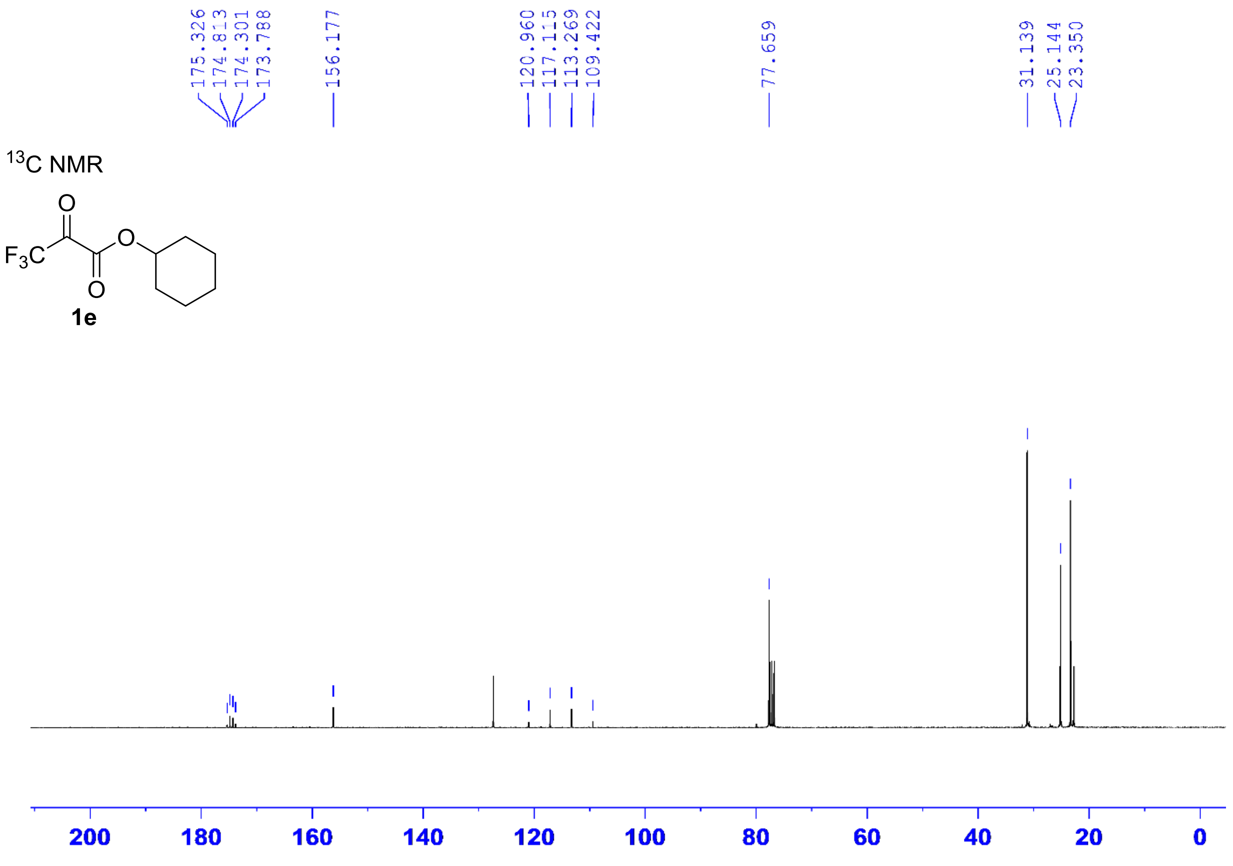
175.326
174.813
174.301
173.788
156.177
120.960
117.115
113.269
109.422
77.659
31.139
25.144
23.350
^{13}C NMR
O
F_3C
O
O
1e
200
180
160
140
120
100
80
60
40
20
0

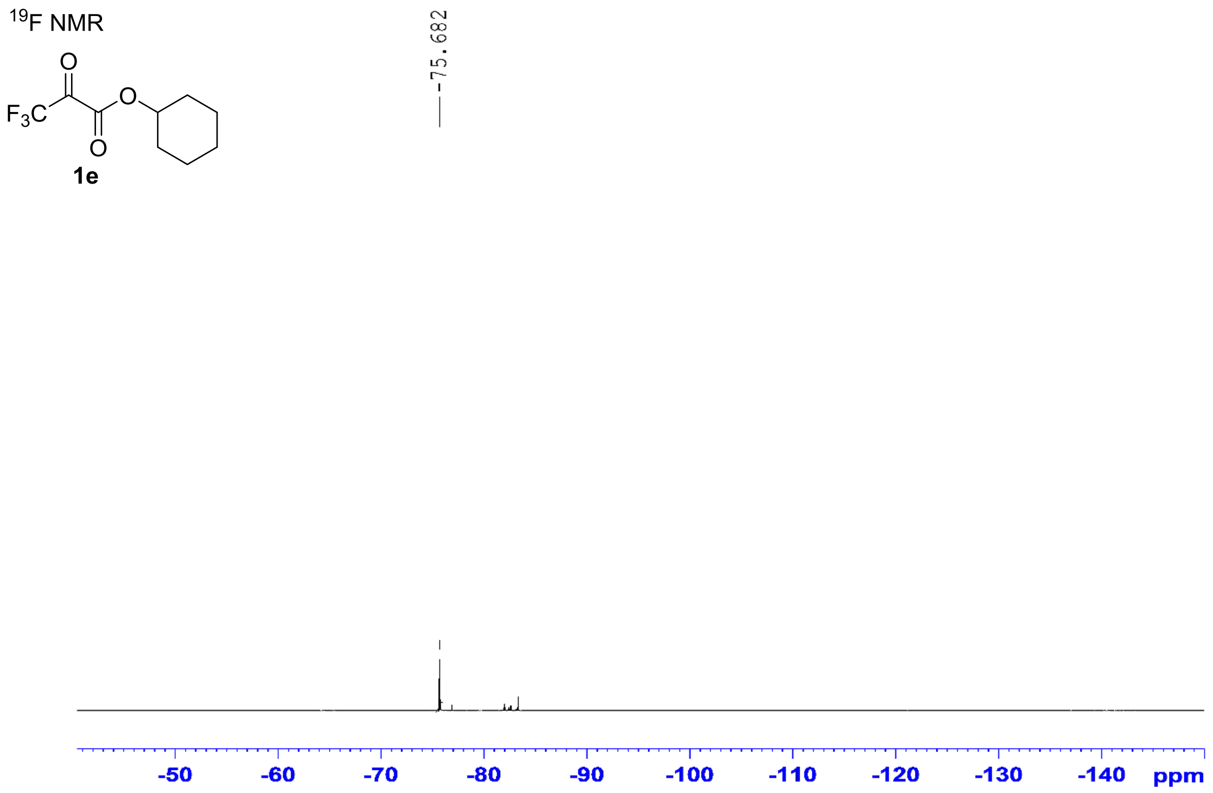

19F NMR
F3C
O
O
O
1e
-75.682
-50
-60
-70
-80
-90
-100
-110
-120
-130
-140
ppm

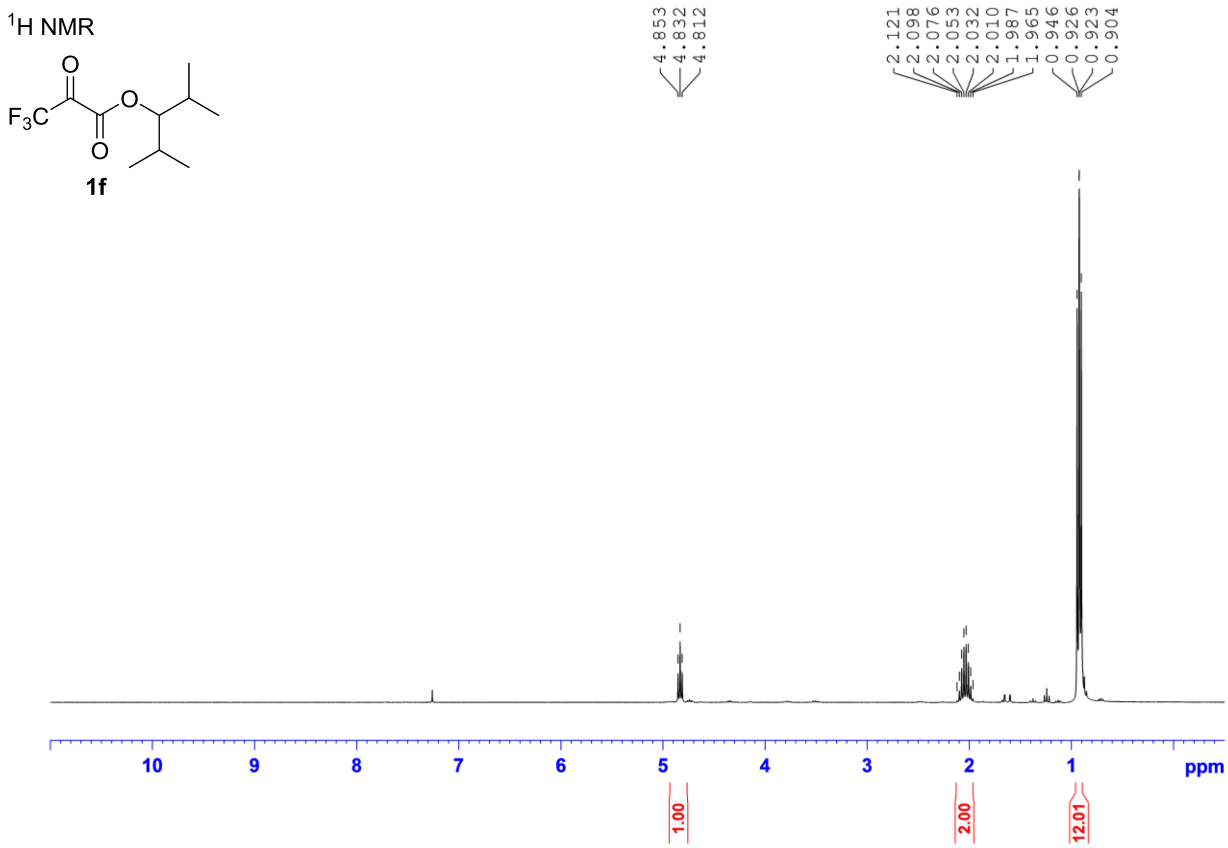

1H NMR
F3C
O
O
O
1f
4.853
4.832
4.812
2.121
2.098
2.076
2.053
2.032
2.010
1.987
1.965
0.946
0.926
0.923
0.904
10
9
8
7
6
5
4
3
2
1
ppm
1.00
2.00
12.01

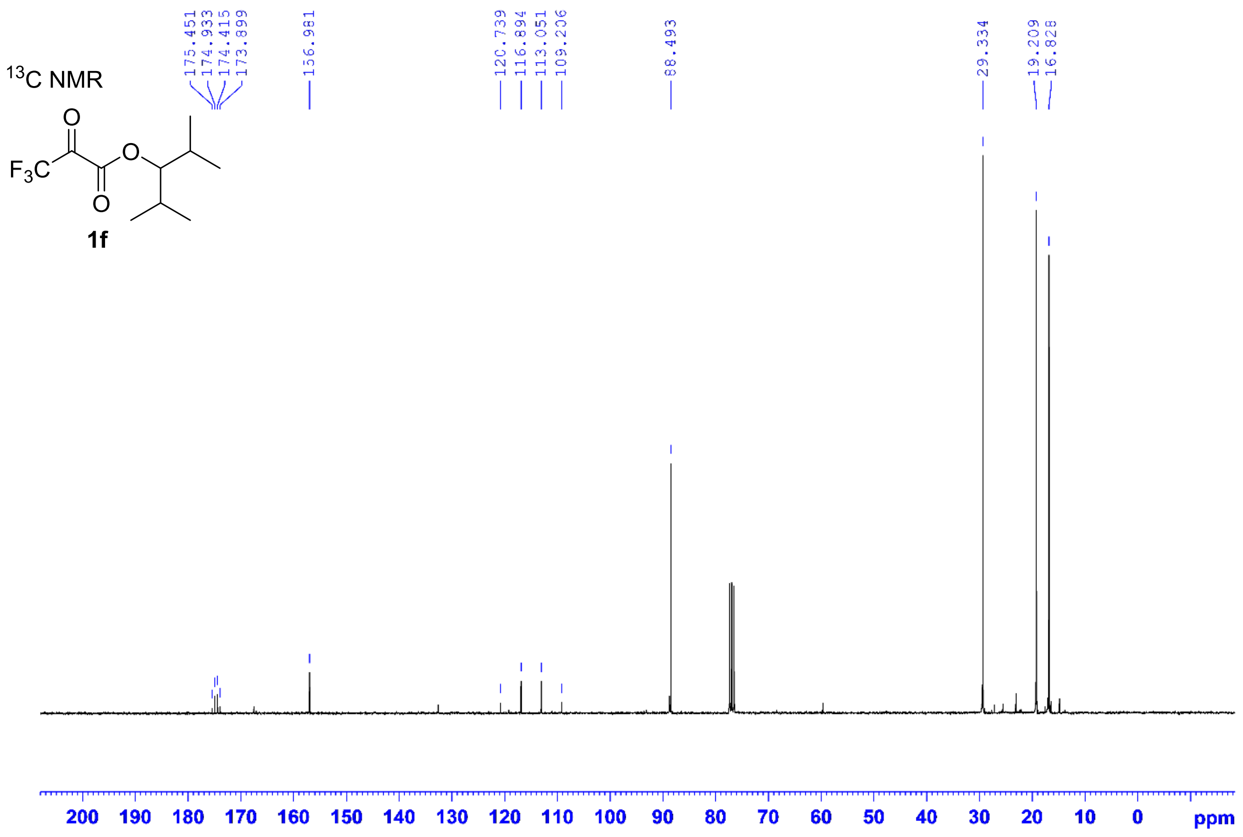

13C NMR
F3C
O
O
O
1f
175.451
174.933
174.415
173.899
156.981
120.739
116.894
113.051
109.206
88.493
29.334
19.209
16.828
200 190 180 170 160 150 140 130 120 110 100 90 80 70 60 50 40 30 20 10 0 ppm

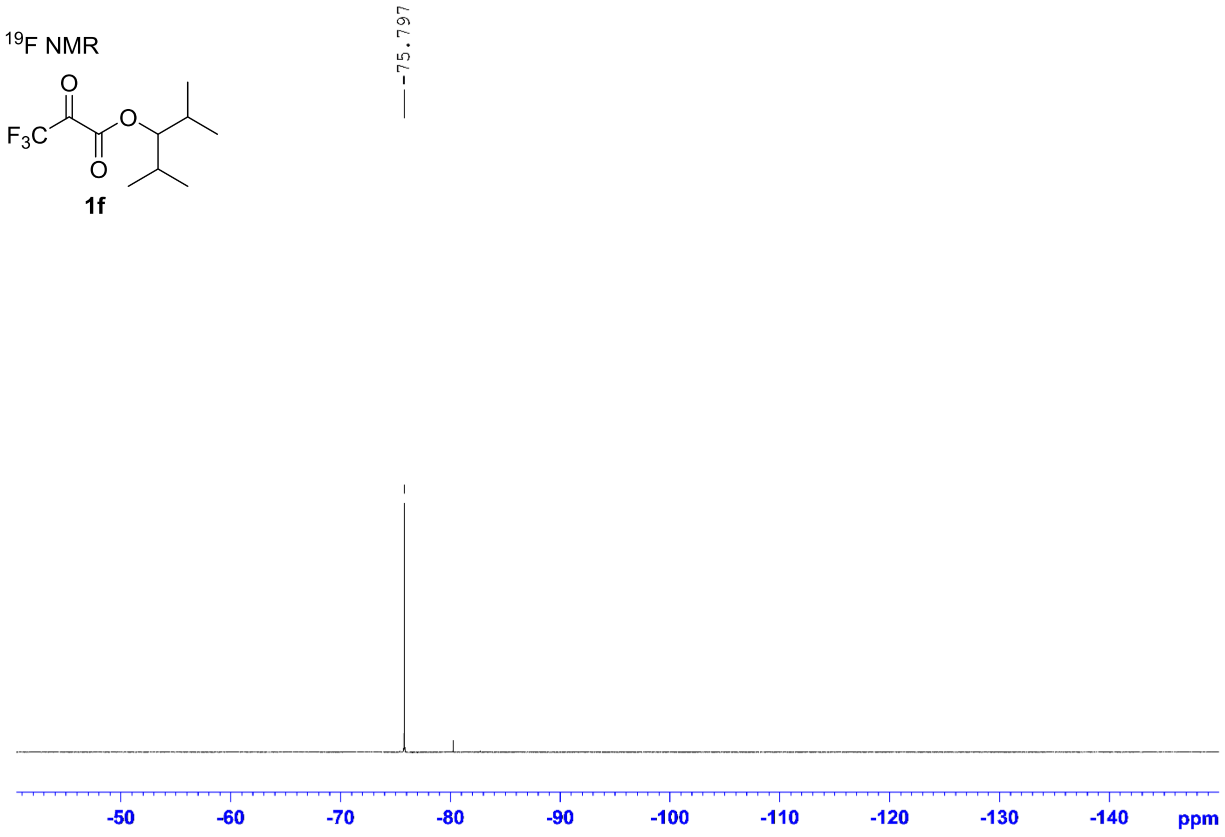

19F NMR
F3C
O
O
O
1f
-75.797
-50 -60 -70 -80 -90 -100 -110 -120 -130 -140 ppm

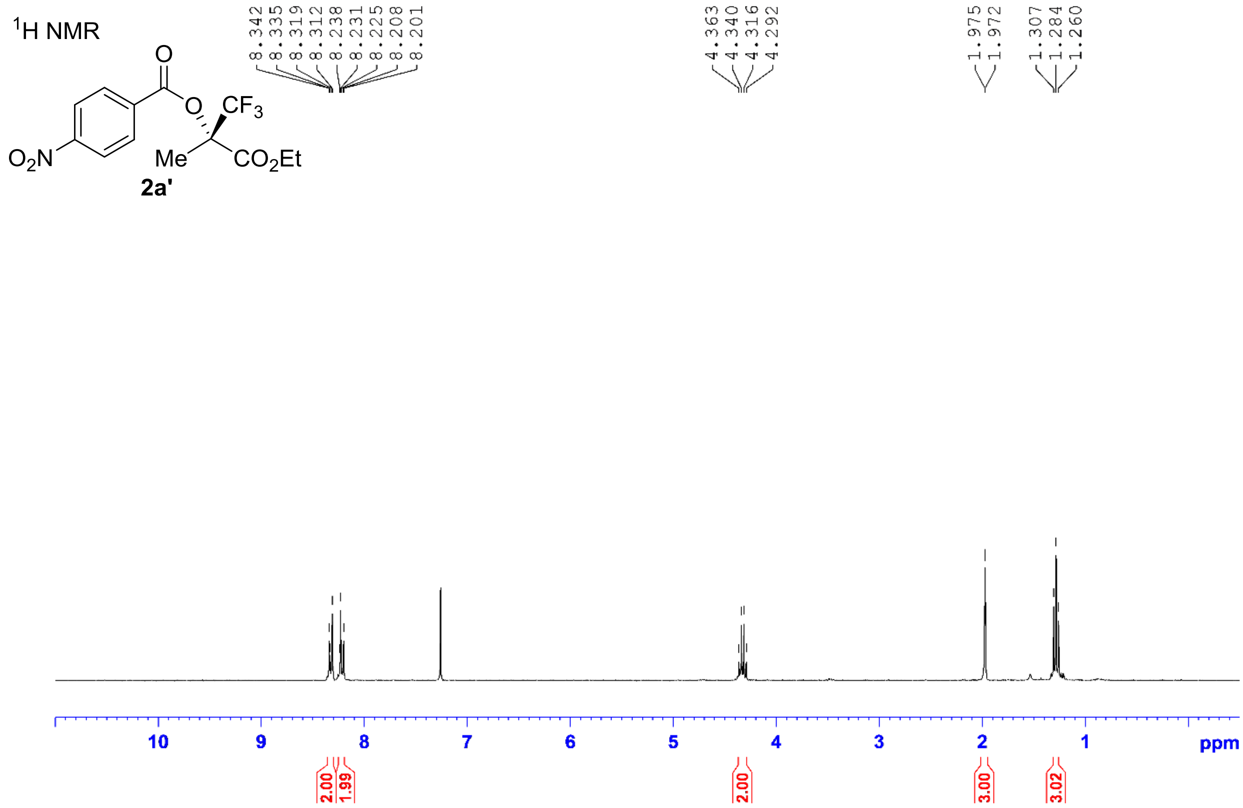

^{1}H NMR
2a'
8.342 8.335 8.319 8.312 8.238 8.231 8.225 8.208 8.201
4.363 4.340 4.316 4.292
1.975 1.972
1.307 1.284 1.260
2.00 1.99 2.00 3.00 3.02
ppm

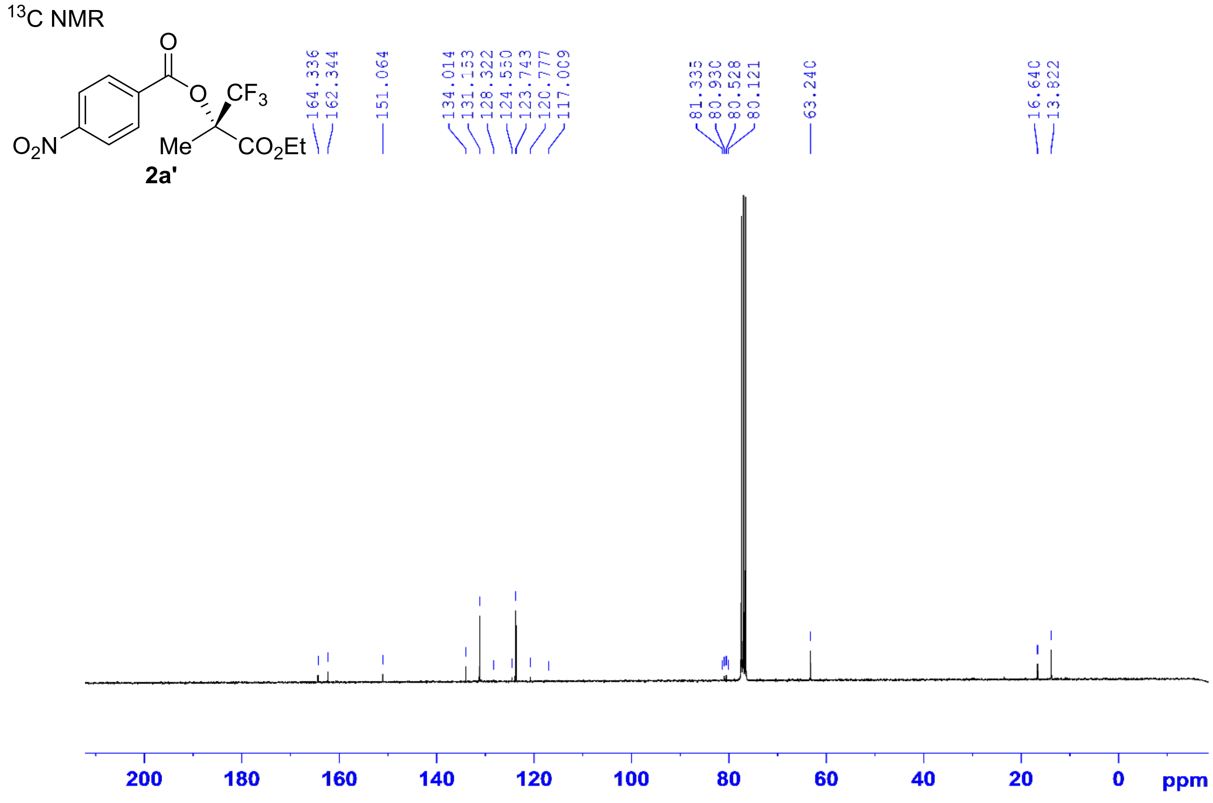

^{13}C NMR
2a'
164.336 162.344 151.064 134.014 131.153 128.322 124.550 123.743 120.777 117.009
81.335 80.930 80.528 80.121 63.240 16.640 13.822
ppm

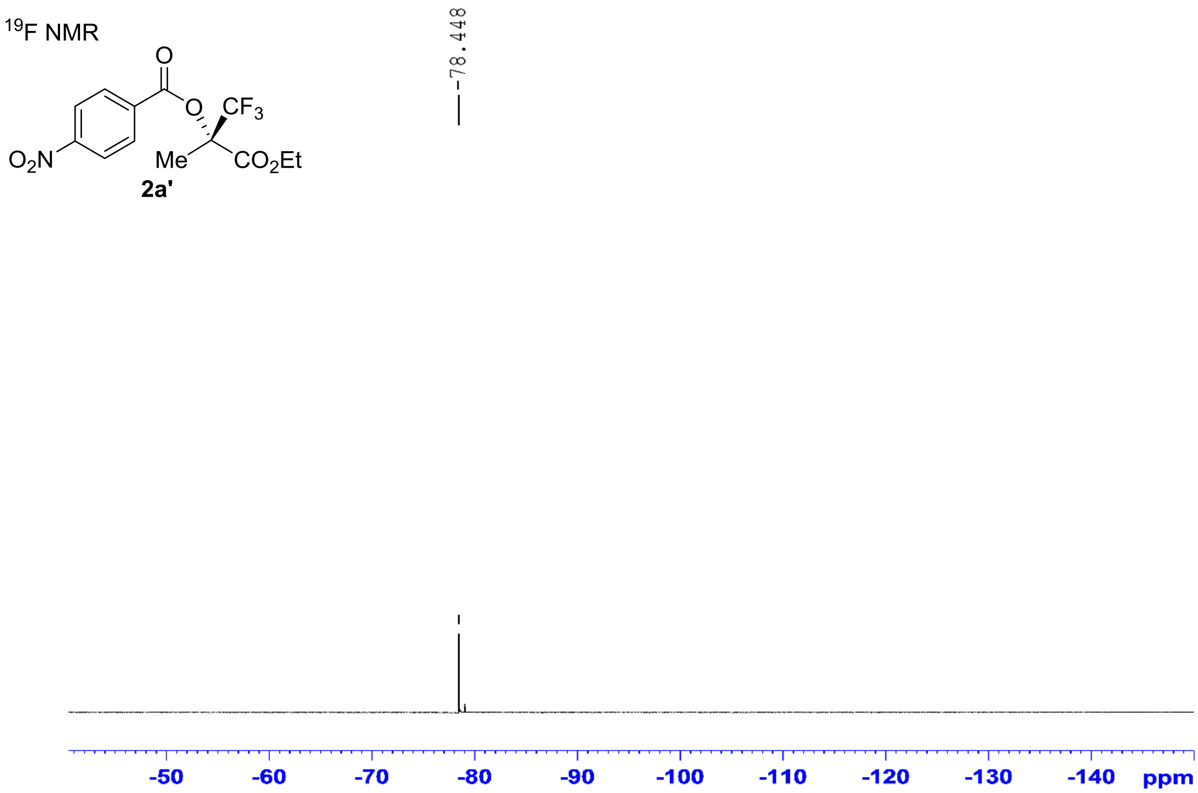

^{19}F NMR
2a'
-78.448
-50 -60 -70 -80 -90 -100 -110 -120 -130 -140 ppm

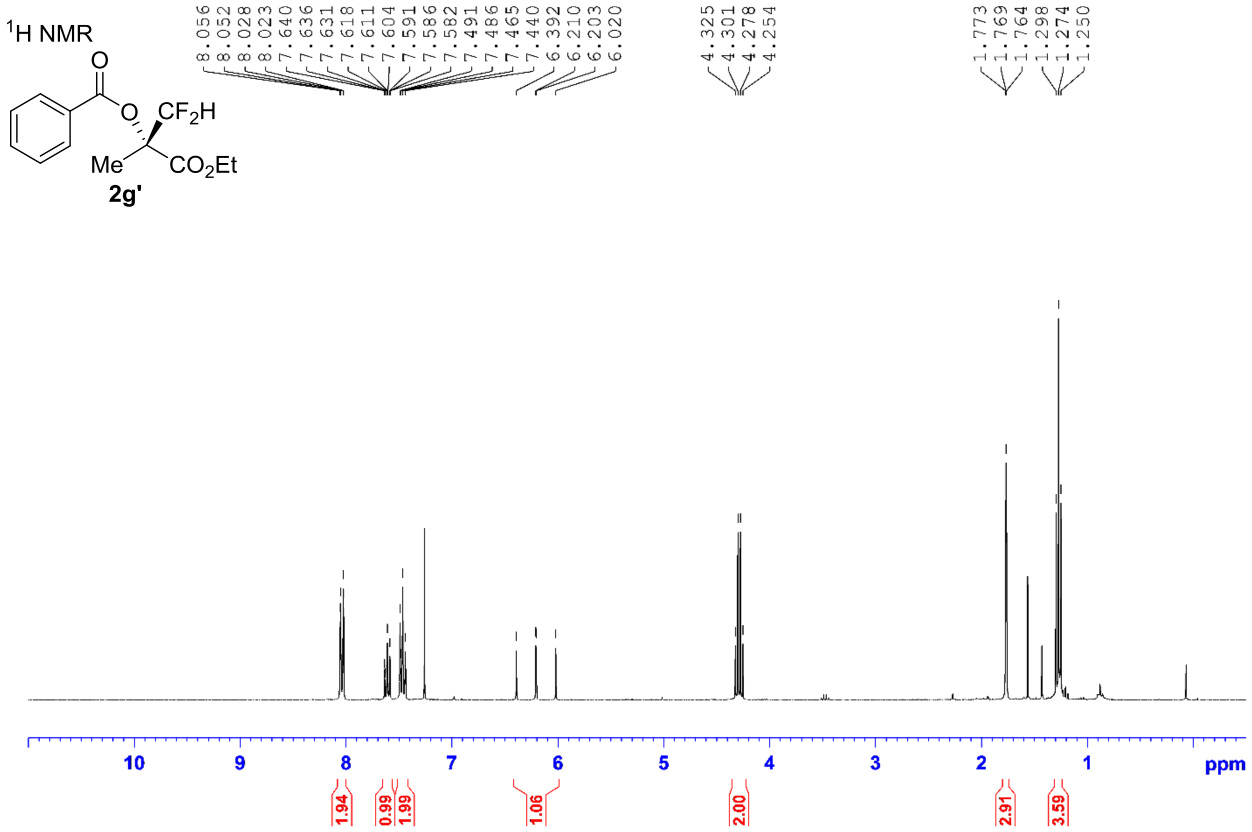

^{1}H NMR
2g'
8.056 8.052 8.028 8.023 7.640 7.636 7.631 7.618 7.611 7.604 7.591 7.586 7.582 7.491 7.486 7.465 7.440 6.392 6.210 6.203 6.020
4.325 4.301 4.278 4.254
1.773 1.769 1.764 1.298 1.274 1.250
10 9 8 7 6 5 4 3 2 1 ppm
1.94 0.99 1.99 1.06 2.00 2.91 3.59

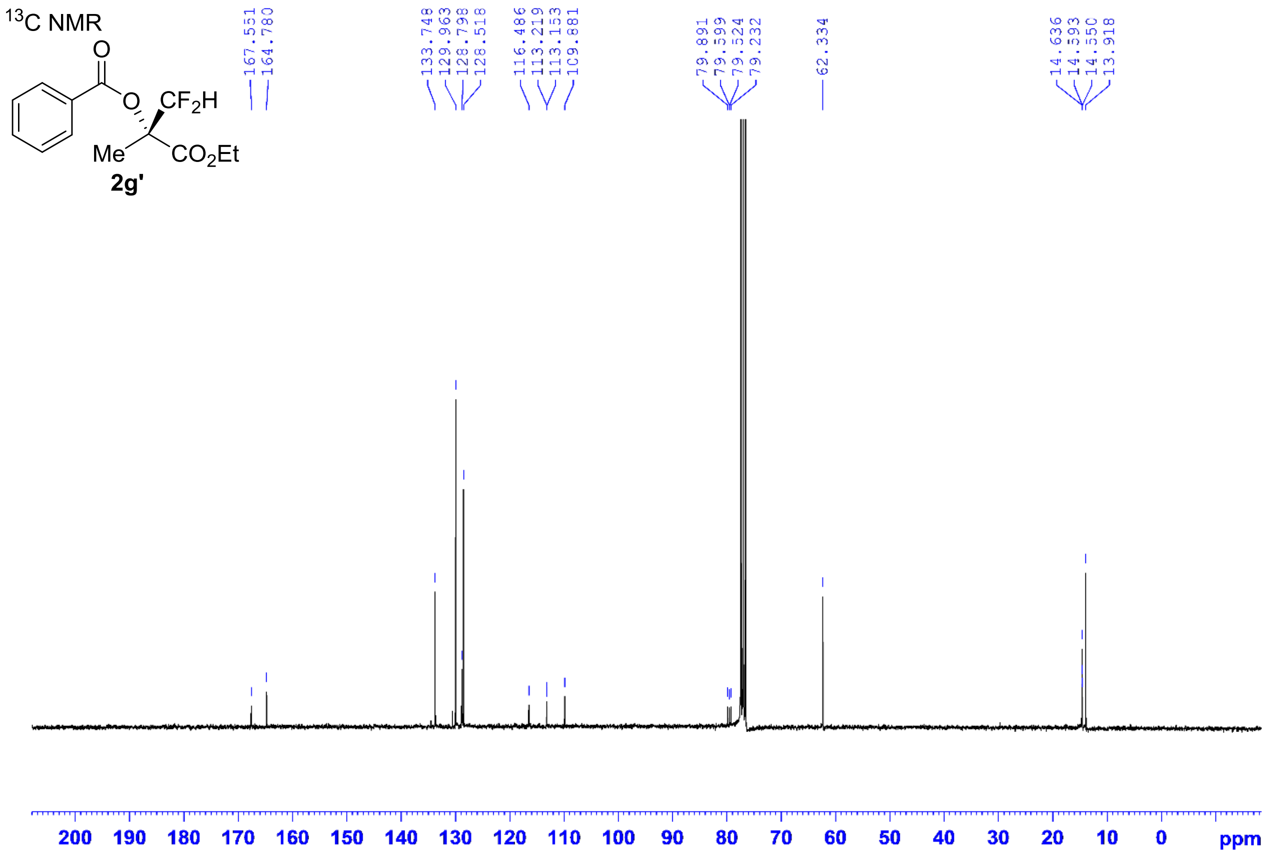
13C NMR
O
O
CF2H
Me
CO2Et
2g'
167.551
164.780
133.748
129.963
128.798
128.518
116.486
113.219
113.153
109.881
79.891
79.599
79.524
79.232
62.334
14.636
14.593
14.550
13.918
200 190 180 170 160 150 140 130 120 110 100 90 80 70 60 50 40 30 20 10 0 ppm

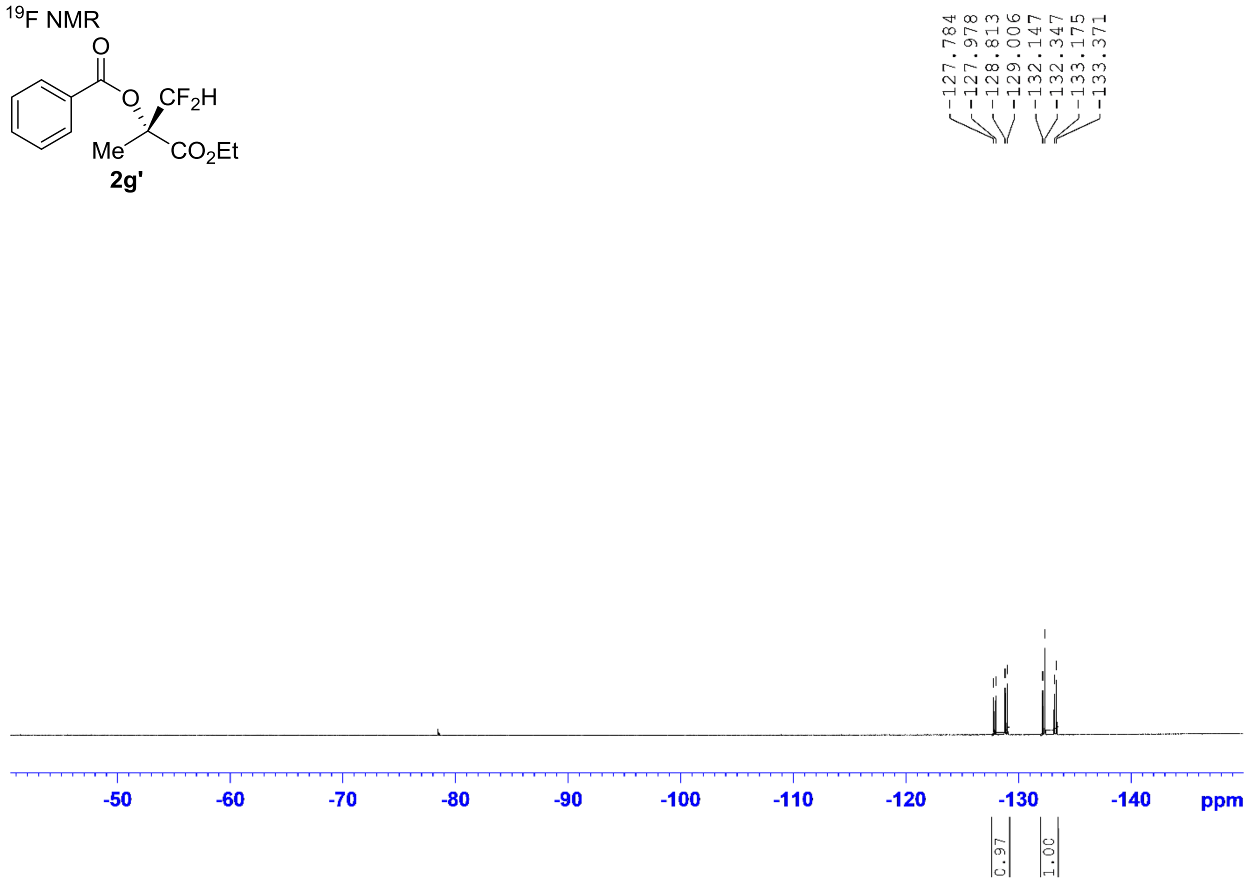
19F NMR
O
O
CF2H
Me
CO2Et
2g'
-127.784
-127.978
-128.813
-129.006
-132.147
-132.347
-133.175
-133.371
-50 -60 -70 -80 -90 -100 -110 -120 -130 -140 ppm
0.97
1.00

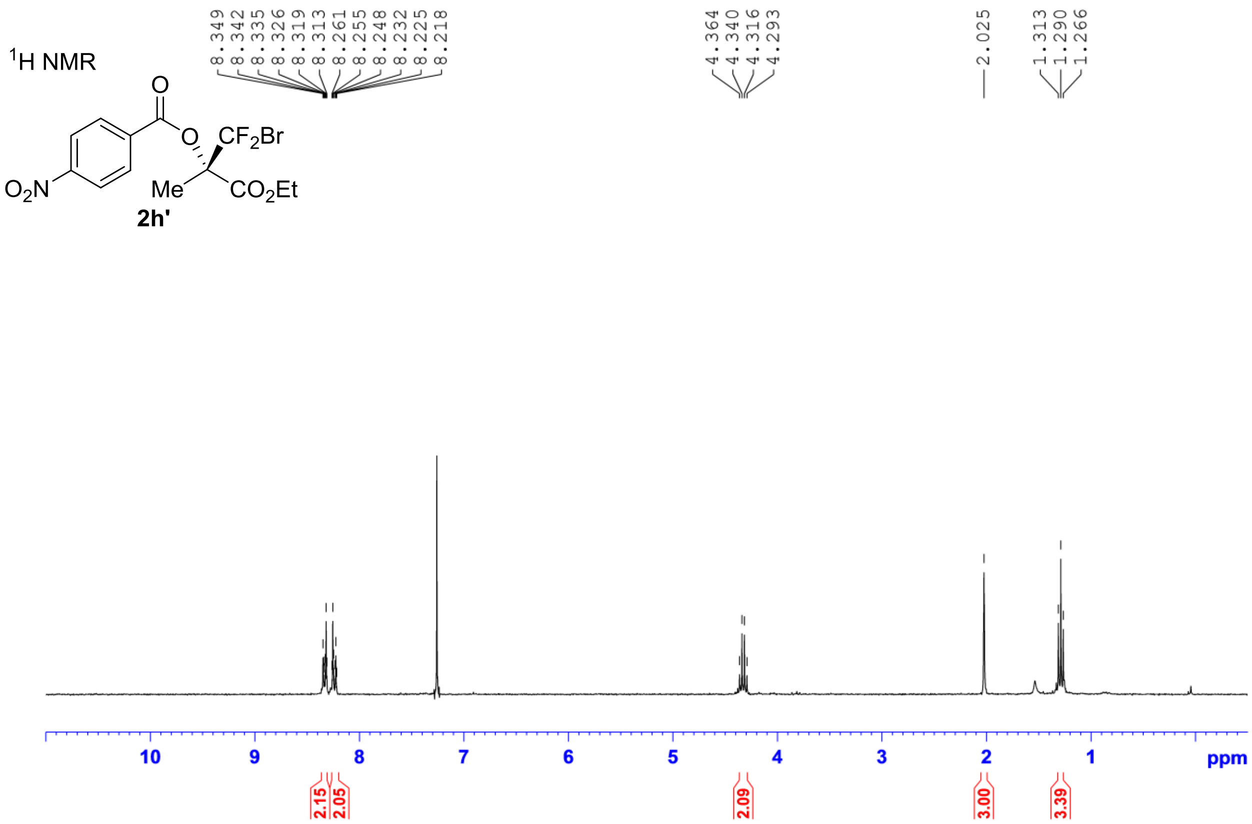
1H NMR
O
O
CF2Br
O2N
Me
CO2Et
2h'
8.349
8.342
8.335
8.326
8.319
8.313
8.261
8.255
8.248
8.232
8.225
8.218
4.364
4.340
4.316
4.293
2.025
1.313
1.290
1.266
10
9
8
7
6
5
4
3
2
1
ppm
2.15
2.05
2.09
3.00
3.39

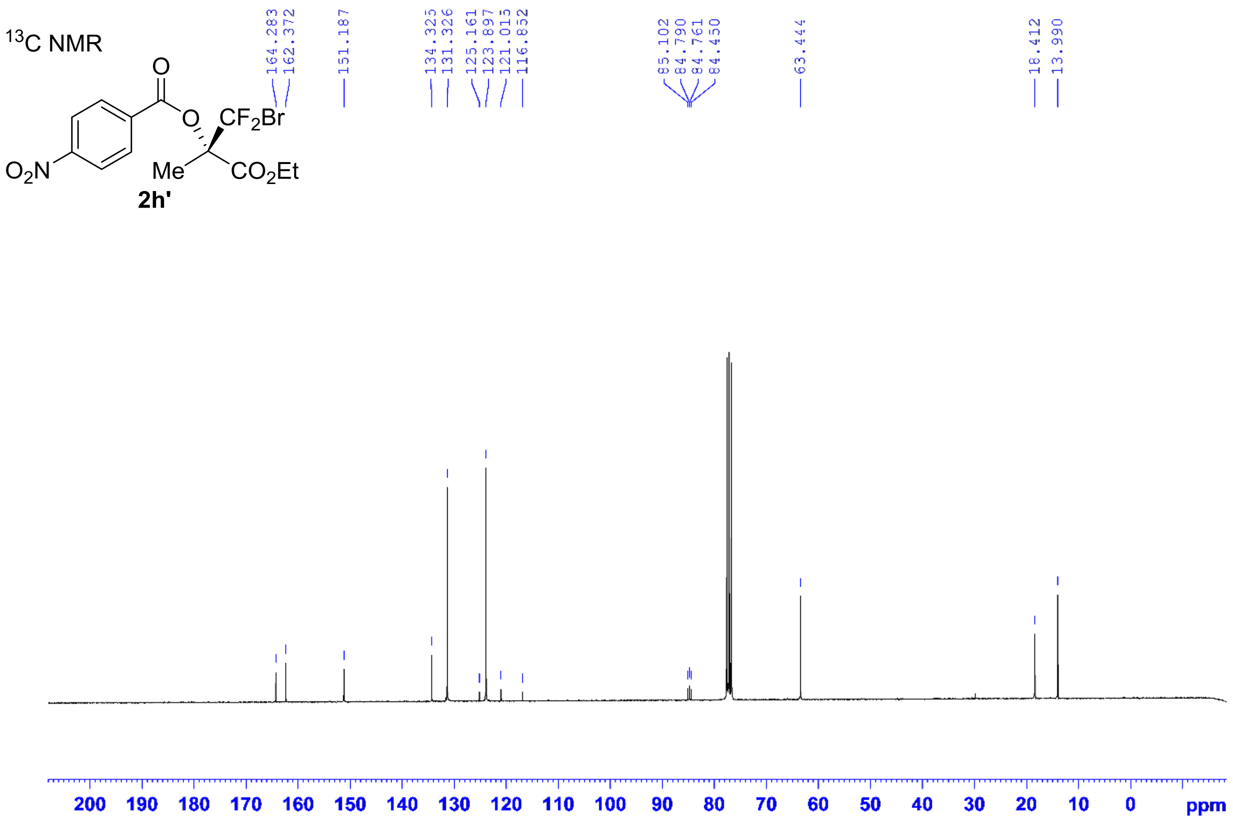
13C NMR
O
O
CF2Br
O2N
Me
CO2Et
2h'
164.283
162.372
151.187
134.325
131.326
125.161
123.897
121.015
116.832
85.102
84.790
84.761
84.450
63.444
18.412
13.990
200
190
180
170
160
150
140
130
120
110
100
90
80
70
60
50
40
30
20
10
0
ppm

^{19}F NMR

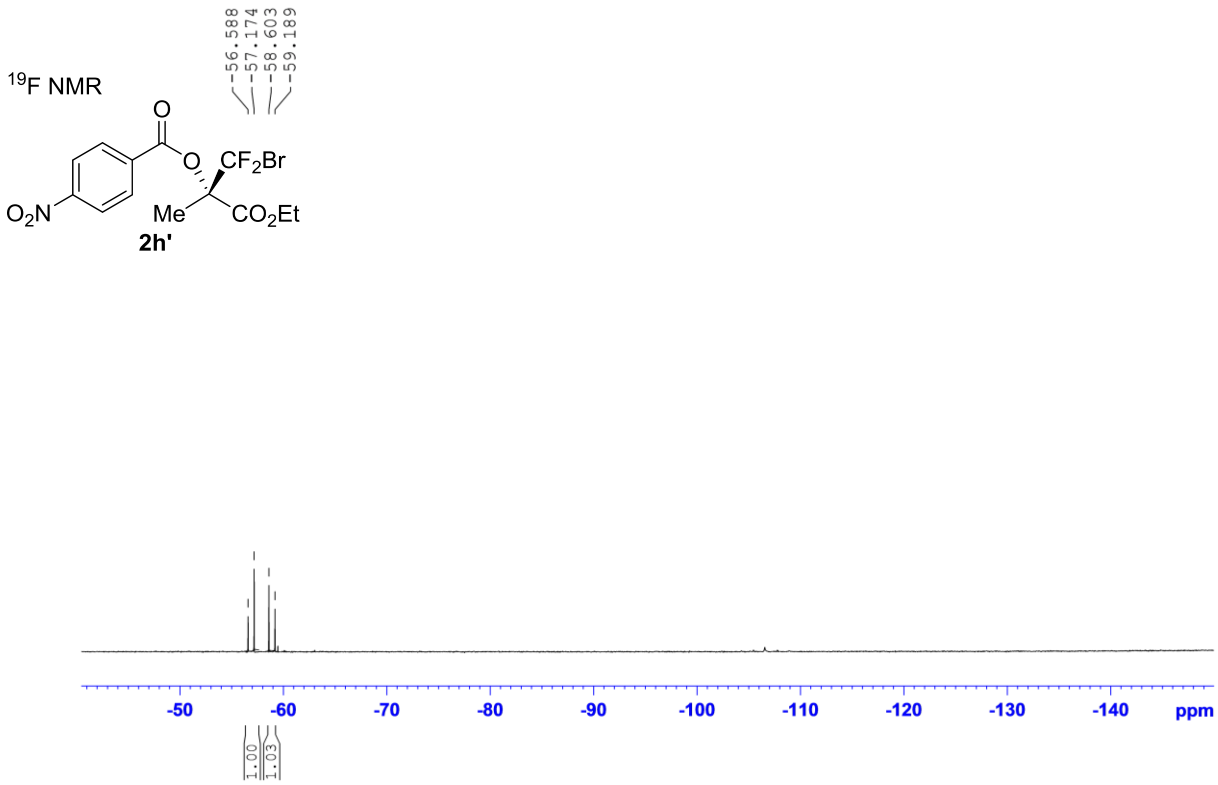

^{1}H NMR

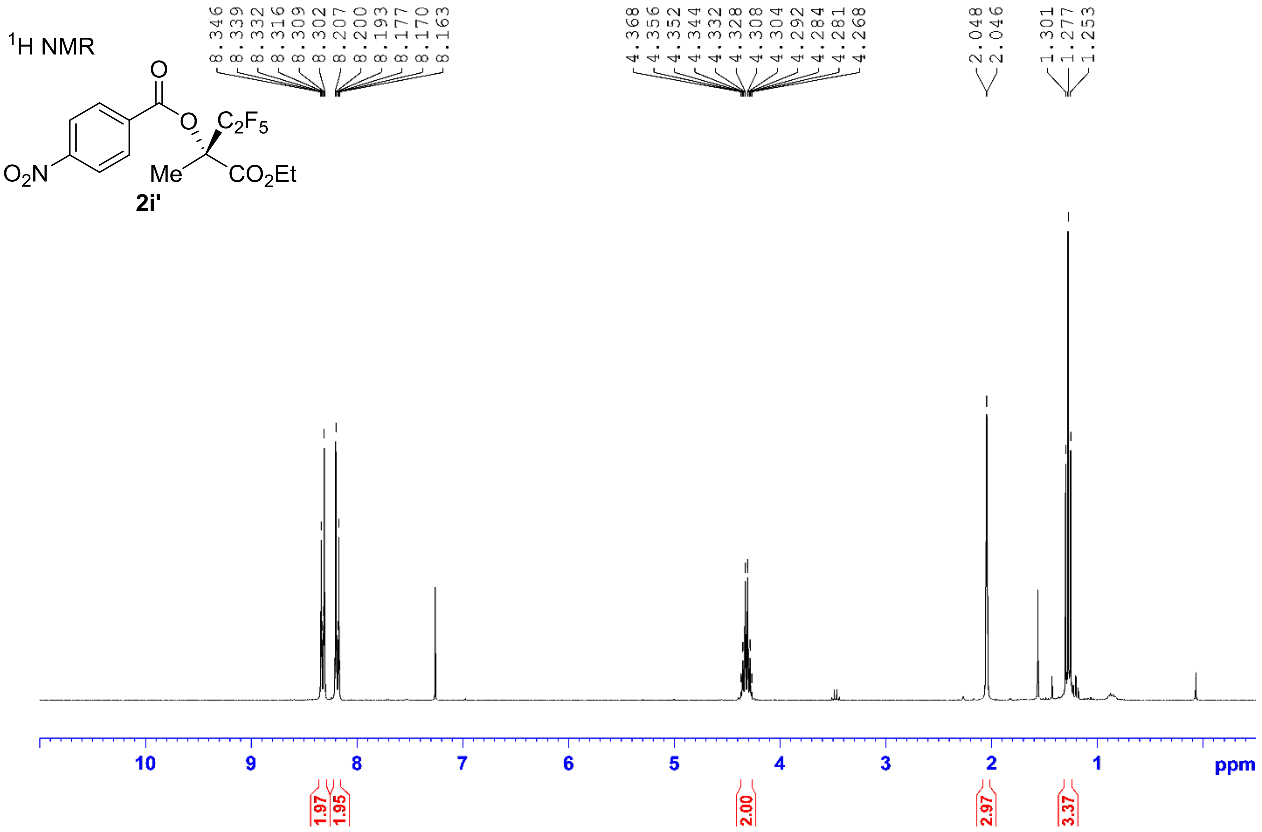

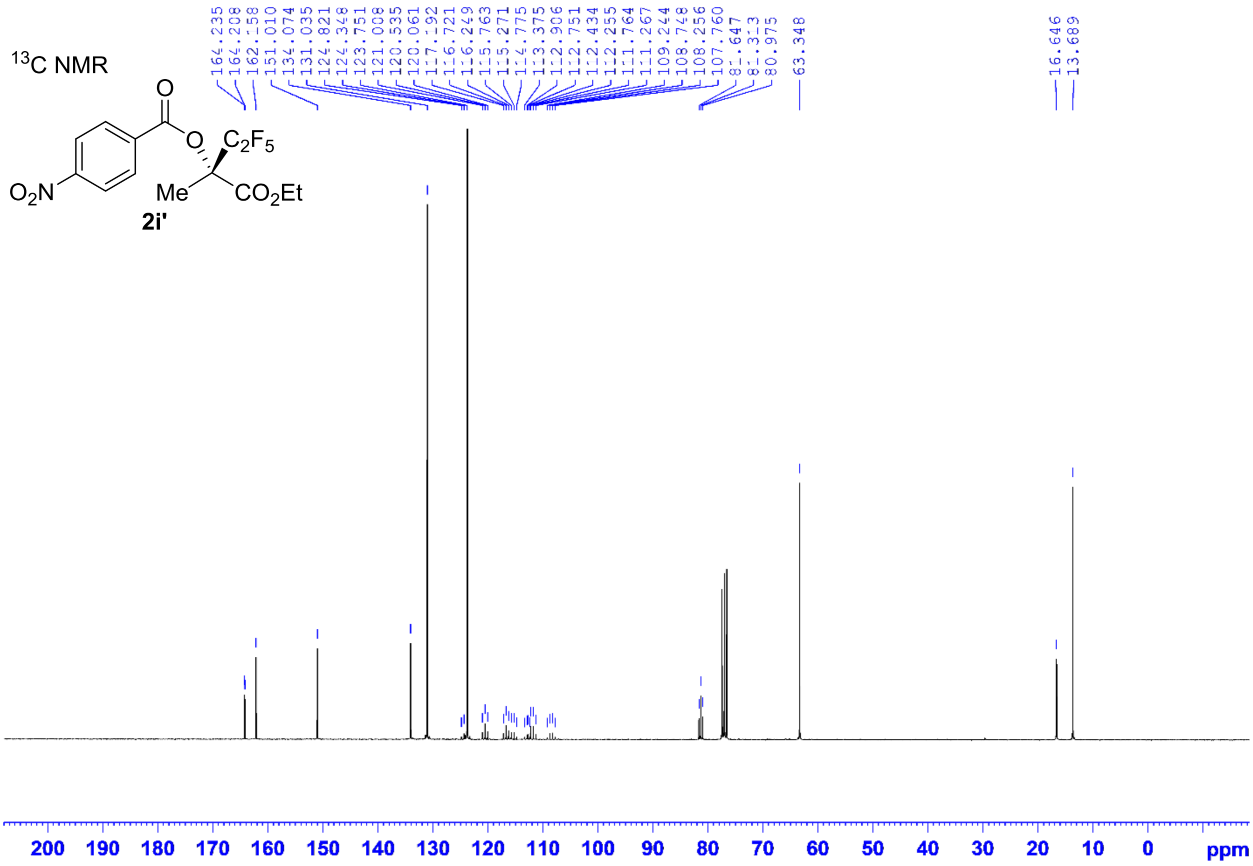

13C NMR
O
O
C2F5
O2N
Me
CO2Et
2i'
164.235
164.208
151.010
134.074
131.035
124.821
124.348
123.751
121.008
120.535
120.061
109.244
108.748
108.256
107.760
80.975
63.348
16.646
13.689
200 190 180 170 160 150 140 130 120 110 100 90 80 70 60 50 40 30 20 10 0 ppm

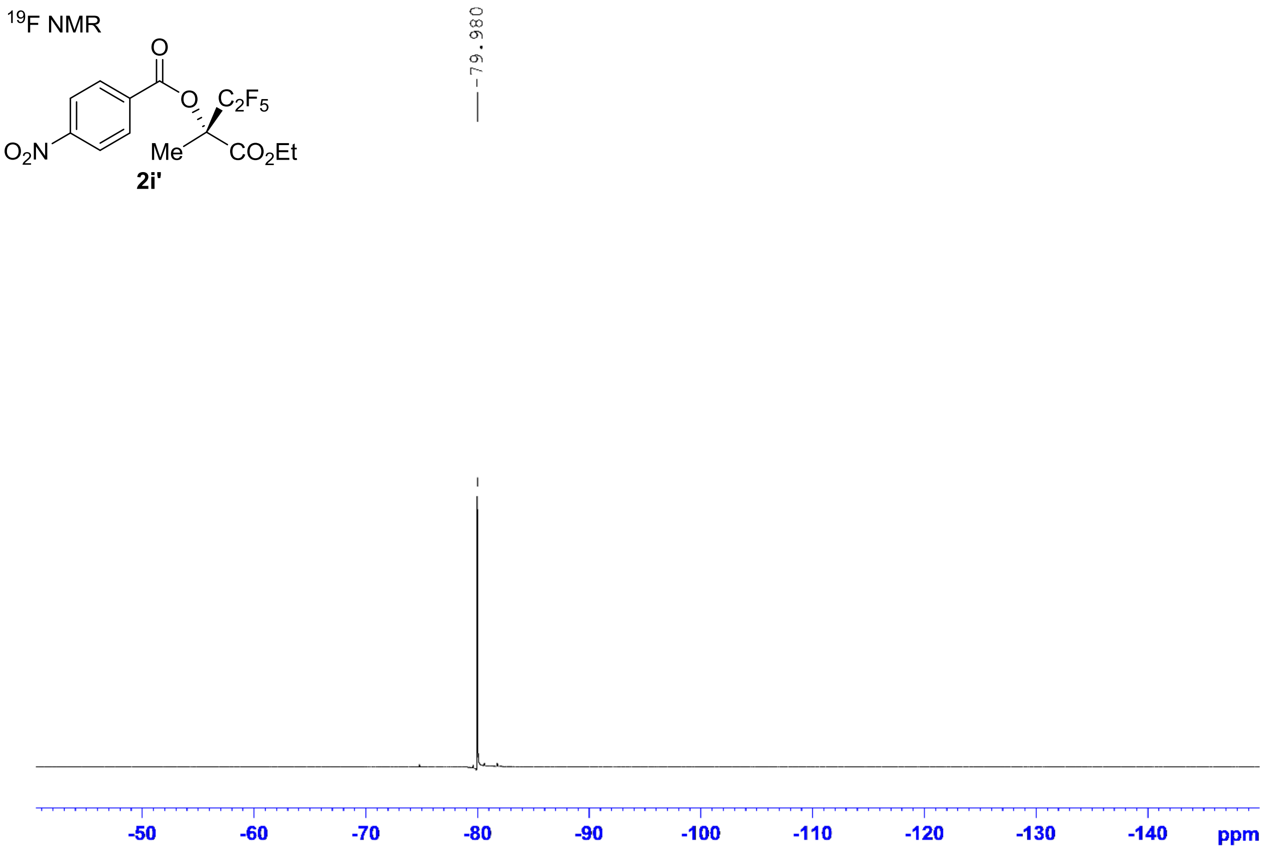

19F NMR
O
O
C2F5
O2N
Me
CO2Et
2i'
-79.980
-50 -60 -70 -80 -90 -100 -110 -120 -130 -140 ppm

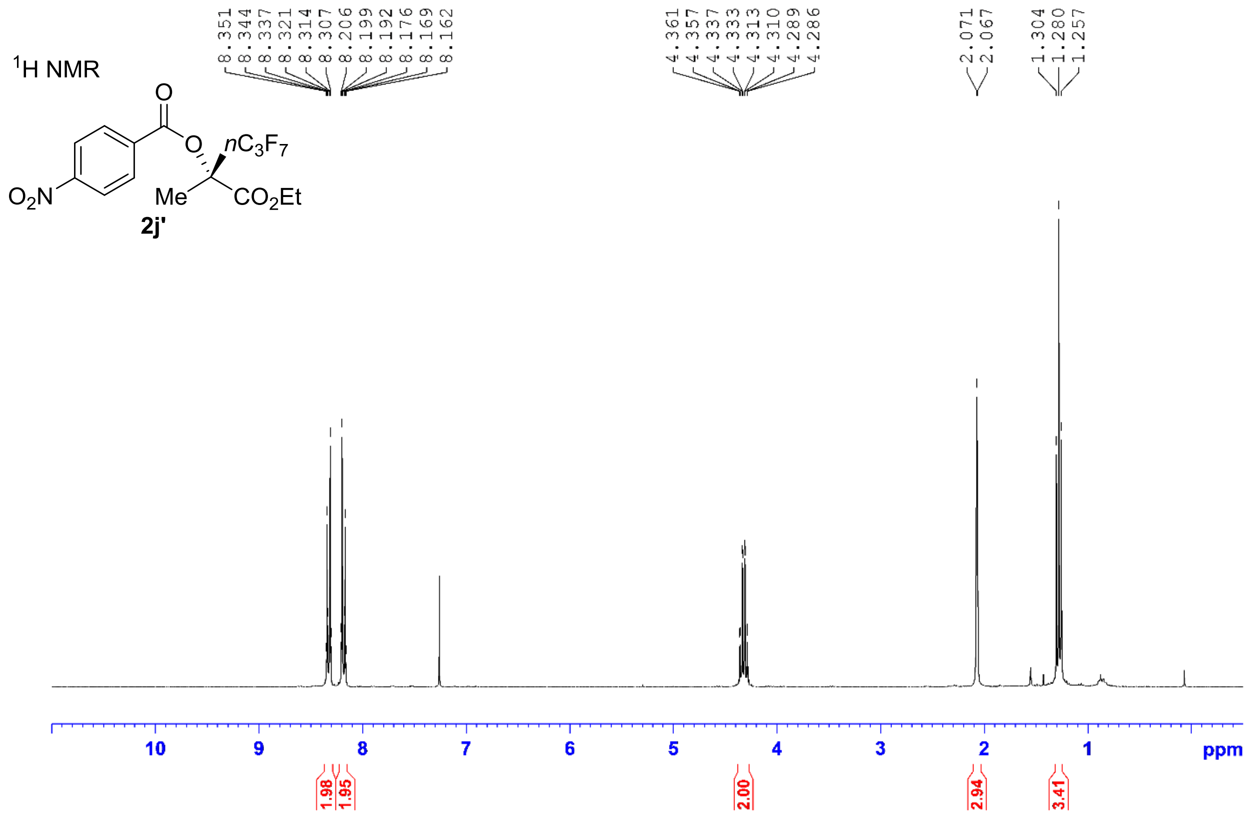

1H NMR
O
O
nC3F7
O2N
Me
CO2Et
2j'
8.351 8.344 8.337 8.321 8.314 8.307 8.206 8.199 8.192 8.176 8.169 8.162
4.361 4.357 4.337 4.333 4.313 4.310 4.289 4.286
2.071 2.067
1.304 1.280 1.257
10 9 8 7 6 5 4 3 2 1 ppm
1.98 1.95 2.00 2.94 3.41

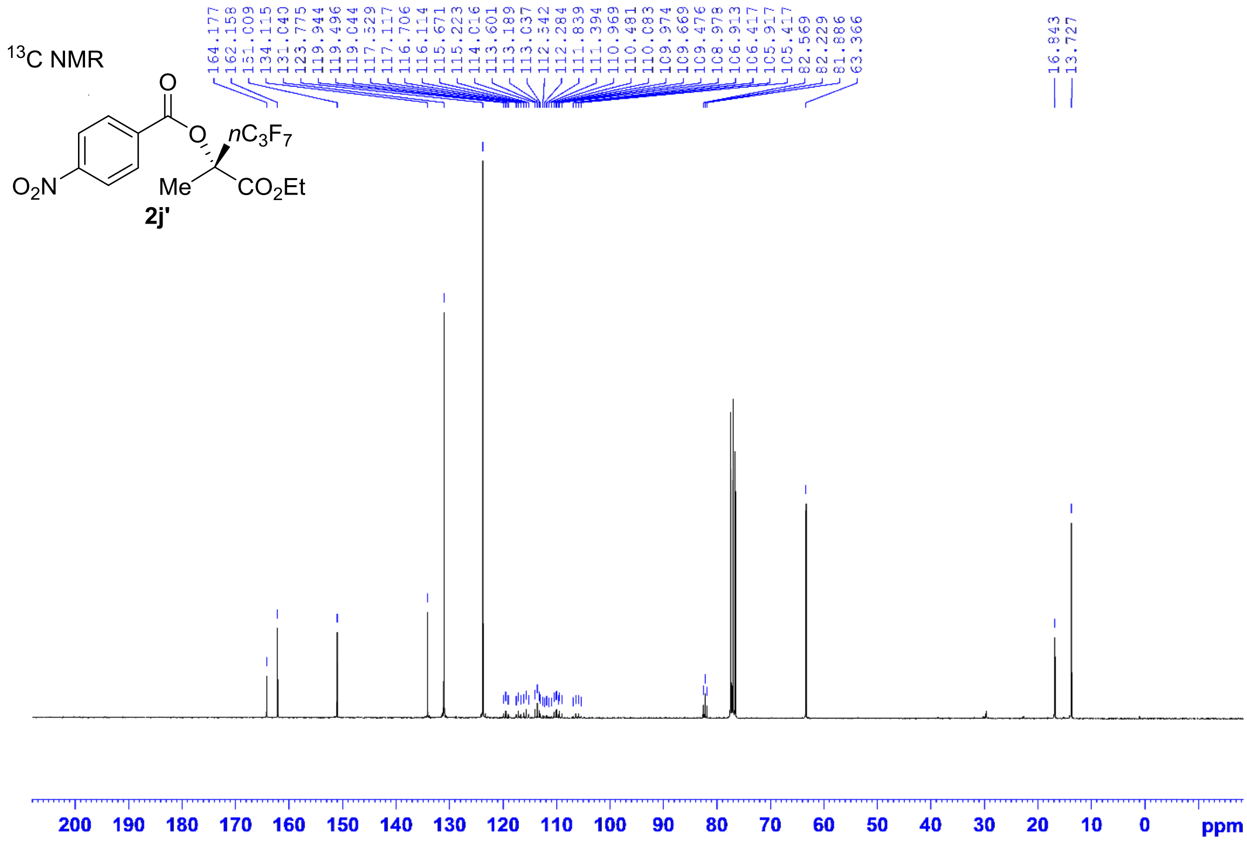

13C NMR
O
O
nC3F7
O2N
Me
CO2Et
2j'
164.177 162.158 151.009 134.115 131.040 123.775 119.944 119.496 119.044 117.529 117.117 116.706 116.114 115.671 115.223 114.016 113.601 113.189 113.037 112.342 112.284 111.839 111.394 110.969 110.481 110.083 109.974 109.669 109.476 108.978 106.913 106.417 105.917 105.417 82.569 82.229 81.886 63.366
16.843 13.727
200 190 180 170 160 150 140 130 120 110 100 90 80 70 60 50 40 30 20 10 0 ppm

^{19}F NMR

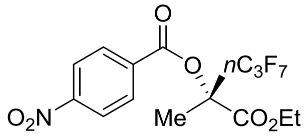

2j'

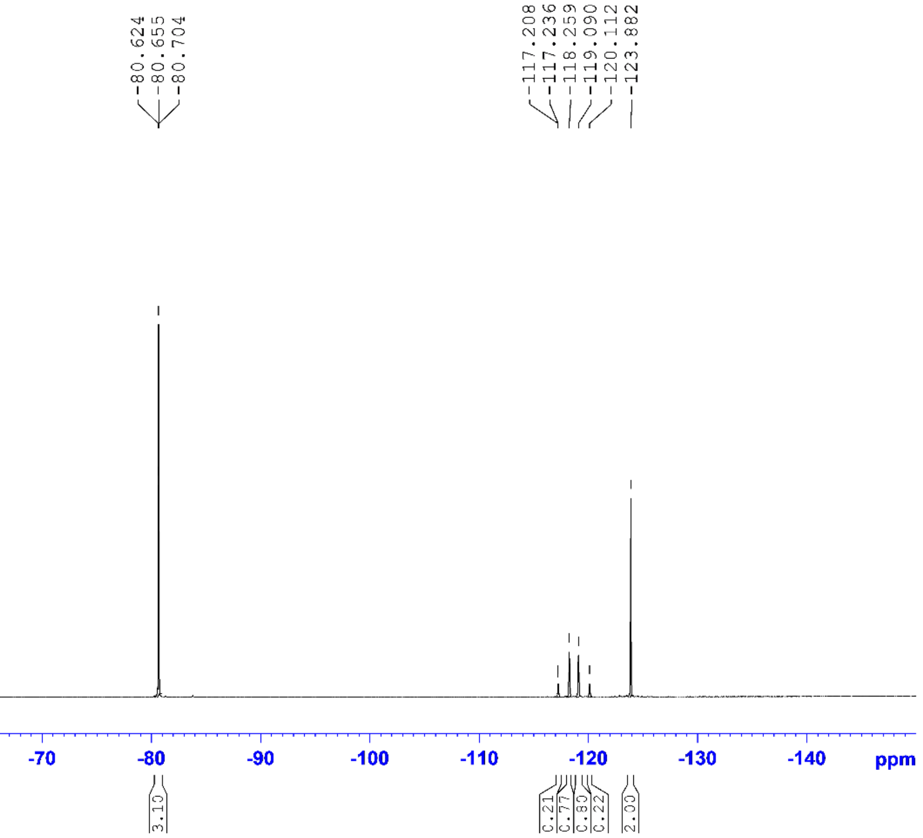

^{1}H NMR

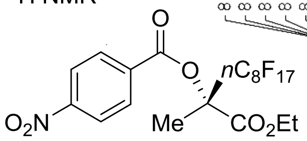

2k'

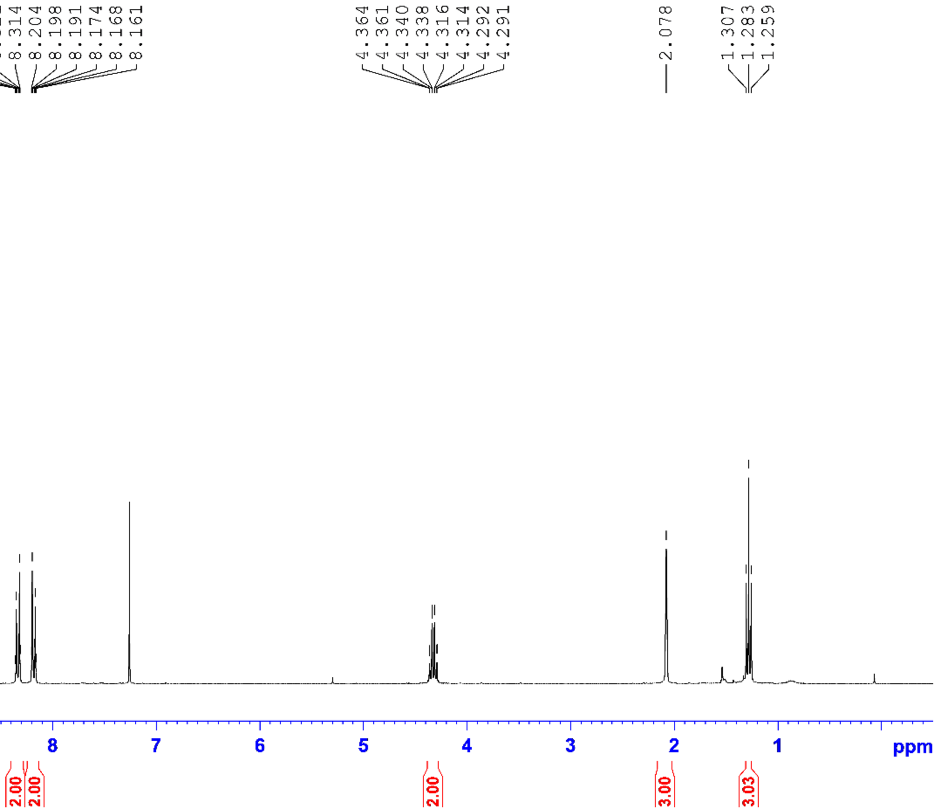

^{13}C NMR

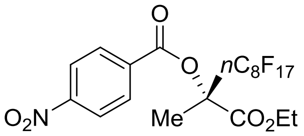

2k'

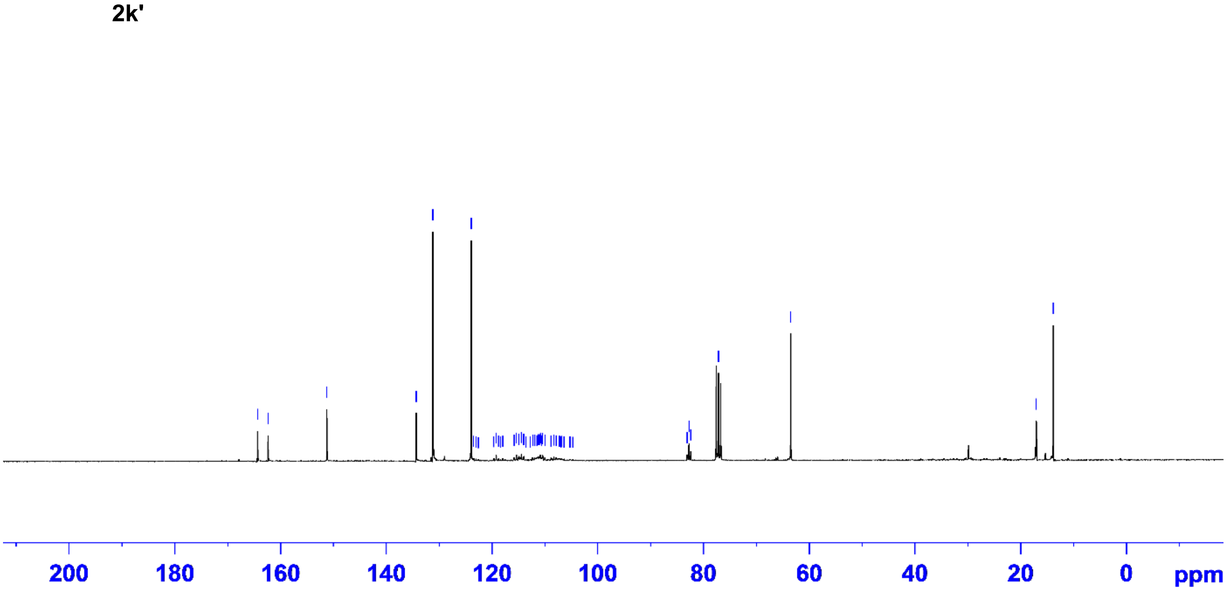

^{19}F NMR

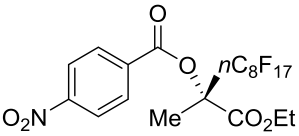

2k'

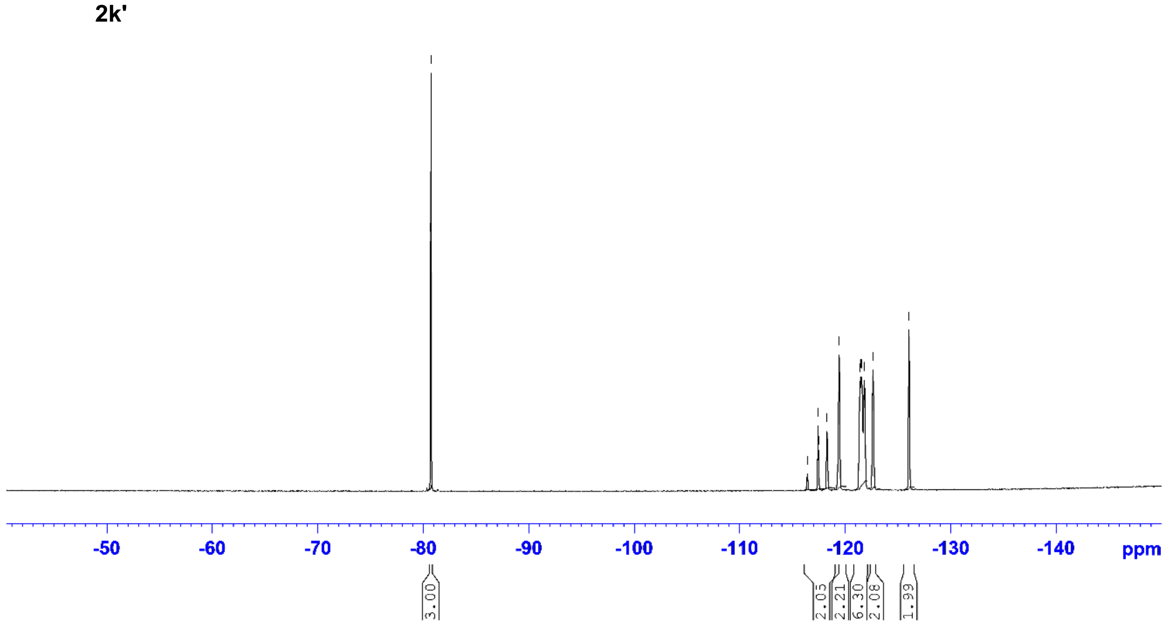

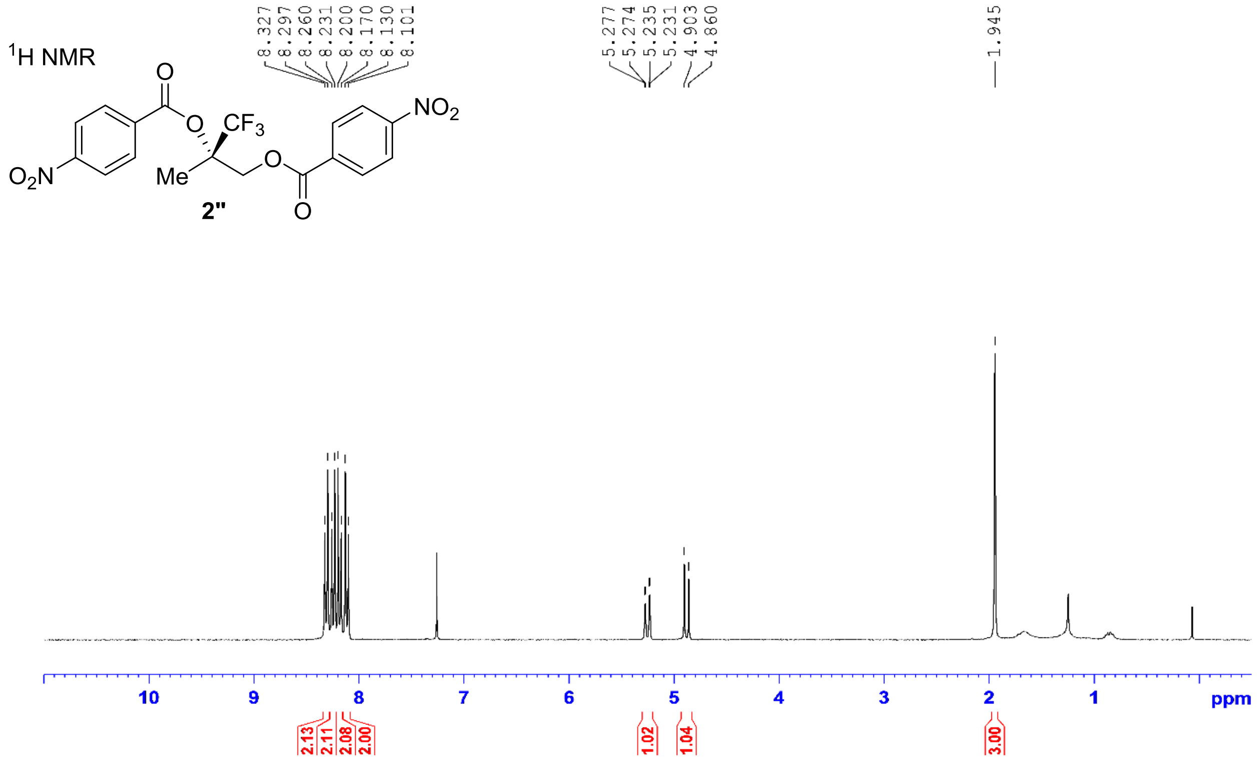

1H NMR
8.327
8.297
8.260
8.231
8.200
8.170
8.130
8.101
5.277
5.274
5.235
5.231
4.903
4.860
1.945
O2N
NO2
CF3
Me
2"
10
9
8
7
6
5
4
3
2
1
ppm
2.13
2.11
2.08
2.00
1.02
1.04
3.00

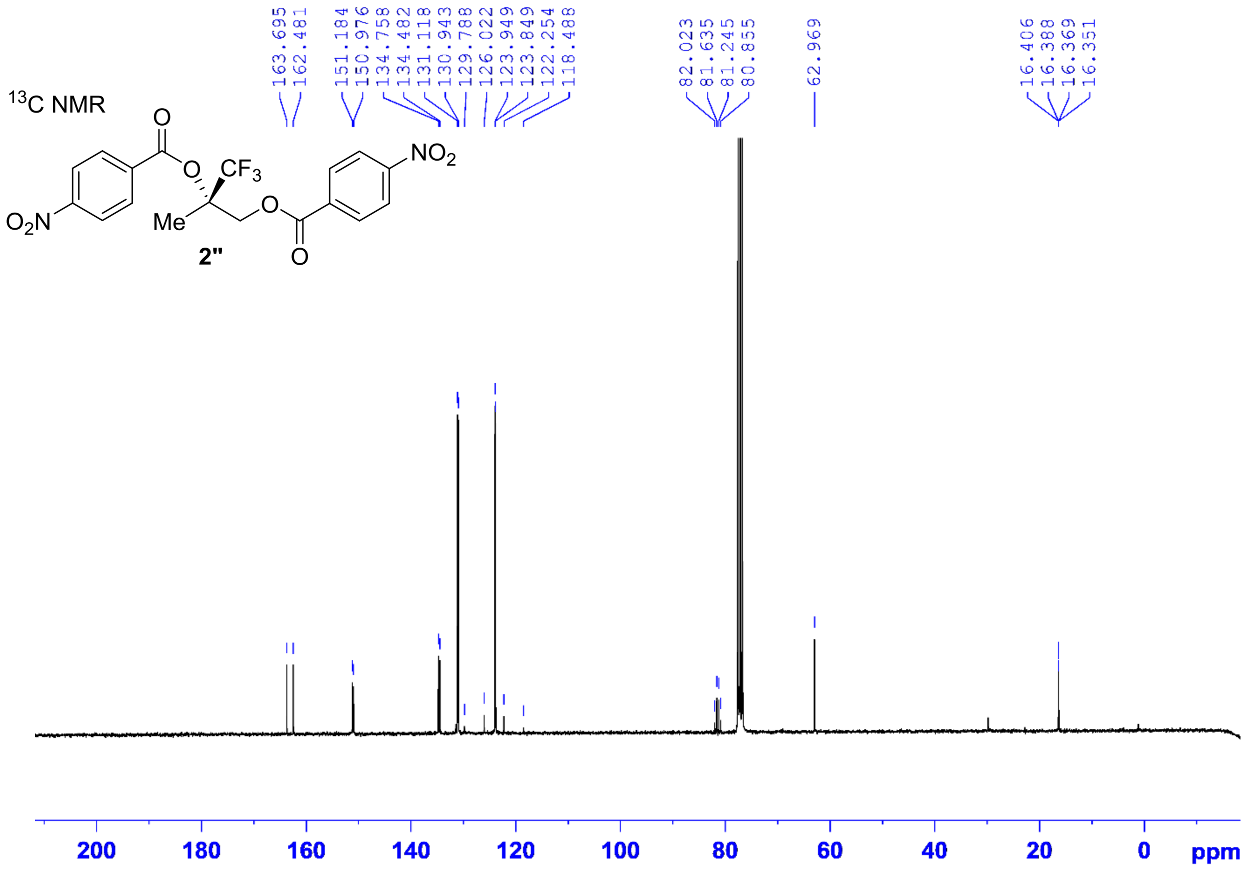

13C NMR
163.695
162.481
151.184
150.976
134.758
134.482
131.118
130.943
129.788
126.022
123.949
123.849
122.254
118.488
82.023
81.635
81.245
80.855
62.969
16.406
16.388
16.369
16.351
O2N
NO2
CF3
Me
2"
200
180
160
140
120
100
80
60
40
20
0
ppm

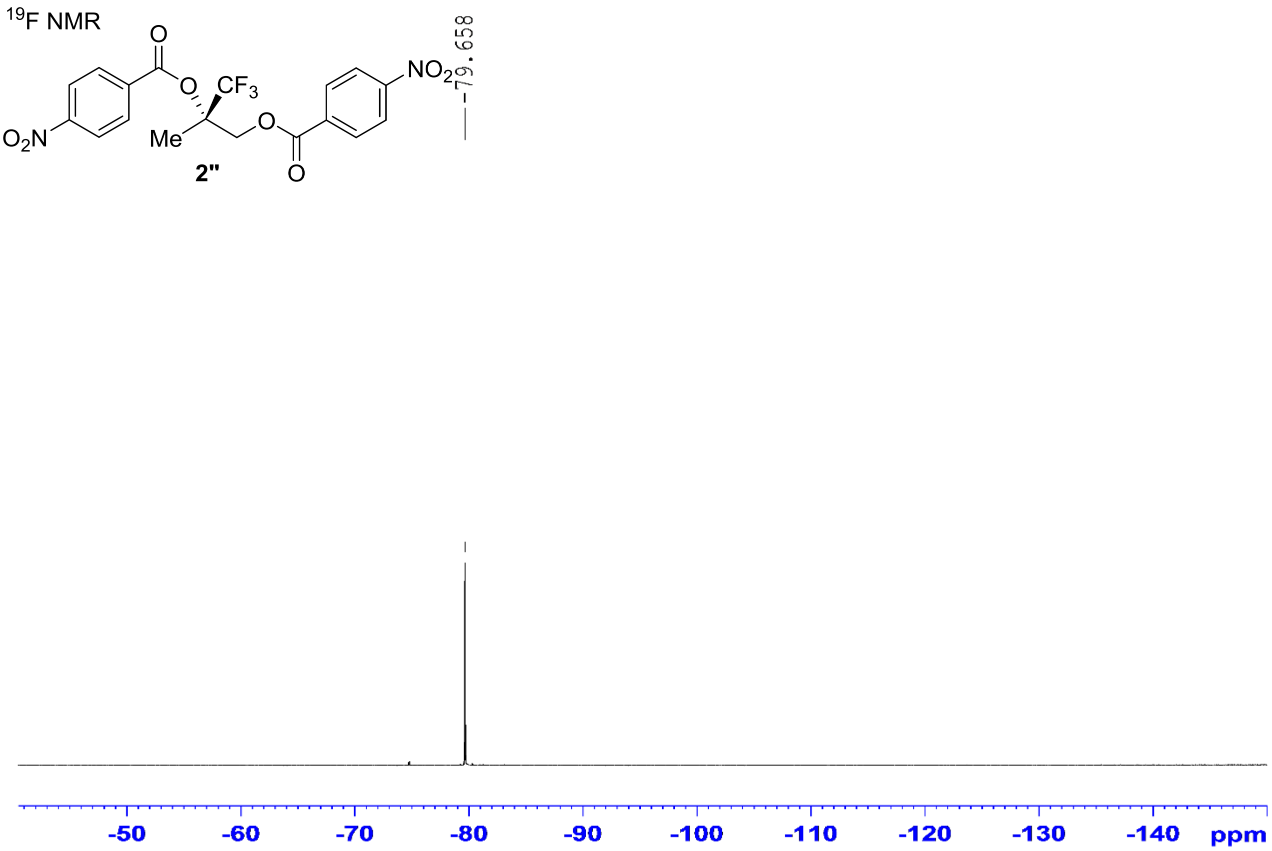

19F NMR
O2N
Me
CF3
NO2
2"
-79.658
-50 -60 -70 -80 -90 -100 -110 -120 -130 -140 ppm

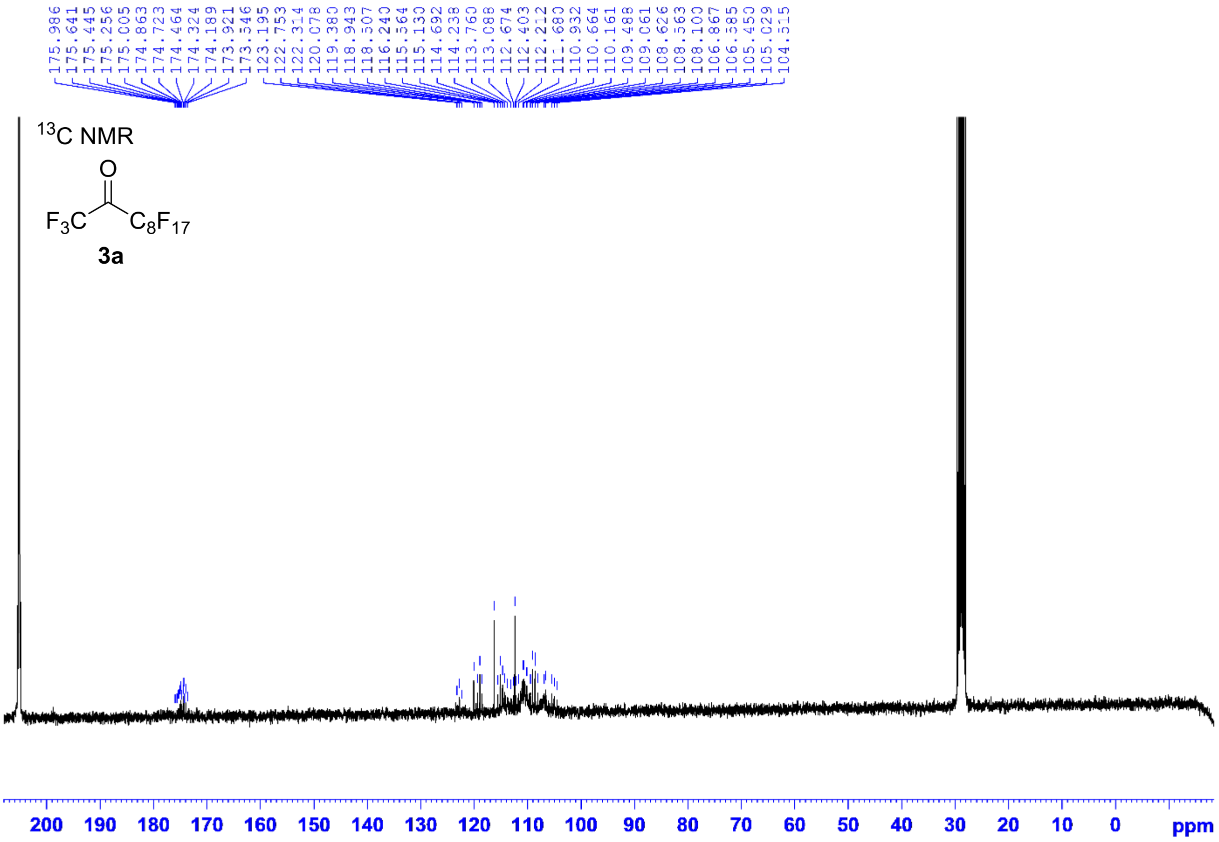

13C NMR
F3C
C8F17
3a
175.986 175.641 175.445 175.256 175.005 174.863 174.723 174.464 174.324 174.189 173.921 173.546 123.195 122.753 122.314 120.078 119.380 118.943 118.507 116.240 115.564 115.130 114.692 114.238 113.760 113.088 112.674 112.403 112.212 111.680 110.932 110.664 110.161 109.488 109.061 108.626 108.563 108.100 106.867 106.385 105.450 105.029 104.515
200 190 180 170 160 150 140 130 120 110 100 90 80 70 60 50 40 30 20 10 0 ppm

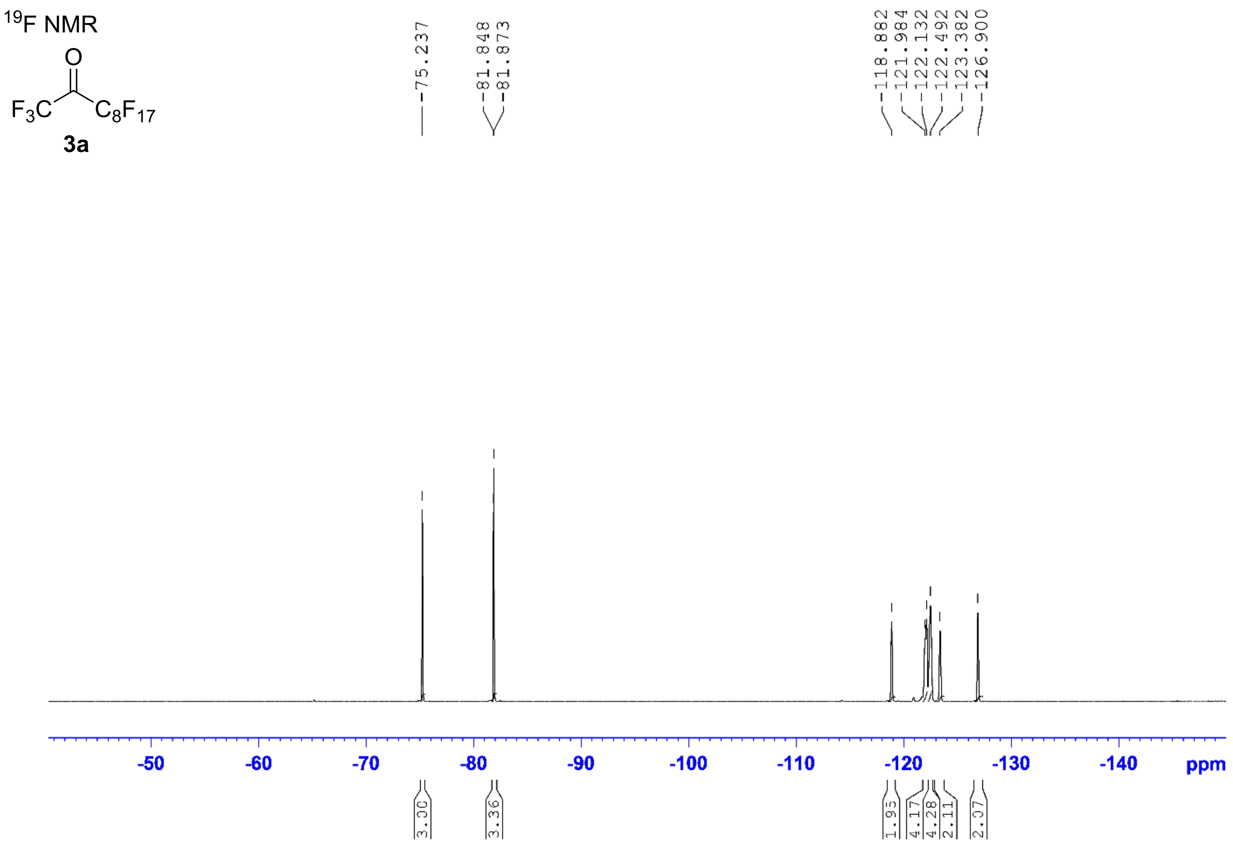

19F NMR
O
F3C
C8F17
3a
-75.237
-81.848
-81.873
-118.882
-121.984
-122.132
-122.492
-123.382
-126.900
-50
-60
-70
-80
-90
-100
-110
-120
-130
-140
ppm
3.00
3.36
1.93
4.17
4.28
2.11
2.07

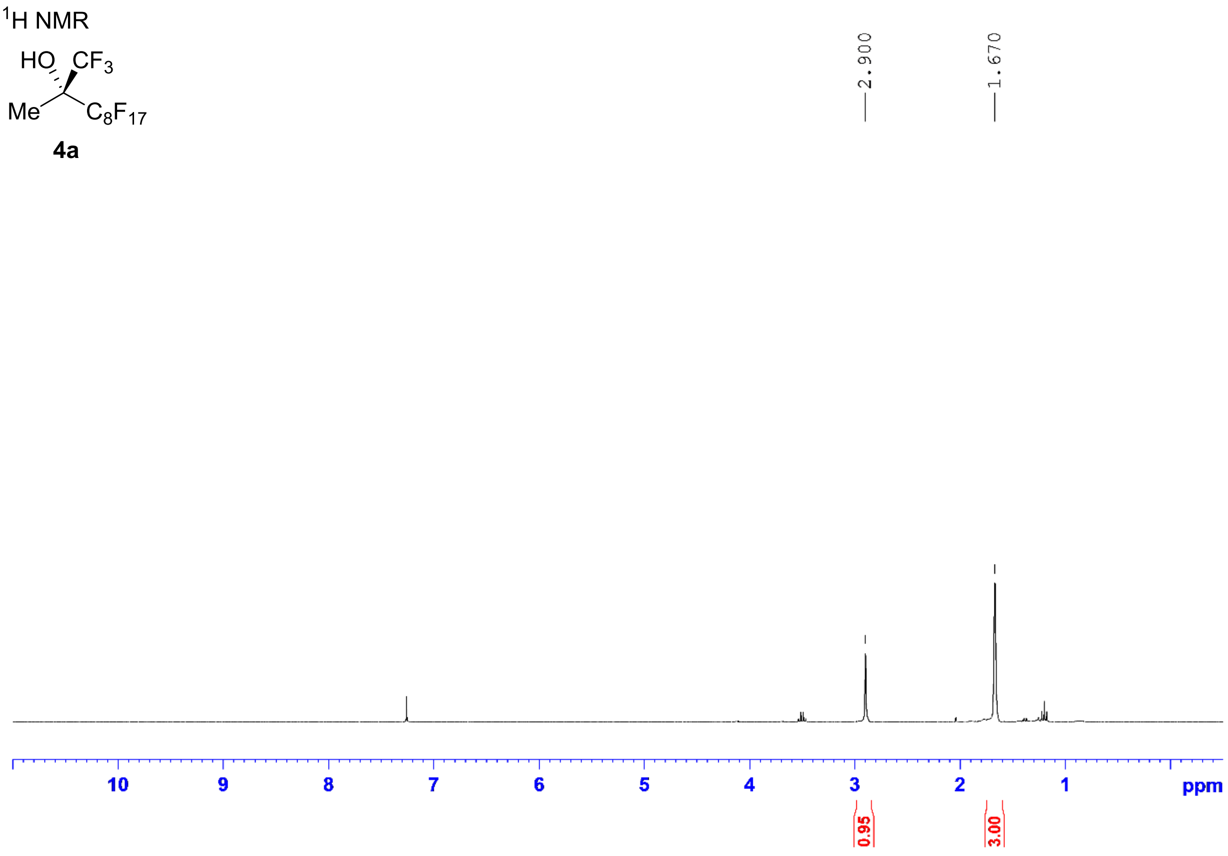

1H NMR
HO
CF3
Me
C8F17
4a
2.900
1.670
10
9
8
7
6
5
4
3
2
1
ppm
0.95
3.00

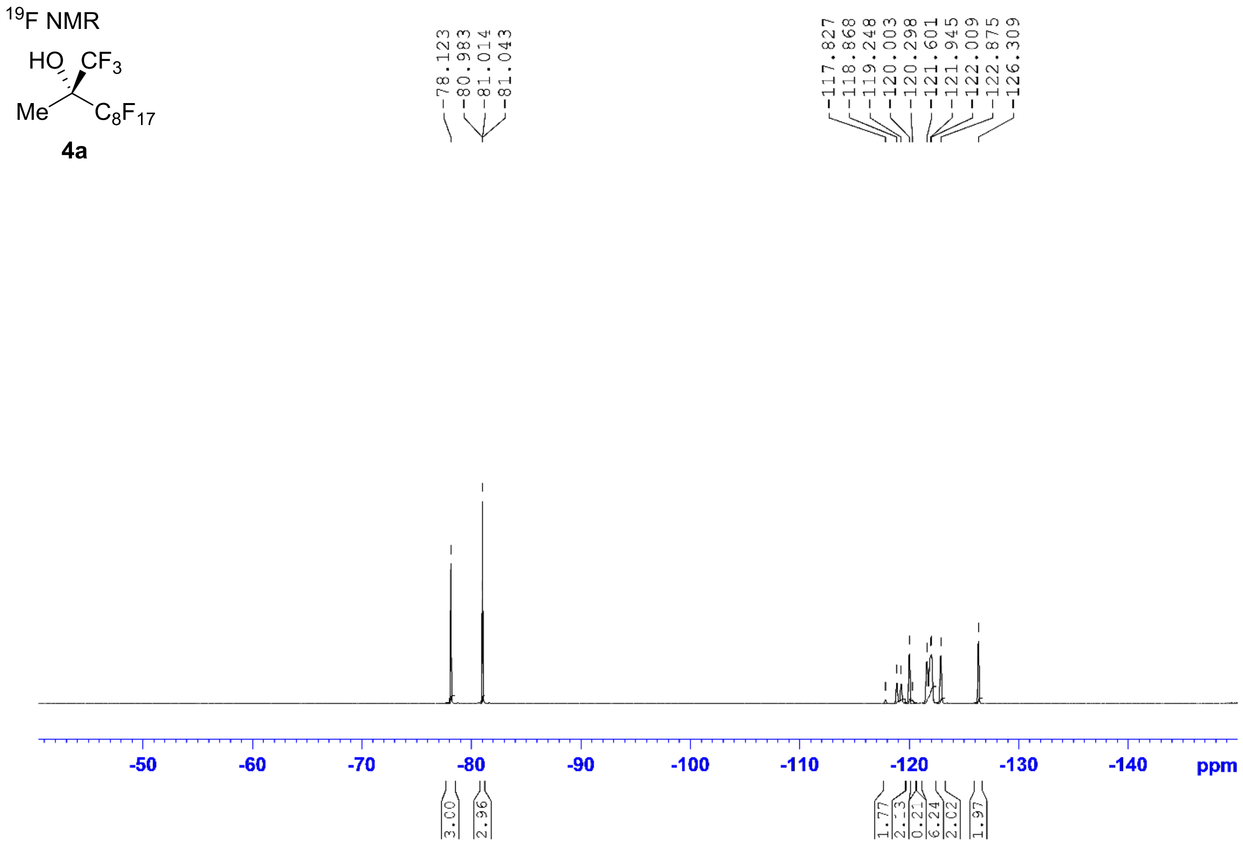
19F NMR
HO CF3
Me C8F17
4a
-78.123
-80.983
-81.014
-81.043
-117.827
-118.868
-119.248
-120.003
-120.298
-121.601
-121.945
-122.009
-122.875
-126.309
-50 -60 -70 -80 -90 -100 -110 -120 -130 -140 ppm

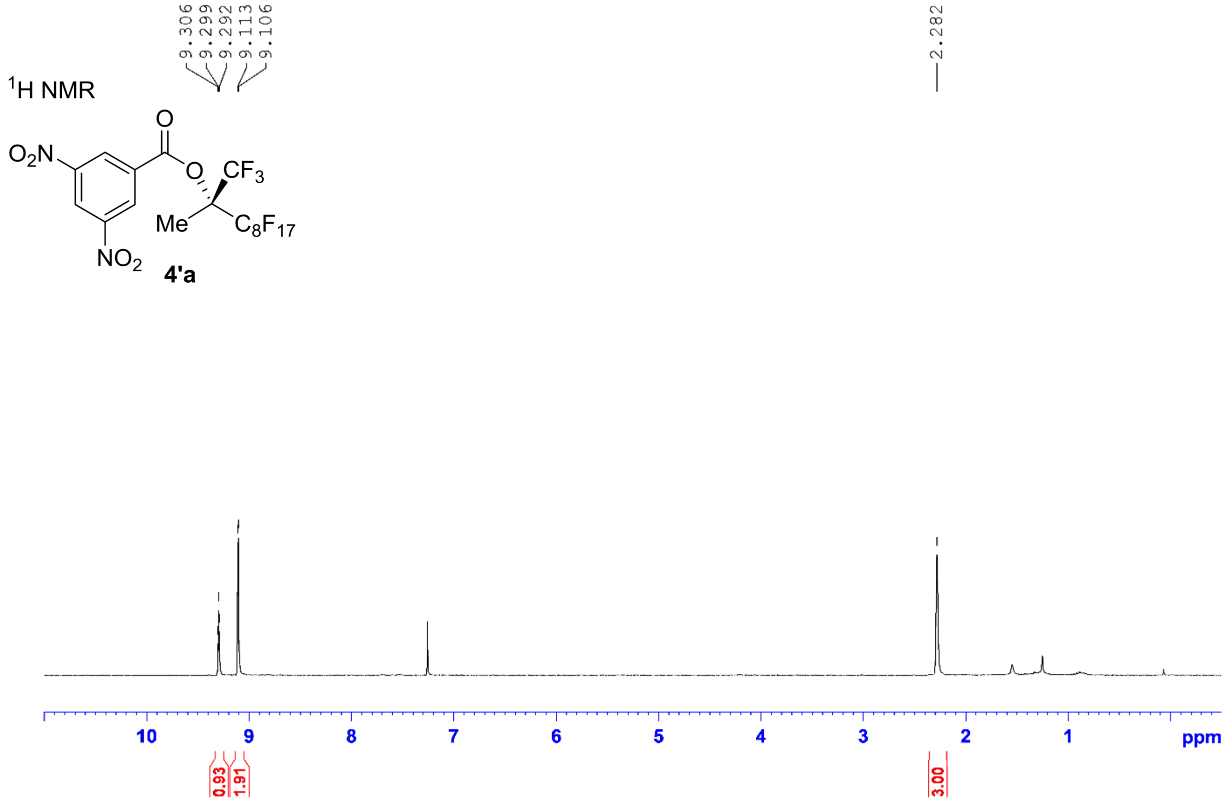
1H NMR
O2N
NO2
O
CF3
Me C8F17
4'a
9.306
9.299
9.292
9.113
9.106
2.282
10 9 8 7 6 5 4 3 2 1 ppm
0.93
1.91
3.00

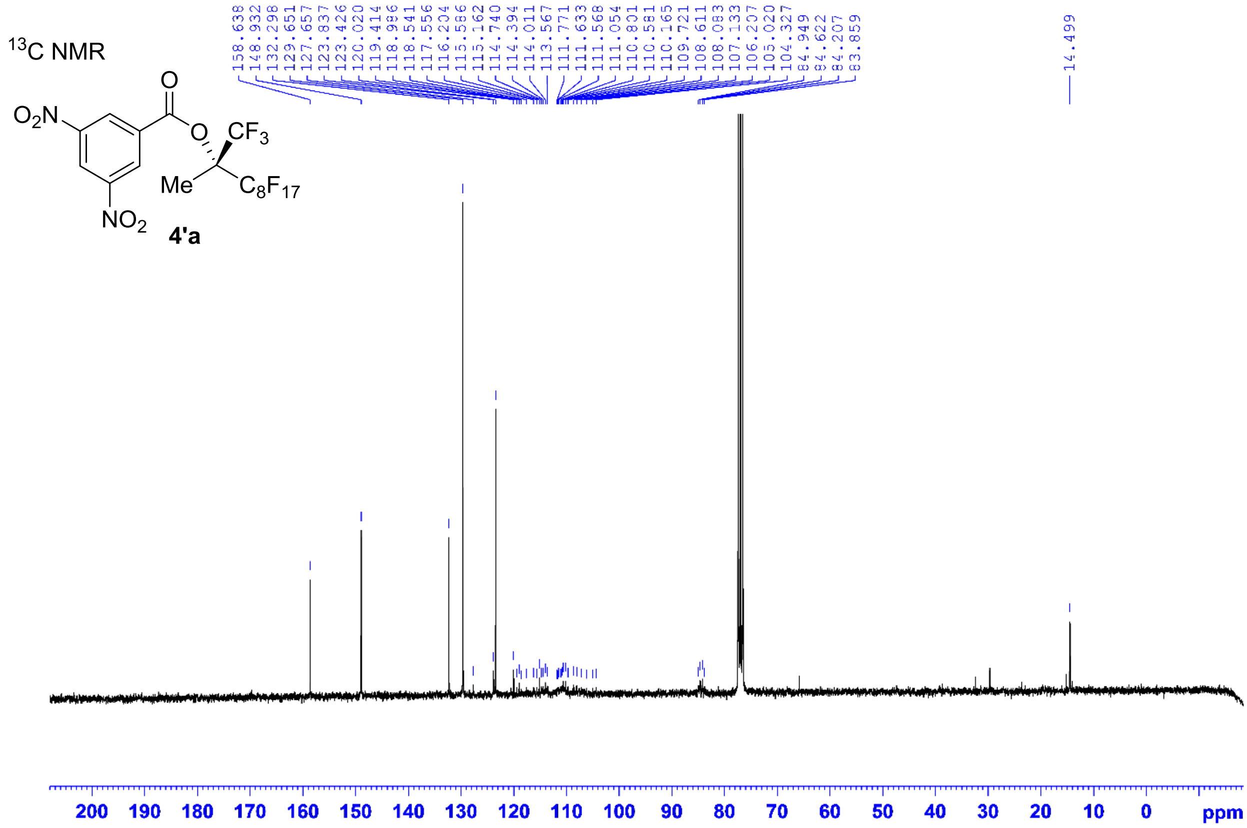
13C NMR
O2N
NO2
CF3
Me
C8F17
4'a
158.638
148.932
132.298
129.651
127.657
123.837
123.426
120.020
119.414
118.996
118.541
117.556
116.204
115.586
115.162
114.740
114.394
114.011
113.567
111.771
111.633
111.568
111.054
110.801
110.581
110.165
109.721
108.611
108.083
107.133
106.207
105.020
104.327
84.949
84.622
84.207
83.859
14.499
200 190 180 170 160 150 140 130 120 110 100 90 80 70 60 50 40 30 20 10 0 ppm

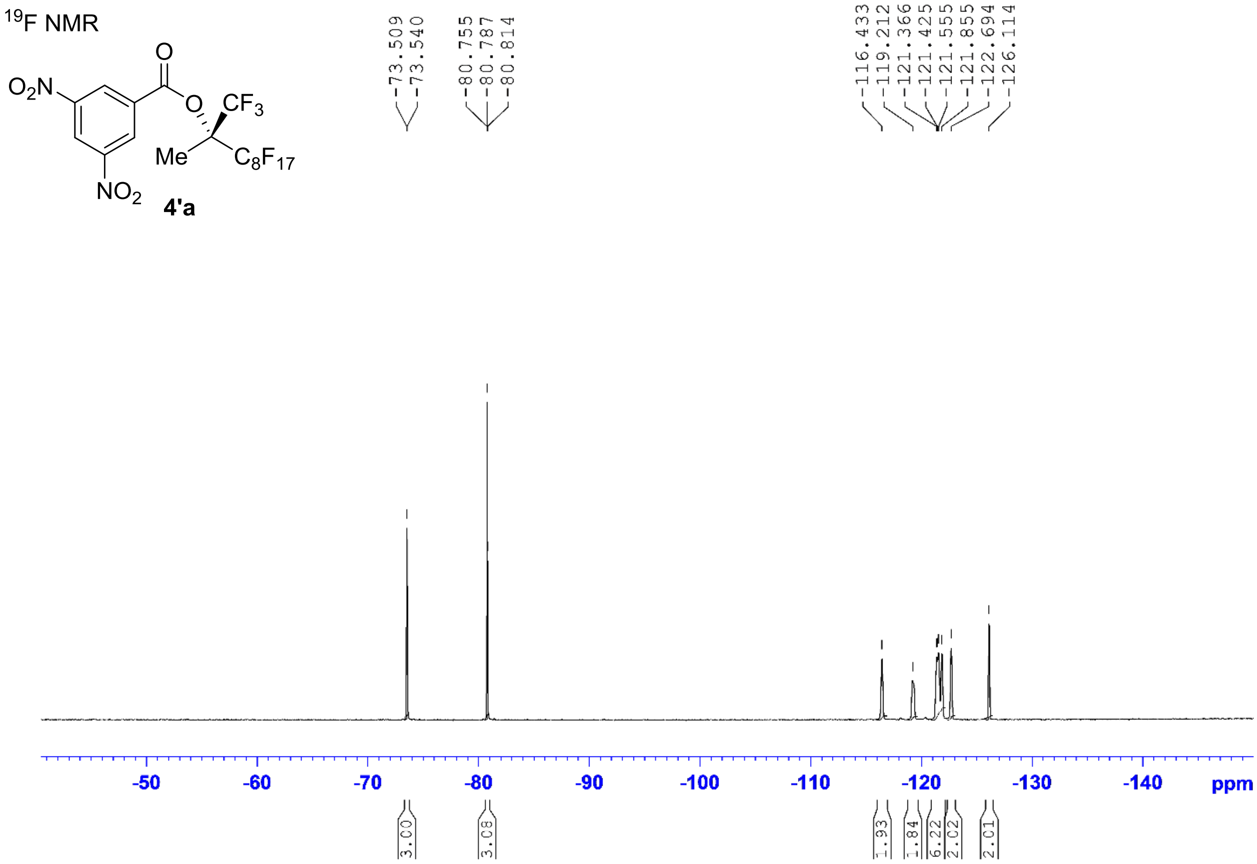
19F NMR
O2N
NO2
CF3
Me
C8F17
4'a
-73.509
-73.540
-80.755
-80.787
-80.814
-116.433
-119.212
-121.366
-121.425
-121.555
-121.855
-122.694
-126.114
-50 -60 -70 -80 -90 -100 -110 -120 -130 -140 ppm
3.00
3.08
1.93
1.84
6.22
2.02
2.01
